# Policy relevant Results from an Expert Elicitation on the Human Health Risks of Decabromodiphenyl ether (decaBDE) and Hexabromocyclododecane (HBCD)

**DOI:** 10.1186/1476-069X-11-S1-S7

**Published:** 2012-06-28

**Authors:** Solveig Ravnum, Karin E Zimmer, Hans Keune, Arno C Gutleb, Albertinka J Murk, Janna G Koppe, Brooke Magnanti, Jan L Lyche, Gunnar S Eriksen, Erik Ropstad, Janneche U Skaare, Michael Kobernus, Aileen Yang, Alena Bartonova, Martin Krayer von Krauss

**Affiliations:** 1 Norwegian Veterinary Institute, P.O.Box 750, 0106 Oslo, Norway; 2 Department of Basic Sciences and Aquatic Medicine, Norwegian School of Veterinary Science, Department of Production Animal Clinical Science, P.O.Box 8146, 0033 Oslo, Norway; 3 Research Institute for Nature and Forest (INBO), Brussels; Centre of Expertise for Environment and Health, Faculty of Political and Social Sciences, University of Antwerp; naXys, Namur Center for Complex Systems, University of Namur, Belgium; 4 Department of Environment and Agro-biotechnologies (EVA), Centre de Recherche Public - Gabriel Lippmann, Department of Environment and Agro-biotechnologies (EVA), 41 rue du Brill, 4422 Belveaux, Grand-Duchy of Luxembourg; 5 Wageningen University, Section of Toxicology, P.O. Box 6700 EA, Wageningen, The Netherlands; 6 Wageningen-IMARES, 1976CP, IJmuiden, The Netherlands; 7 EcoBaby Foundation, Hollandstraat 6, 3634 AT Loenersloot, The Netherlands; 8 University Hospital, Biophysics group, St. Michael’s Hospital, Southwell Street, Bristol BS2, 8EJ, UK; 9 Department of Production Animal Clinical Science, Norwegian School of Veterinary Science, P.O.Box 8146, 0033 Oslo, Norway; 10 Department of Food Safety and Infection Biology, Norwegian School of Veterinary Science, P.O.Box 8146, 0033 Oslo, Norway; 11 NILU - Norwegian Institute of Air Research, P.O.Box 100, 2027 Kjeller, Norway; 12 WHO, Regional Office for Europe, Scherfigs vej 8, 2100 Copenhagen Ø, Denmark

## Abstract

**Aim:**

Apply a recently developed expert elicitation procedure to evaluate the state of the current knowledge of the two brominated flame retardants (BFRs) most commonly used today; decabromo-diphenyl ether (decaBDE) and hexabromocyclododecane (HBCD) and their potential impact on human health in order to support policy considerations. This expert elicitation was organized by the HENVINET (Health and Environment Network) Consortium.

**Method:**

The HENVINET expert elicitation procedure that was used in the evaluations of decaBDE and HBCD is a rapid assessment tool aimed at highlighting areas of agreement and areas of disagreement on knowledge-related key issues for environment and health policy decision making.

**Results:**

The outcome of the expert consultation on BFRs was concrete expert advice for policy makers with specific priorities for further action made clear for both stakeholders and policy makers. The experts were not in agreement whether or not the knowledge currently available on decaBDE or HBCD is sufficient to justify policy actions, but most experts considered that enough data already exists to support a ban or restriction on the use of these compounds. All experts agreed on the necessity of more research on the compounds. Priority issues for further research were, among others:

• more studies on the extent of human exposure to the compounds.

• more studies on the fate and concentration in the human body of the compounds.

## Introduction and aims

HENVINET (Health and Environment Network, http://www.henvinet.eu) was funded by the EU under the 6th Framework Programme, to support the development of integrated health and environmental policies. The aim of the HENVINET consortium was to establish a long-term environment and health network between researchers, stakeholders and policy makers and to make the latest scientific opinions available for society. To achieve these aims the HENVINET consortium was reviewing and disseminating knowledge on environmental health related issues, and the scientific knowledge was processed and interpreted focussing on relevance for policy makers and stakeholders. The HENVINET consortium focussed on four priority health end points as defined in the European Health Action Plan (EHAP); asthma and allergies, cancer, neurodevelopmental disorders and endocrine disruptors.

Two of the chemical substances causing endocrine disruptive effects chosen for evaluation were the major brominated flame retardants (BFRs) in use today; deca brominated diphenyl ether (decaBDE) and hexabromocyclododecane (HBCD). These two chemicals were recently introduced in large quantities in man-made products, and pros and cons for using the chemicals have been heavily debated ever since [1,2]. HENVINET developed an expert elicitation procedure and used it to assess the health and policy implications for several other environmental health issues, including phthalates, another group of chemicals causing endocrine disruptive effects [3]. The method and the experiences from the phthalate evaluation were used to evaluate the health and policy implications of decaBDE and HBCD.

The expert elicitation procedure has been much questioned, but until now it is still considered a valuable complement to science for supporting policy making before sufficient scientific data is available, as it helps highlight areas of uncertainties in the discussed topic [4,5]. Experiences from the HENVINET phthalate project showed that the initial review on phthalates by the project members did not succeed in bringing forward the most important messages to policy makers [6]. For this reason, an expert elicitation procedure was decided upon as a possible tool to reach the policy makers, similar to what was presented in Krayer von Krauss et al., 2008 [7]. An extensive review of the consultation of experts in all four priority areas of the HENVINET consortium, with an overall discussion and analysis of the outcome, was recently made by Keune et al., [8].

We here report the results of the expert elicitations on decBDE and HBCD that were conducted during the spring of 2009. The goal of the elicitations was to identify the most relevant knowledge gaps, the degree of agreement and disagreement on aspects of the assessment of human health effects from these two substances. When relevant, concrete advice for policy makers was included.

## Background

DecaBDE and HBCD are the two major BFRs used to prevent building materials, electronics, clothes and furniture from catching fire [1,9]. The major concerns about decaBDE and HBCD are their persistence in the environment, their potential for bioaccumulation, and indications of toxicological effects.

### DecaBDE

DecaBDE differs from other brominated diphenyl ethers (BDEs) with respect to some important physicochemical properties making it less bioaccumulative and less toxic. In many countries decaBDE has therefore been less strictly regulated than the other BDEs. Even if regarded as less toxic, there are indications of toxicological effects, such as endocrine and neurodevelopmental disturbances [10]. In 2008 decaBDE was therefore banned from being used in electrical and electronic products in the EU [11]. Finally in August 2010 decaBDE was registered under the EU’s REACH Regulation [2]. In Norway decaBDE was totally banned already in 2008 [2]. The states of Maine, Washington, Vermont and Oregon have restricted the use of decaBDE in certain products, but still many major uses of the substance are allowed in North-America [2]. On December 17, 2009, as result of negotiations with the EPA, the two U.S. producers of decaBDE and the largest U.S. importer of decaBDE announced commitments to phase out decaBDE in the United States. Production, import, and sales of decaBDE for most uses in the United States must end by December 31, 2012, and all uses of decaBDE must end by the end of 2013 [12]. The use of Deca-BDE is not subject to any regulatory restrictions in Asia [2].

### HBCD

Concentrations of HBCD in the environment have increased since 2001 [13], probably caused by the increased use of HBCD when replacing other banned or withdrawn BFRs (e.g. Penta BDE and OctaBDE). Indications of toxicological effects are reported for HBCD, such as endocrine disruption effects [9]. Different countries use different policies regarding the production and use of HBCD, but there are hardly any restrictions on the production or use of HBCD today: Canada, Australia and Japan have different national assessments of HBCD under preparation, Ukraine has registered HBCD on their hazard chemical list, and Norway included HBCD in its national action plan for BFRs in 2007 [14]. On June 2^nd^ 2009 the European Chemicals Agency (ECHA) within the REACH framework decided to restrict the use of HBCD within the EU such that it can only be used when “authorized” for specific purposes [15]. Only very recently, on August 18^th^ 2010 the U.S. Environmental Protection Agency (EPA) included HBCD in the EPA’s List of Chemicals of Concern, and they plan to finalise a review of HBCD in 2012 [16,17]. HBCD is currently being reviewed for global agreement of restriction by the Stockholm Convention on Persistent Organic Pollutants [18], and decisions in October 2010 concluded that HBCD should now proceed to the risk management phase, and that a global phase-out can be taken up for consideration in 2013 [14].

Alternative substances to decaBDE and HBCD with putatively lower risks have been proposed [19,20], but knowledge on the potential risks of these compounds are limited and further investigation is required.

## Methodology

Detailed description of the work flow in the expert elicitation procedure that we used to assess the health and policy implications of decaBDE and HBCD can be found in the phthalates study by Zimmer et al., [3], and also in the review by Keune et al., [8].

**Figure 1:**
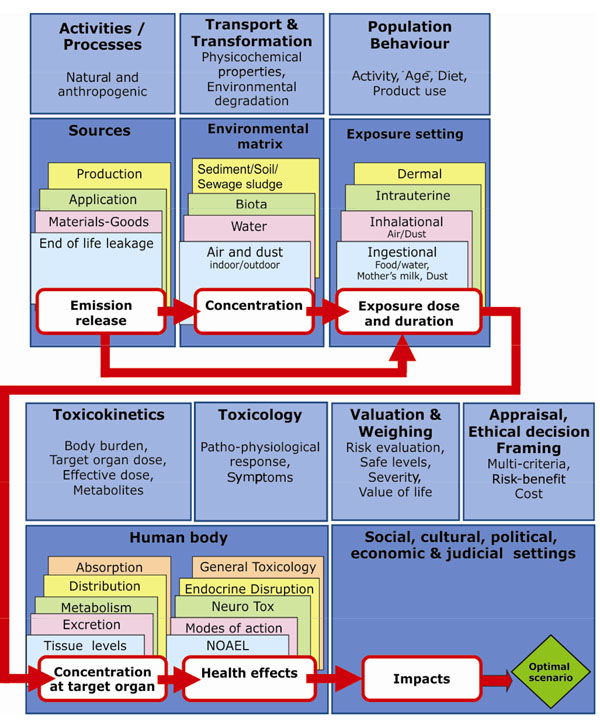
**Cause-effect diagram of decaBDE and HBCD.** The diagram illustrates the scientists current understanding of the cause-relationship between the production and use of decaBDE and HBCD and their potential impact on health, based on existing reviews and recent publications from 2007-2009.

Prior to the expert consultation, certain preparations were necessary: First, we wrote a short background document based on existing reviews and recent publications from 2007-2009, then, a cause-effect diagram was developed to illustrate the scientists’ current understanding of the cause-effect relationship between the production and use of decaBDE and HBCD and their potential impact on health (Figure 1), and finally, an online questionnaire-tool was made [3]. The background documents together with the questionnaires can be found at http://henvinet.nilu.no/EvaluationofKnowledge/tabid/1333/language/en-US/Default.aspx. Login required. The questionnaires are also provided in the additional files 1 and 2.

Thirty-four experts from around the world working in the field of BFRs were asked to fill in the online questionnaire and express their confidence in the current knowledge on the different aspects of decaBDE and HBCD illustrated in the cause-effect diagram. The aim was to identify knowledge gaps and potential areas of agreement or disagreement regarding these issues. The experts were selected based on their publications in the field of decaBDE or HBCD in the last decade. We invited both junior and senior scientists of both genders from around the world. The questionnaire was divided in two parts. In Part A the experts were asked to evaluate the cause-effect diagram. In Part B the questions were connected to all the different elements and sub-elements of the diagram. The experts were asked to tick the box representing their confidence level in the specific topic that the question concerned (Very High = VH, High = H, Medium = M, Low = L, Very Low = VL).

Finally, from these experts a group of eight was asked to fill in a second questionnaire (se additional files 3 and 4) and to attend an expert panel workshop. Selection of the experts was done as in the phthalate study [3], where expertise in at least one of the main topics of the cause-effect diagram (Figure 1) was required. The goal of the second questionnaire was to identify priorities for further action and to discuss the implications of the results of the first questionnaire for policy and research. The experts were asked to pinpoint priority elements in the cause-effect diagram. Based on the results of both questionnaires, the expert panel workshop identified further action to be taken, such as political decision or further research. The final aim was to arrive at concrete policy advice for policy makers. The expert elicitation procedure was also discussed and judged by the experts.

As a final step, policy briefs were prepared on the current gaps of knowledge and expert opinion of decaBDE and HBCD and the results of this HENVINET expert elicitation on decaBDE and HBCD (“HENVINET policy brief on decaBDE” and “HENVINET policy brief on HBCD” https://henvinet.nilu.no, also provided in the additional files 5 and 6).

## Results and discussion

**Figure 2:**
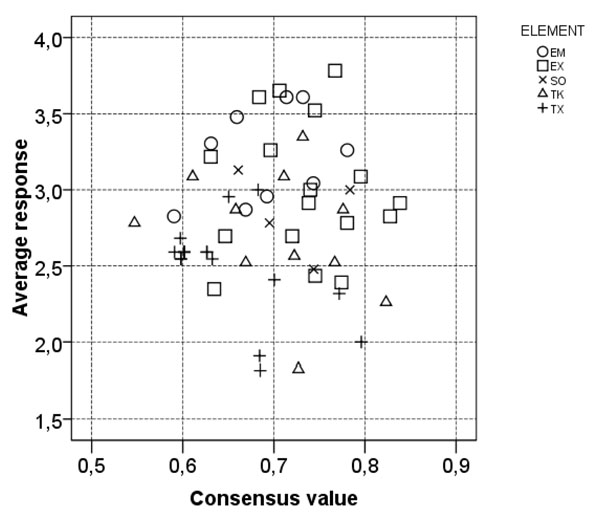
**Consensus scores and average confidence scores for each question from the first decaBDE questionnaire.** The average confidence scores are calculated assigning the answer categories ordinal values (VH=5, H=4, M=3, L=2, VL=1). The question belonging to the same diagram element box are indicated by the same symbol. EM=Environmental Matrix, EX=Exposure, SO=Source, TK=Toxicokinetics, TX=Toxicity).

**Figure 3:**
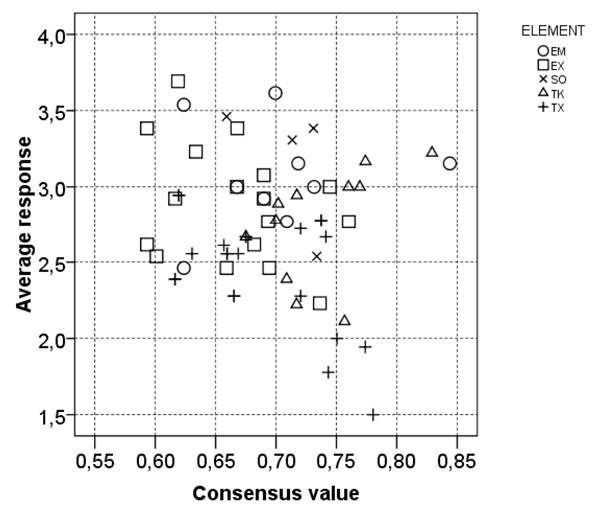
**Consensus scores and average confidence scores for each question from the first HBCD questionnaire** The average confidence scores were calculated assigning the answer categories ordinal values (VH=5, H=4, M=3, L=2, VL=1). The question belonging to the same diagram element box are indicated by the same symbol. EM=Environmental Matrix, EX=Exposure, SO=Source, TK=Toxicokinetics, TX=Toxicity).

The results from expert evaluation of the first questionnaire are provided in the additional file 7. Selected parts of the results from questionnaire 1 are discussed below and presented in Figure 2 &3 and in Table 1 & 2. Results from questionnaire 2 are presented in Table 3.

**Table 1:** Selected questions on decaBDE that scored high or low (outside the 10-90 percentile range) in the average confidence score (Mean) (a) or in consensus (CNS) (b).

(a)											
**Deca**	Questions	VH	H	M	L	VL	No. Resp.	Mean	Std	CNS	RANK (CONS)

		**5**	**4**	**3**	**2**	**1**					

	Environmental Matrix										
**EM3**	**Sediments**	**2**	**13**	**5**	**3**	**0**	**23**	**3.61**	**0.69**	**0.71**	**25**
**EM4**	**Sewage sludge**	**2**	**12**	**7**	**2**	**0**	**23**	**3.61**	**0.84**	**0.73**	**20**
**EM5**	**Soil**	**3**	**9**	**7**	**4**	**0**	**23**	**3.48**	**0.78**	**0.66**	**39**
	Level of exposure										
**EX2**	**Occupationally exposed**	**1**	**12**	**8**	**2**	**0**	**23**	**3.52**	**0.73**	**0.75**	**15**
	Main sources of exposure										
**EX5**	**Occupationally exposed**	**3**	**13**	**6**	**1**	**0**	**23**	**3.78**	**0.74**	**0.77**	**12**
	Occupational exposed										
**EX11**	**Inhalation**	**4**	**8**	**10**	**1**	**0**	**23**	**3.65**	**0.83**	**0.71**	**27**
	Infants and children										
**EX17**	**Via breast milk**	**2**	**14**	**4**	**2**	**1**	**23**	**3.61**	**0.94**	**0.68**	**34**
	Toxicokinetics										
**TK10**	**Final metabolite concentration in target tissues**	**0**	**1**	**3**	**10**	**9**	**23**	**1.83**	**0.83**	**0.73**	**22**
	Human Epidemiological studies										
**TX01**	**Males**	**0**	**1**	**5**	**7**	**9**	**22**	**1.91**	**0.92**	**0.68**	**33**
**TX02**	**Females**	**0**	**1**	**4**	**7**	**10**	**22**	**1.82**	**0.91**	**0.69**	**32**
	Knowledge of the mechanisms of actions										
**TX16**	**Metabolites of BDE209**	**0**	**2**	**1**	**14**	**5**	**22**	**2.00**	**0.82**	**0.80**	**4**

(b)											

**Deca**	Questions	VH	H	M	L	VL	No.Resp.	Mean	Std	CNS	RANK (CONS)

		**5**	**4**	**3**	**2**	**1**					

	Environmental Matrix										
**EM6**	**Water**	**2**	**4**	**8**	**6**	**3**	**23**	**2.83**	**0.95**	**0.59**	**57**
	Level of exposure										
**EX1**	**The general population**	**0**	**2**	**15**	**6**	**0**	**23**	**2.83**	**0.58**	**0.83**	**2**
	General population										
**EX9**	**Ingestion**	**1**	**4**	**14**	**4**	**0**	**23**	**3.09**	**0.73**	**0.80**	**5**
	Occupational exposed										
**EX10**	**Direct contact dermal**	**0**	**3**	**15**	**5**	**0**	**23**	**2.91**	**0.60**	**0.84**	**1**
	Toxicokinetics										
**TK04**	**Debrominated or metabolized by the intestinal microflora**	**0**	**0**	**7**	**15**	**1**	**23**	**2.26**	**0.54**	**0.82**	**3**
**TK07**	**Excreted via urine**	**2**	**5**	**5**	**8**	**3**	**23**	**2.78**	**1.20**	**0.55**	**58**
	Neurodevelopment										
**TX05**	**Males exposed during fetal or neonatal life**	**2**	**2**	**8**	**7**	**3**	**22**	**2.68**	**1.13**	**0.60**	**55**
**TX06**	**Females exposed during fetal or neonatal life**	**2**	**1**	**9**	**6**	**4**	**22**	**2.59**	**1.14**	**0.59**	**56**
	Reproductive function in										
**TX13**	**Males exposed during foetal or neonatal life**	**1**	**3**	**7**	**7**	**4**	**22**	**2.55**	**1.10**	**0.60**	**53**
**TX14**	**Females exposed during foetal or neonatal life**	**1**	**3**	**7**	**7**	**4**	**22**	**2.55**	**1.10**	**0.60**	**54**
	Knowledge of the mechanisms of actions										
**TX16**	**Metabolites of BDE209**	**0**	**2**	**1**	**14**	**5**	**22**	**2.00**	**0.82**	**0.80**	**4**

The questions were “What is your level of confidence in the quality of the current scientific data on…” or “What is your level of confidence in the scientists’ ability to predict…” followed by the text in the left side of the table. VH, H, M, L, VL = levels of confidence; No. Resp. = number of respondents; Std = standard deviation; Mean = arithmetic mean of confidence score; CNS = consensus score (agreement); RANK (CNS) = consensus rank.

### Questionnaire 1 (Q1)

Twenty-three and eighteen experts responded to decaBDE Q1 and the HBCD Q1 respectively. Fewer responses for HBCD are due to technical problems - some of the answers were lost.

#### DecaBDE

Part A - Evaluation by the experts of the cause-effect diagram (Figure 1), pointed out four issues. 1) The diagram lacks the effects caused by the degradation products of decaBDE. This should be in focus, as environmental degradation of decaBDE results in lower brominated BDEs that are known to be toxic [21-23]. 2) The last box in the diagram (“Social, cultural, political, economical and judicial settings”) was thought to be unclear. 3) Important health effects including cancer, reproduction, and multiple organ should be added to the toxicology part, and biota, biosphere, and food stuff should be added to the environmental matrix part. 4) Food processing should be a separate element, since there is a suspicion that food is contaminated by decaBDE while processed. We updated the diagram to fit the suggestions from the experts.

Part B - Evaluation of the individual parameters of the cause-effect diagram. The results are illustrated in Figure 2 and Table 1.

There was *very high confidence in knowledge* of certain areas of exposure and environmental matrix: the level of exposure of occupationally exposed, the main sources of exposure of occupationally exposed, the occupational exposure through inhalation, the exposure of infants and children via breast milk, and the concentrations of decaBDE in environmental matrix of sediments, sewage sludge, and soil. Data on these topics have been thoroughly reviewed by Frederiksen et al., [24] and Law et al., [13] and confirm the good confidence level.

Experts expressed *very low confidence in knowledge* in certain toxicity issues such as human epidemiological studies and the mechanisms of actions of metabolites of BDE209,, because there is insufficient data. This was also concluded by the US EPA [10]. Some studies on the mechanisms of action show interaction of decaBDE with the cholinergic system and with the steroid hormone homeostasis [25,26], but the metabolites of BDE209 have not been specifically studied.

*Very low confidence in knowledge* in toxicokinetics was largely related to the lack of knowledge about the final metabolite concentration in target tissues. Relatively high concentrations of decaBDE have been measured in blood and liver [10], but the final metabolite concentration in the target tissue is not easy to quantify. This uncertainty about final metabolite concentrations can be explained by: 1) *in vivo* debromination of decaBDE to toxic lower BDEs [10,27], or by; 2) differences in absorption and excretion of decaBDE compared to other lower brominated diphenyl ethers [28].

The experts *disagreed most* in areas where only a handful of scientific studies exist. These topics were: 1)neurodevelopment toxicity in females and males exposed during foetal or neonatal life, an issue much debated recently[8,10,29]. BDEs have been reported to interfere with the thyroxin level that can play a crucial role in brain development, and recently real-life scenario studies of neurodevelopment in animals with lower dosages of decaBDE were done that showed neurobehavioural effects [28,30]2) reproductive function toxicity in females and males exposed during foetal or neonatal life, 3) toxicokinetics concerning to what extent BDE 209 is excreted via urine, and 4) the environmental matrix issue of the concentration of decaBDE in water.

#### HBCD

Part A - Evaluation of the cause-effect diagram (Figure 1). Two issues in need of specific focus were identified. 1) The analytical techniques GC/MS and LC/MS for measuring concentrations of HBCD differ and the results cannot be compared [13,31]. 2) The differences in behaviour of each HBCD diastereoisomer need more focus. Recent studies on animals show that isomers of HBCD are selectively metabolised and enriched in the body [9].

The experts further suggested that more health-effect endpoints were included in the toxicology part, as well as food and biota in the environmental matrix part. We updated the diagram to fit the suggestions.

Part B - Evaluation of the individual parameters of the cause-effect diagram. The results (Figure 3 and Table 2) reflect the outcome of scientific studies that are available in literature. For example, the *low confidence in knowledge* and *high agreement* among experts is related to the lack of knowledge of the mechanism of action of α-HBCD, β-HBCD, γ-HBCD, and other potential metabolites of HBCD. There are reports on toxic effects on the liver system and the thyroid system by HBCD [32-34], but there is very little knowledge on differences between the diastereoisomers, as the technical mixture contains all three. The *high agreement* on medium confidence of knowledge in the toxicokinetics issues corresponds with the published data as reviewed by the European Commission [9].

**Table 2:** Selected questions on HBCD that scored high or low (outside the 10-90 percentile range) in the average confidence score (Mean) (a) or in consensus (CNS) (b).

a)											
**HBCD**	Questions	VH	H	M	L	VL	No. Resp.	Mean	Std	CNS	RANK (CONS)

		**5**	**4**	**3**	**2**	**1**					

	Environmental Matrix										
**EM03**	**Sediments**	**2**	**5**	**5**	**1**	**0**	**13**	**3.62**	**0.87**	**0.70**	**30**
**EM04**	**Sewage sludge**	**2**	**6**	**2**	**3**	**0**	**13**	**3.54**	**1.05**	**0.62**	**53**
	Main sources of exposure										
**EX05**	**Occupationally exposed**	**4**	**2**	**6**	**1**	**0**	**13**	**3.69**	**1.03**	**0.62**	**57**
	Knowledge of the mechanisms of actions										
**TX18**	**a- HBCD**	**0**	**2**	**1**	**10**	**5**	**18**	**2.00**	**0.91**	**0.75**	**10**
**TX19**	**b-HBCD**	**0**	**1**	**1**	**9**	**7**	**18**	**1.78**	**0.81**	**0.74**	**12**
**TX20**	**g-HBCD**	**0**	**1**	**2**	**10**	**5**	**18**	**1.94**	**0.80**	**0.77**	**5**
**TX21**	**Other metabolites of HBCD**	**0**	**0**	**1**	**7**	**10**	**18**	**1.50**	**0.62**	**0.78**	**3**

(b)											

HBCD	Questions	VH	H	M	L	VL	No. Resp.	Mean	Std	CNS	RANK (CONS)

		**5**	**4**	**3**	**2**	**1**					

	Environmental Matrix										
**EM01**	**Debromination & biological halflives**	**0**	**3**	**9**	**1**	**0**	**13**	**3.15**	**0.55**	**0.84**	**1**
	Occupational exposed										
**EX11**	**Inhalation**	**3**	**2**	**5**	**3**	**0**	**13**	**3.38**	**1.12**	**0.59**	**63**
**EX12**	**Ingestion**	**1**	**3**	**4**	**4**	**1**	**13**	**2.92**	**1.12**	**0.62**	**58**
	Infants and children										
**EX14**	**Inhalation**	**1**	**1**	**4**	**5**	**2**	**13**	**2.54**	**1.13**	**0.60**	**61**
**EX16**	**Via food**	**0**	**3**	**5**	**2**	**3**	**13**	**2.62**	**1.12**	**0.59**	**62**
	Toxicokinetics										
**TK05**	**Accumulating in the body**	**0**	**6**	**9**	**3**	**0**	**18**	**3.17**	**0.71**	**0.77**	**4**
**TK06**	**Excreted via bile and faeces**	**0**	**5**	**8**	**5**	**0**	**18**	**3.00**	**0.77**	**0.77**	**6**
**TK08**	**Distribution to different tissues**	**0**	**5**	**12**	**1**	**0**	**18**	**3.22**	**0.55**	**0.83**	**2**
	Human Epidemiological studies										
	Nervous system										
**TX05**	**Males exposed as adults**	**1**	**2**	**3**	**9**	**3**	**18**	**2.39**	**1.09**	**0.62**	**59**
**TX06**	**Females exposed as adults**	**1**	**2**	**3**	**9**	**3**	**18**	**2.39**	**1.09**	**0.62**	**60**

The questions were “What is your level of confidence in the quality of the current scientific data on…” or “What is your level of confidence in the scientists’ ability to predict…” followed by the text in the left side of the table. VH, H, M, L, VL = levels of confidence; No. Resp. = number of respondents; Std = standard deviation; Mean = arithmetic mean of confidence score; CNS = consensus score (agreement); RANK (CNS) = consensus rank.

Surprisingly, in some topics we discovered strong disagreements that did not match the findings in recently published literature. In particular, the experts *highly disagreed* in our confidence of knowledge in toxicity of the nervous system in males and females exposed as adults. Some studies exist on the topic, indicating developmental neurotoxic effects in rats [35] and humans [36] and also neurotransmitter effects on cell systems [37-39]. There is a clear disagreement among the expert scientists whether this data is sufficient knowledge or not. Furthermore, there was *high disagreement* in our confidence in knowledge in some topics of exposure: occupational exposure, and exposure of infants and children via food and inhalation, even though no real studies exist. There are only some estimated data available on these exposure issues, reviewed by the European Commission [9], so it is uncertain why some experts consider us to have high confidence in knowledge on these topics.

### Questionnaire 2 (Q2) and the expert panel workshop

#### Priority areas for DecaBDE

The top three areas to study further in order to assess the health risk of decaBDE were identified (Table 3a). *First*, better understanding of the magnitude of environmental transformation of decaBDE is needed, as the extent of degradation of decaBDE is not fully characterized. This is in agreement with a recent review [23] and with the evidence of transformational processes involving microorganisms or sunlight resulting in more bioavailable and toxic BDEs [21] ,[22]. *Second*, better data on the extent of oral exposure in humans, from food and dust is needed, consistent with. Frederiksen et al., [24]. *Third*, the fate of decaBDE in is not known. The experts questioned whether and to what degree decaBDE is metabolised in the human body to other more accumulating and toxic lower brominated BDEs such as pentadiphenyl ether, or readily excreted with or without metabolism. *In addition*, more knowledge on toxicological health effects was also highly prioritized.

**Table 3:** Priority areas for decaBDE (a) and HBCD (b)

(a)					
***Causal box***	**Source**	**Envir. matrix**	Exposure	Human body	**Social**

***Frequency***	**5**	**7**	12	13	**3**

	***Frequency-item***		***Frequency-group***	***Ranking-item***	***Ranking-total***

Envir.matrix			**7**		
**Transport/transformation**		**4**		**1-1-2-2**	**1**
Exposure		**3**	12	**2-3-4**	
**Ingestional**		**4**		**2-3-3-4**	**2**
Human Body			13		
**Toxicokinetics**		**3**		**1-1-2**	**2**

(b)					

***Causal box***	**Source**	**Envir. matrix**	Exposure	Human body	**Social**

***Frequency***	**5**	**4**	11	17	**3**

	***Frequency-item***		***Frequency-group***	***Ranking-item***	***Ranking-total***

Exposure		**4**	11	**2-2-2-4**	**2**
Human body			17		
**Concentration target organ**		**3**		**1-3-3**	**2**
**Toxicology**		**3**		**1-1-2**	**1**

The table shows the frequency of the different causal boxes (elements) of the causal chain diagram in the priority listings according to their influence on the extent of the health risk the causal diagram leads to (Upper table). The three highest ranked elements (combination of most frequently mentioned and ranking positions), their frequency (how often the element was chosen by the experts; 5 = five experts chose it) and rank (level of priority of an element chosen by the experts; 1 = highest) and their ranking in total (Lower table) is shown.

#### Priority areas for HBCD

There was a general opinion that at present there is insufficient data available to allow health impact assessment of HBCD. The top issues (Table 3b) were largerly in agreement with the recent review [9]. *First*, epidemiological and toxicological studies in humans are needed as the data from toxicological studies of the targets of HBCD and of the mechanism of action of HBCD are limited. This is confirmed by a recent review [9] showing that all results are from animal studies. One recent human epidemiological study on the effects of prenatal exposure of healthy infants to HBCD [36] shows some negative effects on sexual and psychomotor development. *Second*, measurements of HBCD concentrations in the target tissues are needed, due to lack of adequate studies and because of differences in the toxicokinetics between the HBCD isomers. *Third*, studies of human exposure to HBCD was prioritized, since the experts considered that too little is known about normal exposure routes to the general population and that knowledge on exposure is essential in a risk assessment. This is in agreement with the few available studies , such as the indications of transfer of HBCD to cord blood and mother’s milk in humans [40-43] and the estimations of exposure of different population groups [9].

#### Further action for decaBDE and HBCD

Experts disagreed whether today’s knowledge on the risks of decaBDE and HBCD justifies a more drastic policy intervention. Most experts suggested to use the precautionary principle andintroduce regulations restricting and prohibiting activities. A minority of experts felt that more data and better understanding are required before drastic policy measures can be justified.

The arguments for policy actions were:

• Large build-up in the environment was shown for HBCD [44] and for decaBDE [22,28].

• It is unethical to pollute a whole population with a chemical with unknown toxicological properties just to prevent a few deaths caused by fire.

• DecaBDE is persistent and transforms into bioaccumulating and toxic compounds [10,22,23]. The HBCD is persistent and bioaccumulates [9].

• Concentrations of decaBDE were detected in remote areas with no use or production of these compounds, indicating long range transport [22,45], and elevated concentrations were predicted for HBCD [46], confirming that these compounds pose a global problem.

• Comparison with analogous compounds where more knowledge is available, such as polychlorinated biphenyls (PCBs), suggests additional risks. For PCBs, risk was first assessed at high doses in adults, but later more sensitive endpoints were detected at lower doses and in earlier life-stages. One such endpoint could be vitamin K metabolism and subsequent impact on blood coagulation especially in prenatal life, and another endpoint could be leptin metabolism and possibly body weight [47,48].

The arguments against policy actions were:

• The use of suggested alternative compounds [19,20] is not proven to be safer. Less studied compounds not subjected to risk assessment could be introduced.

• Since there is a lack of knowledge regarding the margin of exposure, the human exposure is not big enough to cause toxic effects. The toxicological activity appears to be lower for decaBDE compared to BDEs with lower degrees of bromine substitution [28].

Finally there were suggestions on how to improve knowledge on decaBDE and HBCD:

• Require more research and more toxicological testing from the industry itself.

• Improve the organisation of research cooperation between universities and research institutions at the European level.

• Improve funding for the necessary research.

• Strengthen human cohort studies to include investigations of new medications or chemicals.

• Require permission from an ethical committee for production and use.

• Monitor the levels of decaBDE and HBCD in humans, food and environment to provide insight into the main routes of exposure and to assess the trends.

#### Comments on Methodology

The expert elicitation procedure can be very helpful in quickly obtaining results as a basis for policy consideration, when the scientific uncertainty is high. An advantage of expert elicitation is that different experts may interpret the available data differently, and also the diversity of opinions among the experts is often broader than that reported in consensus documents such as risk assessment reports [5]. All opinions will be heard even if they are raised only by one or a few persons, such as the ethical justification for restriction and banning of the chemicals, as opposed to continued use of the chemicals until there is enough proof of toxic effects. The approach is therefore well suited to identify areas of agreement and disagreement between experts.

The experts in our elicitation argued that the procedure itself could have influenced the results. Individual interpretations or misunderstandings of the questions in the questionnaires may have influenced the responses, and this was considered critical. The experts also missed an optional box of “out of my expertise” or “I don’t know”, instead of only “very low confidence”, because they considered this distinction very important.

Further, experts define differently what constitutes a policy action.This could have influenced the answers in Questionnaire 2.

Most certainly the combination and type of experts have affected the outcome. The loss of some answers in the questionnaires due to technical problems with the web-based solution could also have influenced the final results.

We consider that the different interpretations and misunderstandings were clarified in the workshop, and the final advice provided in the Policy Briefs is robust.

## Conclusions

The HENVINET expert elicitation procedure is an assessment tool aimed at highlighting different view points on knowledge-related key issues for policy making. The procedure is not intended as a substitute for risk assessment, but as its complement. We identified priorities for further research on decaBDE and HBCD, and provided valuable recommendations for policy makers.

Priority issues for further research on decaBDE were: studies of the magnitude of environmental transformation of decaBDE, of the extent of oral exposure in humans, and of the fate of decaBDE in the body. Priority issues for further research on HBCD were: epidemiological and toxicological studies of HBCD, more measurements of the concentration of HBCD in the target tissues, and determination of the extent of human exposure to HBCD.

Based on the answers to the questionnaires and the discussions at the workshop, the invited experts were not in agreement on whether or not the current knowledge is sufficient to justify more strict policy actions at this point. While most experts argued that the persistence of decaBDE and the transformation into bioaccumulating and toxic compounds, and the persistence and bioaccumulation properties of HBCD, are enough to justify a ban or restrictions on use of these compounds, others believed that more data is required before a decision to change the status quo of these economically and technically important compounds is justified.

## Abbreviations

BDE: Brominated diphenyl ether; BDE209: Brominated diphenyl ether 209; decaBDE: Decabromodiphenyl ether; BFRs: Brominated Flame Retardants; BSEF: Brominated Science and Environmental Forum; ECHA: European Chemicals Agency; EHAP: European Health Action Plan; EPA: U.S.Environmental Protection Agency; HBCD: Hexabromocyclododecane; HENVINET: Health and Environment Network; OctaBDE: Octabromodiphenyl ether; PCBs: polychlorinated biphenyls; PentaBDE: Pentabromodiphenyl ether; REACH: Regulatory European Chemicals; Q1: questionnaire 1; Q2: questionnaire 2; VH: Very High; H: High; M: Medium; L: Low; VL: Very Low

## Competing interests

The authors declare that they have no competing interests.

## Author’s contributions

The authors have critically reviewed this manuscript and approved the final version. SR contributed to questionnaire 1, questionnaire 2, the workshop, the policy brief, the evaluation questionnaire, and had the main responsibility of manuscript. KEZ contributed to questionnaire 1, questionnaire 2, the workshop, the policy brief, the evaluation questionnaire and in reviewing the manuscript. HK had a major role in project planning, structuring, and presentation, a main role in data analysis and interpretation of the results, and reviewed all written materials. AG contributed to the early planning phase and all the later phases, in addition to critically reviewing the policy brief and the manuscript. AJM contributed to the early planning phase and in writing the policy brief and the manuscript. JGK contributed to planning the project and also critically reviewing the policy brief and the manuscript. BLM contributed to the later stages of planning and also critically reviewing the policy brief and the manuscript. JLL contributed to all stages of planning the project. GSE contributed to all stages of planning and performing the project, in addition to critically reviewing the policy brief and the manuscript. ER contributed to all stages of planning and performing the project, in addition to critically reviewing the policy brief. JUS contributed to all stages of planning, in addition to critically reviewing the policy brief. MK contributed to designing the casual diagram images used in the web based tools as well as developing the online questionnaire 1 in addition to English proofreading. AY contributed to data analysis and interpretation of the results. AB was the project leader, involved in all stages of the project in addition to approval of all written materials. MKvK was work package 1 leader and heavily involved in planning and performance at all stages of the project, in addition to having the main responsibility for the workshop.

## Workshop participants (in alphabetical order)

Åke Bergman, Stockholm University, Sweden

Lucio G Costa, University of Washington, US

Per Ola Darnerud, National Food Administration, Sweden

Marie Frederiksen, University of Aarhus, Denmark

Helen Håkansson, Karolinska Institutet, Stockholm, Sweden

Janna G Koppe, Ecobaby Foundation, The Netherlands

Jan L Lyche, Norwegian School of Veterinary Science

Cathrine Thomsen, Norwegian Institute of Public Health

Cynthia de Wit, Stockholm University, Sweden

## Supplementary Material

Additional file 1_Q1_decaBDE **Evaluation questionnaire – Causal chain for BFR decaBDE** Questionnaire 1 with explanation and background information. Online version is available at http://henvinet.nilu.no/EvaluationofKnowledge/tabid/1333/language/en-US/Default.aspx Click here for file

Additional file 2_Q1_HBCD **Evaluation questionnaire – Causal chain for BFR HBCD** Questionnaire 1 with explanation and background information. Online version is available at http://henvinet.nilu.no/EvaluationofKnowledge/tabid/1333/language/en-US/Default.aspx Click here for file

Additional file 3_Q2_DecaBDE **Follow-up questions on decaBDE** Questionnaire 2 on decaBDE. Experts were expected to use results of questionnaire 1 (Additional file 3) when answering. Click here for file

Additional file 4_Q2_HBCD **Follow-up questions on HBCD** Description of data: Questionnaire 2 on HBCD. Experts were expected to use results of questionnaire 1 (Additional file 3) when answering. Click here for file

Additional file 5_Policy Brief_decaBDE **HENVINET Policy Brief. Expert Elicitation on Health Implications of decaBDE.** Based on the results from questionnaire 2 and the workshop, a policy recommendation was written as the final product of the project. Click here for file

Additional file 6_Policy Brief_HBCD **HENVINET Policy Brief. Expert Elicitation on Health Implications of HBCD.** Based on the results from questionnaire 2 and the workshop, a policy recommendation was written as the final product of the project. Click here for file

Additional file 7_Q1 Results_decaBDE and HBCD **Additional file 3_Q1 Results** Available data from questionnaire 1 of decaBDE and HBCD. Mean, standard deviation, consensus measure and rank consensus are presented. Click here for file
