# Supplementary material for: Policy relevant Results from an Expert Elicitation on the Human Health Risks of Decabromodiphenyl ether (decaBDE) and Hexabromocyclododecane (HBCD)
Source: Environ Health. 2012 Jun 28;11(Suppl 1):S7. doi: 10.1186/1476-069X-11-S1-S7 (PMC3388476; doi:10.1186/1476-069X-11-S1-S7)
Supplement: Additional file 1_Q1_decaBDE — Evaluation questionnaire – Causal chain for BFR decaBDE Questionnaire 1 with explanation and background information. Online version is available at http://henvinet.nilu.no/EvaluationofKnowledge/tabid/1333/language/en-US/Default.aspx [file 1476-069X-11-S1-S7-S1.pdf]

Evaluation questionnaire – Causal chain for BFR decaBDE

Introduction

Thank you very much for participating in this expert evaluation, conducted in the context of the HENVINET project.

With your help we will evaluate the state-of-the-art in the scientific knowledge of various aspects of the cause-effect relationship between the production and use of deca-brominated diphenyl ether (decaBDE/BDE-209) and its potential impact on health.

The goal is to identify knowledge gaps and potential agreement or disagreement on this between you and your expert colleagues in the field. Ultimately, the aim is to discuss the implications of the results of the evaluation for policy and research.

The evaluation consists of two separate parts. In part A you will be asked to comment on the completeness and structure of the causal diagram illustrating scientists' current understanding of the cause-effect relationship between the production and use of decaBDE and its potential impact on health. In part B you will be asked to express your confidence in scientists' ability to predict various aspects of the health risk assessment of decaBDE/BDE-209.

We expect the entire exercise will take you about 30-45 minutes.

Sometimes experts feel uncomfortable performing evaluations on the basis of their scientific intuition and experience. We ask for your considered opinion based on the quality of your scientific work and rely on the fact that your broad experience in the field will suffice to help achieve a qualified understanding of the issues under discussion here.

Your own expert judgement will be complimented by those of at least 10 of your colleagues in the field, as well as by a thorough review of the literature on this issue. All this information will be considered by a panel of experts during a workshop on the impact of decaBDE on health, at a later time. On this basis, the experts will provide recommendations to policy makers with regards to research and problem solving strategies.

We hope that this will address any concerns you may have. If not, you may contact Gunnar Eriksen: gunnar.eriksen@vetinst.no

We appreciate your participation very much and, on behalf of the Norwegian Veterinary Institute, Norwegian School of Veterinary Science, WHO Euro, Norwegian Institute for Air Research and the HENVINET consortium, we thank you for your time.

Information about yourself

Please tell us about your research background and current institutional affiliation. These data will be confidential.

- Name:____
- Email address: ____
- Institutional affiliation: ____
- 5 keywords describing your area of expertise:

Part A-Evaluation of the diagram

The diagram shown in the figure below illustrates the cause-effect relationship between production and emission of decaBDE and health effects. For a summary explanation of the scientific basis of the diagram, please see Annex 1.

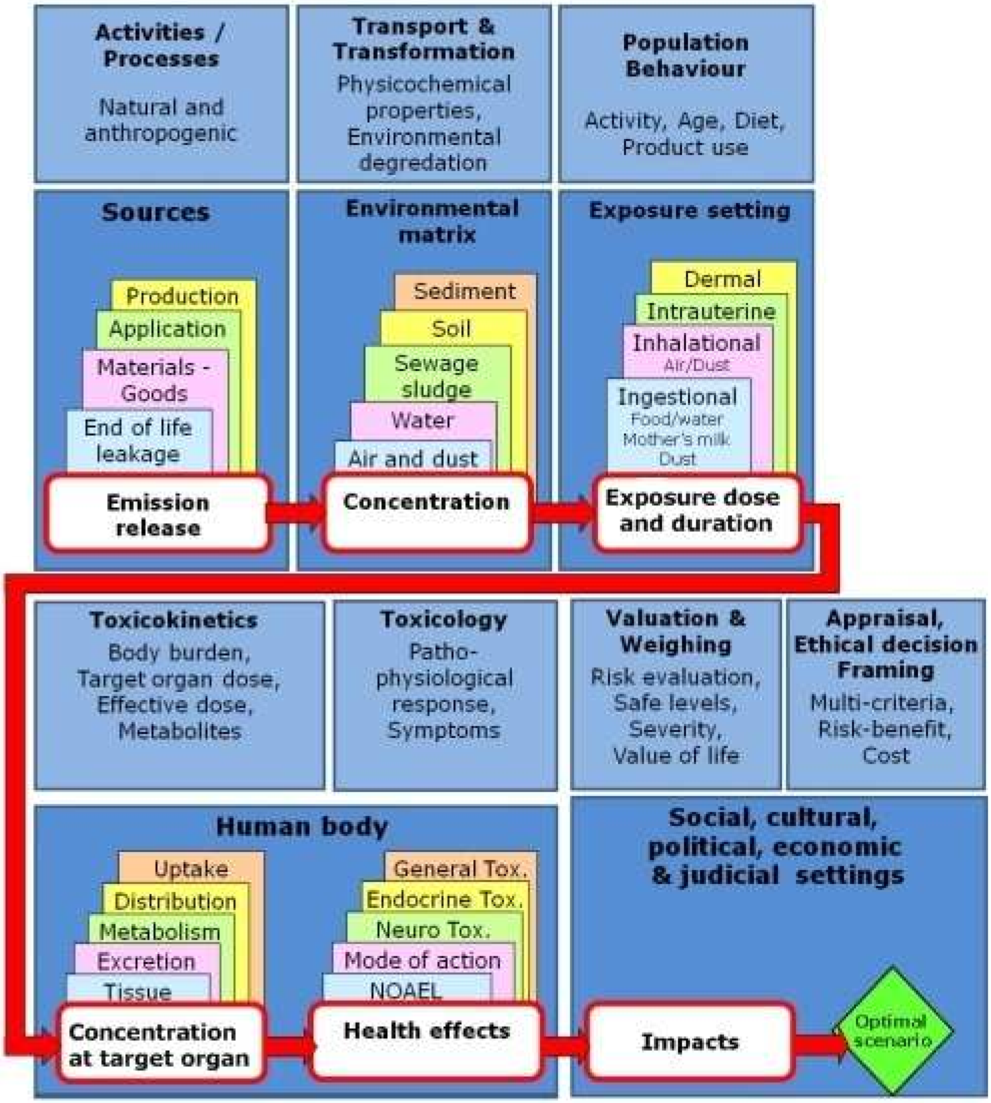

Does the diagram take into account all of the important parameters when evaluating the risks related to production, use and discharge of decaBDE?*

☐ Yes ☐ No

If you said no to the previous question, Please explain:

Are the different causal relationships adequately structured?*

☐ Yes ☐ No

If you said no to the previous question, Please explain:

Are there any unnecessary parameters shown in the diagram that could be deleted?*

☐ Yes ☐ No

If you said yes to the previous question, Please explain:

Part B-Evaluation of individual model parameters

In the questions that follow you will be asked to express your confidence in scientists' ability to predict the concentrations, exposure and effects of decaBDE/BDE-209. Insert a check mark where you feel it is appropriate.

It is important that you consider each question independently of the others. For example, when you answer a question on excretion, do not take into consideration your confidence in the scientists' ability to predict absorption.

Where questions ask for your confidence level, please use these guidelines:

Very high - At least 9 in 10 chance of being correct
High - At least 7 in 10 chance of being correct
Medium - At least 5 in 10 chance of being correct
Low - At least 3 in 10 chance of being correct
Very low - Less than 2 in 10 chance of being correct

Both terms decaBDE and BDE-209 are used in the evaluation form, it is aimed at using decaBDE for the technical mixture and BDE-209 for the single congener.

Sources

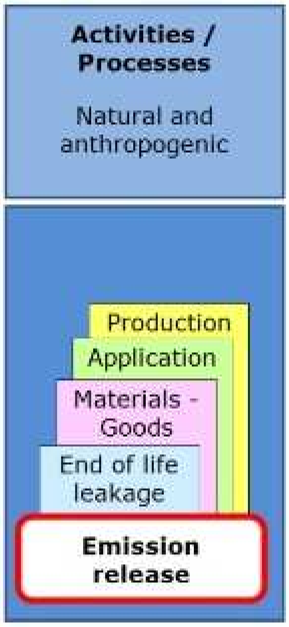

Regarding decaBDE, what is your level of confidence in the quality of the current scientific data on:

Production volumes*

☐ Very high	☐ High	☐ Medium	☐ Low	☐ Very low

Application volumes*

☐ Very high	☐ High	☐ Medium	☐ Low	☐ Very low

Regarding the use of decaBDE in products, what is your level of confidence in the scientists' ability to:

Identify and quantify all different applications*

☐ Very high	☐ High	☐ Medium	☐ Low	☐ Very low

Predict the magnitude of emission/release/leakage during production, use and recycling*

☐ Very high	☐ High	☐ Medium	☐ Low	☐ Very low

Environmental matrix

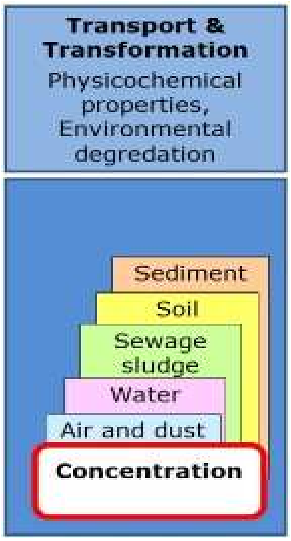

Regarding BDE-209, what is your level of confidence in the scientists' ability to predict:

Environmental transformation, such as debromination, and biological half-lives?*

Very high	High	Medium	Low	Very low

The magnitude of long-range transport?*

Very high	High	Medium	Low	Very low

What is your level of confidence in the scientists' ability to predict the <u>concentration</u> of BDE-209 in:

Sediments?*

Very high	High	Medium	Low	Very low

Sewage sludge?*

Very high	High	Medium	Low	Very low

Soil?*

Very high	High	Medium	Low	Very low

Water?*

Very high	High	Medium	Low	Very low

Dust?*

Very high	High	Medium	Low	Very low

Indoor Air?*

Very high	High	Medium	Low	Very low

Outdoor Air?*

Very high	High	Medium	Low	Very low

Exposure

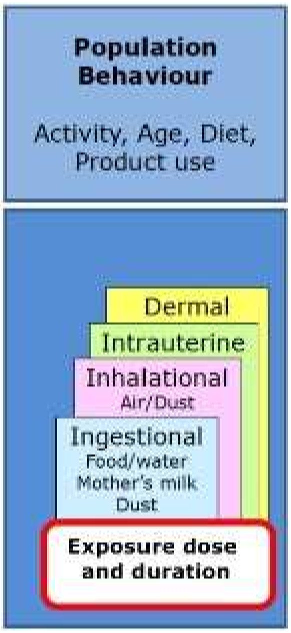

What is your level of confidence in the scientists' ability to predict the <u>level of exposure</u> to BDE-209 in:

The general population?*

☐ Very high	☐ High	☐ Medium	☐ Low	☐ Very low

Occupationally exposed?*

☐ Very high	☐ High	☐ Medium	☐ Low	☐ Very low

Infants and children?*

☐ Very high	☐ High	☐ Medium	☐ Low	☐ Very low

What is your level of confidence in the scientists' ability to predict <u>the main sources of exposure</u> to BDE-209 in:

The general population?*

☐ Very high	☐ High	☐ Medium	☐ Low	☐ Very low

Occupationally exposed?*

☐ Very high	☐ High	☐ Medium	☐ Low	☐ Very low

Infants and children?*

☐ Very high	☐ High	☐ Medium	☐ Low	☐ Very low

What is your level of confidence in the scientists' ability to predict the exposure of <u>the general population</u> to BDE-209 via the following routes:

Direct contact/dermal?*

☐ Very high	☐ High	☐ Medium	☐ Low	☐ Very low

Inhalation?*

☐ Very high	☐ High	☐ Medium	☐ Low	☐ Very low

Ingestion?*

☐ Very high	☐ High	☐ Medium	☐ Low	☐ Very low

What is your level of confidence in the scientists' ability to predict the exposure of <u>occupationally exposed</u> groups to BDE-209 via the following routes:

Direct contact/dermal?*

☐ Very high	☐ High	☐ Medium	☐ Low	☐ Very low

Inhalation?*

☐ Very high	☐ High	☐ Medium	☐ Low	☐ Very low

Ingestion?*

☐ Very high	☐ High	☐ Medium	☐ Low	☐ Very low

What is your level of confidence in the scientists' ability to predict the exposure of infants and children to BDE-209 via the following routes:

Direct contact/dermal?*

Very high	High	Medium	Low	Very low

Inhalation?*

Very high	High	Medium	Low	Very low

Intrauterine?*

Very high	High	Medium	Low	Very low

Via food?*

Very high	High	Medium	Low	Very low

Via breast milk?*

Very high	High	Medium	Low	Very low

Toxicokinetics

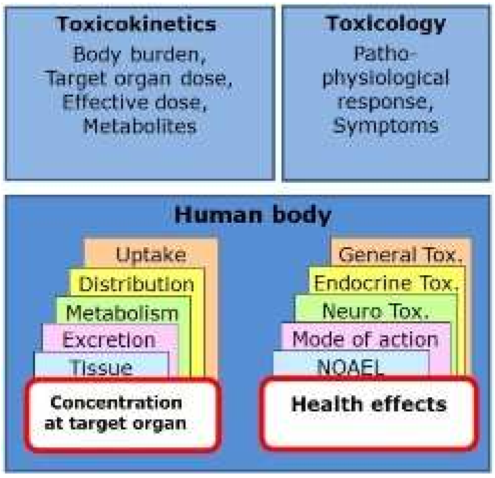

What is your level of confidence in the scientists' ability to predict to what extent BDE-209 is:

Absorbed/taken up?*

☐ Very high	☐ High	☐ Medium	☐ Low	☐ Very low

Metabolised to hydroxy-metabolites after absorption?*

☐ Very high	☐ High	☐ Medium	☐ Low	☐ Very low

Debrominated to lower brominated congeners after absorption?*

☐ Very high	☐ High	☐ Medium	☐ Low	☐ Very low

Debrominated or metabolised by the intestinal microflora?*

☐ Very high	☐ High	☐ Medium	☐ Low	☐ Very low

Accumulating in the body?*

☐ Very high	☐ High	☐ Medium	☐ Low	☐ Very low

Excreted via bile and faeces?*

☐ Very high	☐ High	☐ Medium	☐ Low	☐ Very low

Excreted via urine?*

☐ Very high	☐ High	☐ Medium	☐ Low	☐ Very low

Regarding BDE-209, what is your level of confidence in the scientists' ability to predict

The distribution to different tissues?*

Very high	High	Medium	Low	Very low

The final concentration of <u>the parent compound</u> in the target tissues, taking factors such as absorption, distribution, metabolism and excretion into account?*

Very high	High	Medium	Low	Very low

The final concentration of <u>metabolites</u> in the target tissues, taking factors such as absorption, distribution, metabolism and excretion into account?*

Very high	High	Medium	Low	Very low

The biological half-life?*

Very high	High	Medium	Low	Very low

Toxicology

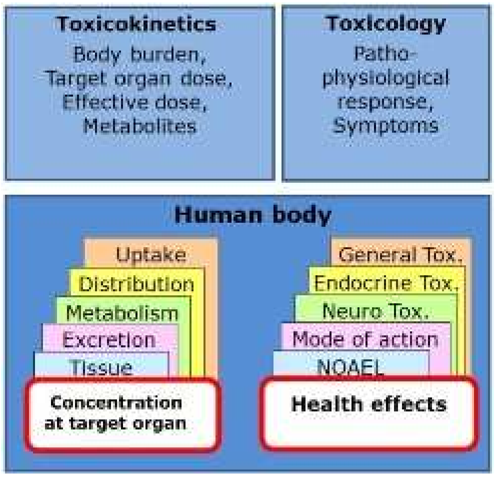

Based on <u>human epidemiological studies</u>, what is your level of confidence in the scientists' ability to predict adverse effects of BDE-209 in

Males?*

Very high	High	Medium	Low	Very low

Females?*

Very high	High	Medium	Low	Very low

Based on experimental animal studies, what is your level of confidence in the scientists' ability to predict adverse effects of BDE-209 on <u>general health</u> in

Males?*

Very high	High	Medium	Low	Very low

Females?*

Very high	High	Medium	Low	Very low

Based on experimental animal studies, what is your level of confidence in the scientists' ability to predict adverse effects of BDE-209 on <u>neurodevelopment</u> in

Males exposed during foetal or neonatal life?*

Very high	High	Medium	Low	Very low

Females exposed during foetal or neonatal life?*

Very high	High	Medium	Low	Very low

Based on experimental animal studies, what is your level of confidence in the scientists' ability to predict adverse effects of BDE-209 on thyroid function in

Males exposed as adults?*

Very high	High	Medium	Low	Very low

Females exposed as adults?*

Very high	High	Medium	Low	Very low

Males exposed during foetal or neonatal life?*

Very high	High	Medium	Low	Very low

Females exposed during foetal or neonatal life?*

Very high	High	Medium	Low	Very low

Based on experimental animal studies, what is your level of confidence in the scientists' ability to predict adverse effects of BDE-209 on reproductive function in

Males exposed as adults?*

Very high	High	Medium	Low	Very low

Females exposed as adults?*

Very high	High	Medium	Low	Very low

Males exposed during foetal or neonatal life?*

Very high	High	Medium	Low	Very low

Females exposed during foetal or neonatal life?*

Very high	High	Medium	Low	Very low

Based on experimental studies, what is your level of confidence in the scientists' knowledge of the mechanisms of action of

BDE-209?*

☐ Very high	☐ High	☐ Medium	☐ Low	☐ Very low

Metabolites of BDE-209?*

☐ Very high	☐ High	☐ Medium	☐ Low	☐ Very low

What is your level of confidence in the scientists' ability to predict the NOAEL of BDE-209?*

☐ Very high	☐ High	☐ Medium	☐ Low	☐ Very low

Final comment

Finally, do you think that any relevant questions were left out or that any questions were superfluous?
Please describe:

All evaluators will be acknowledged in the article that will be based on this questionnaire.

☐ Please check this box if you wish to remain anonymous.

Thank you for taking part in this evaluation and contributing your time and expertise. Your assistance is appreciated.

These results will be analysed and reviewed by experts within the HENVINET consortium.

ANNEX 1 DecaBDE background information

This document is mainly based on recent reviews and reports. Where appropriate, the referred original study is underlined, followed by the review or report in separate brackets. Original studies referred to directly are not underlined.

Both terms decaBDE and BDE-209 occur in the document, it is aimed at using decaBDE for the technical mixture and BDE-209 for the single congener.

Sources

Production and applications

Bromine has flame retarding properties and polybrominated diphenyl ethers have therefore been used for decades in various products to slow down development of fire and thereby save lives and reduce material damage (Frederiksen et al. 2008).
While penta- and octaBDEs were banned in EU in 2004 and followed by 10 states of the USA, decaBDE is still produced and used worldwide (56 100 tons/year in 2001)(Frederiksen et al. 2008). Originally, decaBDE was banned for the use in electrical and electronic applications in the EU together with the other BDEs, but was later exempted from the ban by the Commission. In 2008, the European Court of Justice decided that the Commission had exempted decaBDE on false premises and consequently it was again put a ban to its use in these products (Court of Justice of the European Communities 2008) .
Commercial decaBDE mixtures contain 97% or more BDE-209. The last percentages consist of nonaBDEs and maybe trace amounts of octaBDE (U.S.Environmental Protection Agency 2008).

Materials and goods

Brominated flame retardants have been used in electronics like TVs, computers, mobile phones and in various electrical kitchen appliances. Also upholstery, textiles, building materials and plastic products, as well as cars and airplanes will often contain brominated flame retardants (Frederiksen et al. 2008). DecaBDE is mainly used in textiles and television and computer castings (Costa and Giordano 2007). Approximately ¾ are used in plastics and ¼ in textiles (European Chemicals Bureau et al. 2007).

End of life leakage

Large quantities of brominated flame retardants were found in close proximity to an e-waste recycling site in China, and BDE-209 was the dominating congener in soil and many of the sediment samples. This indicates that electronic devices may contribute to high local environmental levels of BDEs and BDE-209 in particular if not handled appropriately at recycling. A steadily increasing turn-over rate of electronic equipment will be a major contributor to future environmental concentrations (Wong et al. 2007).

Environmental Matrix

BDE-209 makes up a great part of the total PBDE content in air, water and sediments. Relatively recent studies show debromination of deca-BDEs into more toxic and bioaccumulative congeners in abiotic and biotic processes (Ross et al. 2008), such as photolytic debromination in house dust (Stapleton and Dodder 2008) and in plastics (Kajiwara et al. 2008) by natural sunlight. BDE-209 measured on atmospheric particles collected in remote areas as well as in urban regions, suggests that also this congener are transported long distances through the air (de Wit et al. 2006) (Ross et al. 2008). This is also evidenced by its presence in arctic food webs and its relatively high contribution to the total PBDE concentration in some arctic animals (Jenssen et al. 2007).

Sediment

Sediment cores show an historical build-up of the congener. This makes a great reservoir of BDE-209 in sediments which may pose a risk to lower trophic levels while upper trophic levels are more prone to be affected by their potential breakdown products (Ross et al. 2008). BDE-209 accounts for around 80% of the total PBDE in Strait of Georgia sediments (Ross et al. 2008). In European sea water sediments three studies of 10-13 samples have revealed BDE-209 concentrations of 0.03µg/kg dry weight (German Bight 2002-2005) to 132µg/kg dry weight (Spanish coast) (Law et al. 2008). In Belgium, BDE-209 content in the layers of two sediment cores was determined to be in the range of 315 to 8410µg/kg dry weight (Covaci et al. 2005) (Law et al. 2008). Also in European river sediments, BDE-209 constitutes the greatest part of the total BDEs and accounts for approximately 50-60% (Law et al. 2008). So it does in Asian coastal and river sediments, where the highest concentrations are found in Chinese Pearl River (up to 3580µg/kg dry weight) (Mai et al 2005), while 2248µg/kg dry weight was the upper range of what was measured in Korean costal surface sediments (Moon et al. 2007). Besides, levels of BDE-209 still increase in sediments of Tokyo Bay while the other BDEs show a decreasing trend (Minh et al. 2007) (Law et al. 2008).

Sewage sludge

BDE-209 seems to dominate the congener profile in sewage sludge. In altogether 5 studies from 4 European countries sewage sludge samples from 8 to 50 different plants showed

concentrations of BDE-209 ranging from approximately 10µg/kg dry weight (Czech Republic) to 4150µg/kg dry weight (Spain) (Law et al. 2008).
BDE-209 has shown to have a half-life of 700 days under anaerobic conditions in sewage sludge (Gerecke et al. 2006). One study showed no evidence of debromination during the process in wastewater plants (Knoth et al. 2007) (Law et al. 2008).

Soil

BDE-209 was the dominating congener in all soil samples collected from 5 sites in Sweden in 2000, with concentrations of 0.015µg/kg dry weight (reference site) to 3900µg/kg dry weight (sewage sludge amended site). Time since sludge amending in relation to the concentrations measured indicated high persistence of this congener in soil/sludge. No photolytic debromination was seen (Sellström et al. 2005) (Law et al. 2008). The same concentrations were observed at the reference site in a Spanish study (14.6ng/g dry weight), but lower on the amended sites (up to 1082 ng/g dry weight) (Eljarrat et al. 2008).

Water

Discharge of BDE-209 to aquatic systems and coastal oceans is exponentially increasing (Ross et al. 2008). Few studies have measured concentrations of BDEs in water because their hydrophobicity will make them absorb to particulate matter. However, in a study where dissolved and suspended phases of sea-surface micro layer and sea water in Hong Kong were measured, BDE-209 was not measured in concentrations above detection limit in any samples, while BDE-28, BDE-47 and BDE-100 were the dominating congeners (Wurl et al. 2006) (Law et al. 2008).

Dust

BDE-209 is the most abundant of the measured PBDEs in house dust (32-97%). The levels of BDE-209 is higher in North America (630-2000ng/g dw, 6 studies, n between 5 and 64) than in Europe (60-466ng/g dw in continental Europe and Scandinavia 5 studies of n from 1 to 10, 7100 ng/g dw measured in UK, n=10, one study) (Frederiksen et al. 2008). Other measurements from the UK have also revealed high concentrations of the BDE-209 (two samples 520 000 and 100 000 µg/kg, median 2800 µg/kg (Harrad et al. 2007) (Law et al. 2008). In Kuwait, levels of PBDEs in dust were lower than in Europe (83ng/g dw, n=17), but BDE-209 accounted for nearly 90 %, while Singapore levels from dust in air conditioners levelled the North American levels (2200 ng/g dust weight, n=31) (Frederiksen et al. 2008).

Air

The type of sample collector may influence the result. On one hand passive samplers primarily sample the gas phase and therefore may underestimate concentrations of the higher molecular weight BDEs, while on the other hand some active samplers which sample

the particulate phase will underestimate small molecular weight BDEs. Methods of dust sampling also vary (Law et al. 2008). Unlike other congeners, BDE-209 is not evenly distributed between gas and particulate phase; it is present almost exclusively in the particle phase (Venier and Hites 2008).

Indoor air

Homes
BDE-209 is suggested as the main congener in indoor air (median 64-173pg/m^3) in five studies conducted in Europe and North America with between 4 and 73 samples collected in each study (Frederiksen et al. 2008). BDE-209 was measured and found to be the dominant PBDE congener in car-air (Greece:104pg/m^3) (Mandalakis et al. 2008) and concentration of PBDEs were higher in newer cars (Frederiksen et al. 2008).

Occupational
High levels of BDE-209 are found in electronic dismantler halls in Sweden (median 15 340pg/m^3 in air incl particles, n=4 and 30 000 pg/m^3 in "personal air", n=11). BDE-209 was dominating the overall PBDE profile (Frederiksen et al. 2008).

Outdoor air

Levels are lower in outdoor air, but BDE-209 is still among the important congeners (Frederiksen et al. 2008; Law et al. 2008). Atmospheric concentration was measured near Lake Maggiore in Northern Italy in March 2005 using a high volume sampler. BDE-209 was only present in particulate phase with a concentration of 4.79 pg/m^3 and it was the third most abundant congener measured (Mariani et al. 2008). In Turkey, atmospheric BDE-209 concentrations were measured at one suburban, two urban and one industrial site. BDE-209 dominated the total PBDE profile at all sites. A modified high volume-sampler was used to sample the 60 samples at each of the sites during summer and winter months. Total concentration (gas and particulate phase) of BDE-209 was in the range of 19 (suburban) to 54 (industrial) pg/m^3 in summer and in the range of 10.9 (suburban) to 32.5 (industrial) pg/m^3 in winter. On average the proportion of BDE-209 was 70 +/- 22% at the four sites (Cetin and Odabasi 2008).

Exposure

Dermal

Dermal absorption might happen by direct contact with textiles, furniture, electrical equipment or house dust. Some studies regard this as an important contributor to the overall exposure (Frederiksen et al. 2008). Dermal contact with indoor house dust was

calculated to account for 16% of the total body burden of PBDEs in adults (Lorber 2008) (Frederiksen et al. 2008).

Inhalation

Particles or compounds in the gaseous phase or particles will be inhaled and ingested via mucus (Frederiksen et al. 2008). This is probably an important source, however, little is known about how much this contributes to the overall exposure. BDE-209 is the most abundant congener in air, and constitutes up to 62% of total amount of PBDE (Karlsson et al. 2007) (Frederiksen et al. 2008).

Intrauterine

Little is known to which extent the foetus is exposed *in utero*. In a recent French study, BDE-209 was found only in 50% of the cord serum samples, while it was found in 90% of the maternal serum samples. Measured levels however, were much higher in cord serum (median 27.11, range 3.43-363.33ng/g lw, n=36) compared to the maternal serum (median 5.78, range 0.79-37.43ng/g lw, n=64). The reason for this was suggested to be the lower lipid content in cord serum. The median relative contribution of decaBDE in cord serum was 77% to the total levels of octa-, nona- and deca-BDE (Antignac et al. 2009). A Spanish study measured BDE-209 concentrations in cord serum to be 1.4-2.2ng/g lw (median at two different hospitals in Madrid, n=53 and 44, respectively), while it was the predominant congener in placenta (1.0ng/g lw, n= 30) (Gomara et al. 2007). High levels measured in toddlers might be due to breast milk consumption and higher exposure to dust than adults (U.S.Environmental Protection Agency 2008).

Ingestional

Mother's milk

Levels of BDE-209 have shown to be constant from 1987 until 1999 in samples from Faeroe Islands (Frederiksen et al. 2008)
BDE-209 was detected in low concentrations in all samples of mother's milk sampled during 2000-2002 from 10 primipara mothers living in Northern Norway (Polder et al. 2008b). In a French study published in 2009, BDE-209 was found in all 62 analysed breast milk samples from 20 to 46 years old women, mean 32.5, (range 0.39-6.80ng/g lw, median 1.62ng/g lw) and it was the dominating of the higher brominated congeners (relative contribution of 45% to total octa- to decaBDE concentration) (Antignac et al. 2009). In breast milk from two different areas of Madrid, Spain, sampled three weeks after delivery, BDE-209 was the most abundant congener (2.9ng/g lw, n=22 and 2.8ng/g lw, n=30) (Gomara et al. 2007). Mother's milk from North America has shown mean concentrations of 0.8 (first time mothers, after 8 weeks nursing) and 0.9ng/g lw (20-41 years old) in two different studies with n of 40 and 47, respectively (She et al. 2007, Schecter et al. 2003) (U.S.Environmental Protection Agency

2008). Samples collected from 19 primiparous mothers living in an urban or a rural area of Eastern China contained higher concentrations of BDE-209 than of the other congeners (median: 2.6ng/g lw, all samples), but was only detected in 50% of the samples (Sudaryanto et al. 2008).

Food

Seafood is an important source of BDE-209, especially fatty fish and fish livers. However, meat, eggs and dairy products seem to be a relatively more important source of BDE-209 than of the other PBDEs, probably because of the short half-life of BDE-209; terrestrial animals live closer to the sources than fish do (Ohta et al. 2002) (Frederiksen et al. 2008). Levels of BDE-209 in cod liver and herring from Danish waters have been as high as 50000-60000 pg/g ww, while the general trend is that BDE-209 accounts for less than 10% of the total PBDE content in fish. Much larger fractions are measured in shellfish from Korea and the Netherlands (Frederiksen et al. 2008).
Strong correlation was seen between consumption of fish from a contaminated lake and serum levels of PBDEs. This was not the case for BDE-209, suggesting that other sources than dietary fish are important (Thomsen et al. 2008). Another study indicated that milk products could contribute considerably to the BDE-209 intake (Knutsen et al. 2008). Although levels of PBDEs in vegetables are generally low, spinach has shown to contain large amounts, though BDE-209 was not measured in that study (Frederiksen et al. 2008).

Egg content of BDE-209: 10pg g/ww, n=1, 2003/04, USA
Meat: 38 pg g/ww, n=18, 2003/04, USA
Meat: below detection limit, n= 4-26, 2005, Belgium
Chicken breast: 48pg/g ww, n=1, 2003/04, USA
Cheese: below detection limit, n=3, 2005, Belgium
Milk products: 9.1pg/g ww, n=15, 2003/04, USA
Dairy products: 4.42pg/g ww, n=18, 2003-05, Spain
Oils: 24pg/g ww , n=16, 2003-05, Spain
Infant formula: 14pg/g ww, n=1, 2003/04, USA
(Various authors)(Frederiksen et al. 2008)

Dust

Dust is an important source of BDE-209. Dust in gaseous and particulate phase is inhaled and ingested with mucus (Frederiksen et al. 2008). Dust is regarded the most important source of BDE-209 exposure for many people in the UK and can be inhaled and then ingested as well as absorbed through skin via direct contact, while penta-BDE mixtures mainly are ingested via food (Law et al. 2008). BDE-209 is the most abundant of the measured PBDEs in house dust (32-97%). The levels of BDE-209 is higher in North America (630-2000ng/g dw, 6 studies, n between 5 and 64) than in Europe (60-466ng/g dw in continental Europe and Scandinavia 5 studies of n from 1 to 10, 7100 ng/g dw measured in UK, n=10, one study) (Frederiksen et al. 2008). Other measurements from the UK have also revealed high

concentrations of the BDE-209 (two samples 520 000 and 100 000 µg/kg, median 2800 µg/kg (Harrad et al. 2007) (Law et al. 2008). In Kuwait, levels of PBDEs in dust were lower than in Europe (83ng/g dw, n=17), but BDE-209 accounted for nearly 90 %, while Singapore levels from dust in air conditioners levelled the North American levels (2200 ng/g dust weight, n=31) (Frederiksen et al. 2008).

Human body

Toxicokinetics

Uptake

There are no direct quantitative studies on BDE-209 absorption in humans; however, measured concentrations in humans indicate absorption (U.S.Environmental Protection Agency 2008). For example, uptake from air/air particulates may happen through inhalation of particulates followed by swallowing or direct dermal contact and is indicated by clear evidence of occupational exposure. It is shown to be taken up in animals of the aquatic food web, but at lower levels than other congeners. Its uptake is probably hindered by particle binding (Ross et al. 2008). However, BDE-209 is demonstrated to bioaccumulate in terrestrial food chains and mammal predators, and it may be more important for birds feeding in terrestrial, than in marine habitats (Law et al. 2008). Though also in mammals (rodents) absorption of decaBDE is much lower than for lower brominated congeners (Morck et al. 2003)(Costa and Giordano 2007). Absorption range of 7-26% is indicated for rats; however, accurate measurements are difficult because of the high content of the compound and metabolites in faeces. It is indicated that absorbed and metabolised decaBDE accounted for around 10% of the faecal excretion (U.S.Environmental Protection Agency 2008). Very small amount has been shown to be absorbed through mice skin in vitro. Only 0.07-0.34% had passed through the skin sections 24 hrs after they were exposed to 6, 30, and 60nmol in a flow through diffusion system and the percentages passed were inversely related to dose (Hughes et al.2001) (U.S.Environmental Protection Agency 2008).

Distribution

BDE-209 distributes differently from the other highly brominated congeners, which are found in the highest concentrations in adipose tissue (Morck et al 2003) (Costa and Giordano 2007). Hydrophilic metabolites, molecular mass and favoured conformation may be factors leading to a low uptake by adipocytes (U.S.Environmental Protection Agency 2008). Most available knowledge on distribution in humans originates from monitoring of levels in human populations. Thus, data is scarce and mostly limited to milk and blood. BDE-209 is distributed and secreted in human milk but is found at low levels compared to other congeners (U.S.Environmental Protection Agency 2008; Polder et al. 2008a). In blood however, BDE-209 seems to reach higher levels, and levels in an infant and a toddler showed to be unusually high compared to their parents (U.S.Environmental Protection Agency 2008). In French mothers, levels of BDE-209 were found to be highest in cord serum (where the compound was found, median 27.11ng/g lw), followed by lower levels in maternal serum

(median 5.78ng/g lw), milk (median 1.62ng/g lw) and adipose tissue (median 0.75ng/g lw) (Antignac et al. 2009).
Metabolites were found in both maternal and foetal tissues after exposure of pregnant rats to BDE-209. Thus the metabolites can pass placenta and enter the foetus (Riu et al. 2008) (Legler 2008). The congener has also shown to dominate the PBDE profile in human placentas (1.0ng/g lw, n=30) in a Spanish study (Gomara et al. 2007). Presence of BDE-209 has been detected in mouse neonatal brain and heart (Viberg et al. 2003) (Legler 2008). The compound was also detected in all tissues examined in rats 3-7 days after oral exposure to 2.9mg/kg 14C-labelled and unlabelled decaBDE: liver, adipose tissue, lung, kidney, adrenal glands, skin, muscle, spleen, testis, thymus, heart, plasma and colon wall and small intestine wall. Levels measured indicated distribution to blood rich tissues rather than to lipid-rich tissues (Morck et al. 2003) (U.S.Environmental Protection Agency 2008). In more studies, highest concentrations were measured in liver and plasma, though overall the relative distribution to different tissues varies across studies in adult rodents. Age-dependent differences in distribution to liver and developing brain have also been revealed (U.S.Environmental Protection Agency 2008).

Human tissue levels

Blood
In 21 pooled serum samples from the general Norwegian population, BDE-209 were detected in all samples, however, they did not show the same time trends as the other congeners, which rose until late 1990's and then stabilised. This might be due to the shorter half-life of BDE-209 (Thomsen et al. 2007) (Law et al. 2008). In Japan, 89 mothers had a range of 0.74-21.19 ng/g lipid sum of PBDEs in their blood and the most prominent congener was BDE-209 (Inoue et al. 2006) (Costa and Giordano 2007). In 64 analysed serum samples of mothers collected in France, the concentration range was 0.79ng/g lw-37.43ng/ g lw and the median value was 5.78ng/g lw (Antignac et al. 2009).
7 year old children had a higher blood level of BDE-209 than their mothers (Frederiksen et al. 2008). In one Californian family, the breast fed infant serum concentration of BDE-209 reached 233ng/g lw, in the 5 –year old sister 143ng/g lw, while the mother and father had serum concentrations of 14 and 23ng/g lw, respectively (Fischer et al. 2006) (U.S. Environmental Protection Agency, 2008). High concentrations were also measured in occupationally exposed individuals in Sweden (up to 34ng/g lw) and China (86 and 310ng/g lw) (Frederiksen et al. 2008).

Milk
Other PBDEs are dominating in human milk samples, while levels of BDE-209 were found to be low as seen in Northern Norway (median 0.13ng/g lw)(Polder et al. 2008b), North western Russia (median 0.19ng/g lw, n=37) (Polder et al. 2008a), France (median 1.62ng/g lw) (Antignac et al. 2009), in the Pacific Northwest of the U.S. and British Columbia (median 0.4ng/g lw) (She et al. 2007) and in U.S. mothers with various ethnical backgrounds (mean 0.9ng/g lw) (Schecter et al. 2003) (U.S.Environmental Protection Agency, 2008). In Eastern China median concentration was found to be 2.6ng/g lw, but BDE-209 was only detected in 50% of the samples (Sudaryanto et al. 2008).

Adipose Tissue
Adipose tissue levels in samples collected during caesarean sections could be quantified in 79 of the 86 samples collected. Concentration ranged from 0.13 to 4.39ng/g lw and the median value was 0.75ng/g lw. This was lower than in milk, maternal serum and in cord serum which were sampled in the same study (Antignac et al. 2009). In Japan, median concentration of BDE-209 in adipose tissue from 28 donors (18 males and 10 females) collected at autopsy during 2003-04, was 1.2 ng/g lw (range: <0.5-12) in males and 0.74 ng/g lw (range: <0.5-1.7) in females (Kunisue et al. 2007).

Metabolism

Data regarding metabolic processes in humans is difficult to achieve.
BDE-209 is readily metabolised in rodent tissues (U.S.Environmental Protection Agency 2008). While the other PBDE congeners are metabolised to mono- or di-hydroxylated metabolites, BDE-209 is both metabolised to hydroxylated metabolites and debrominated to other congeners, such as the more accumulating and toxic nona-, octa-, and heptaBDEs (Morck et al. 2003; Huwe and Smith, 2007) (Costa and Giordano 2007). Reductive debromination is suggested to be the first step in the metabolic pathway followed by oxidation to phenolic metabolites. Some debromination may happen through the activity of the CYP1A1 and CYP2B1 enzymes (Zhou et al. 2001) (U.S.Environmental Protection Agency 2008). Metabolism may occur both in liver and in epithelium of the gastrointestinal tract or by the intestinal micro flora. After i.v. injection of decaBDE in rats 63% of the fecal BDE-209 content was metabolised, while 37% was intact BDE-209 (el Dareer et al. 1987) (U.S.Environmental Protection Agency 2008). In a 21-days dietary exposure study in rats, hepta- to nonaBDEs constituted 1.5% of the dose of decaBDE mixture, whereas they accounted for 16-22% of the total PBDE concentration measured in the tissues. Some of these lower brominated congeners showed a much higher propensity to bioconcentrate than BDE-209 (Huwe and Smith 2007). Evidences of *in vivo* debromination are also observed in starlings (van den Steen et al. 2007), fish and fish liver microsomes (Stapleton et al. 2006).

Excretion

BDE-209 appears to be excreted more rapidly than the other congeners. The half-life in humans is days to months for octa- to decaBDEs in contrast to the lower brominated congeners which half-lives are in the order of years (Costa and Giordano 2007). Thuresson et al. 2006 estimated the half-life of BDE-209 in humans to be 15 days. Shorter and longer half-lives have been found in rats; after oral and intravenous injection of one single dose of decaBDE using a three-compartment model, the half-life was estimated to be around in total 2.5 days (Sandholm et al. 2003) (U.S.Environmental Protection Agency 2008), while repetitive oral dosing for 21 days led to a half-life of 75 days in rats (Huwe and Smith 2007) (Costa and Giordano 2007). When all metabolites are taken into account, the half-life is expected to be prolonged (U.S.Environmental Protection Agency 2008). Main route of excretion in rodents is through faeces, while urinary excretion seems to be of minor importance (less than 1%). After oral exposure most BDE-209 (around 90%) is excreted unabsorbed in faeces. Biliary excretion accounts for approximately 10% of the amount

measured in faeces. Most (around ¾) of the dose will be excreted within 72 hours regardless the administration method, and most of this during the first 24 hours (U.S.Environmental Protection Agency 2008).

Toxicology

General toxicity

DecaBDE is less potent than the other BDEs. Knowledge on general toxicity is obtained through animal experiments. The observations suggest that males are more sensitive than females (NTP, 1986) (U.S.Environmental Protection Agency 2008)

Short term studies (up to 14 days) in adult rodents did not reveal any effects on the endpoints examined at the applied oral doses of decaBDE of 97-99% purity (up to 20 994mg/kg/day in males and 23 077mg/kg/day in females) (U.S.Environmental Protection Agency 2008).
In oral exposure studies of longer duration (decaBDE with 94-97% purity for two years, starting from 7-8 weeks of age), the following was observed:

- Male rats exposed to 0, 1120 or 2240mg/kg/day: significant increased incidence of liver thrombosis, liver degeneration, fibrosis of the spleen, lymphoid hyperplasia in the mandibular lymph node in the high dose group and increased incidence of neoplastic nodules in the liver in both low- and high-dose groups
- Female rats exposed to 0, 1200 or 2550mg/kg/day: the only significant finding was increased incidence of neoplastic nodules in the liver in the high dose group
- Male mice exposed to 0, 3760 or 7789mg/kg/day: significant increased incidence of centrilobular hypertrophy in the liver and follicular cell hyperplasia in thyroid gland in the high- and low-dose group
- Female mice exposed to 0, 3760 or 7780mg/kg/day: statistically significant increase in the incidence of stomach ulcers in the high dose-group.

Genotoxicity
Despite the observed increased incidences of neoplastic nodules in rats, decaBDE has shown not to be genotoxic. No chromosomal aberrations or sister-chromatid exchanges were observed in Chinese hamster ovary cells exposed to doses of up to 500µg/ml in presence and absence of an exogenous metabolic system. Parent decaBDE in the presence or absence of exogenous metabolic system did not exert mutagenic properties either when tested in vitro on *Salmonella typhimurium* strains (up to 10 000µg/ml) or in a mouse lymphoma cell assay system (up to 10µg/ml) (NTP, 1986) (U.S.Environmental Protection Agency 2008). Increase in reactive oxygen species were found in human hepatoma cells after exposure to 10-100µM BDE-209 (Hu et al. 2007) (Costa and Giordano 2007).

Neurotoxicity

Neurobehavioral effects have been reported after exposure to single and repetitive doses during critical windows of development in rodents. PBDEs are reported to interfere with thyroxin levels and this might contribute to behavioural changes as these hormones play a crucial role in brain development (Costa and Giordano 2007). Also neurodevelopmental toxicity studies indicate that males may be more sensitive to decaBDE than females, although females have not been studied to the same extent as males.

When the animals are exposed during development, much smaller doses over shorter periods, even single doses may cause effects. Time of exposure has been shown to be of great importance for development of adverse neurological effects. A Swedish research group has performed several behavioural studies in rats and mice, where the endpoints rearing, locomotion and total activity in rodents exposed neonatally (decaBDE, >98-99% purity, in 20% fat emulsion, oral gavage) were assessed in three successive 20-minute periods months after exposure. The following findings are published:

- Post natal day (PND) 10 was suggested to be a sensitive window in rat and mouse brain development. However, exposure to decaBDE only caused effects when the animals were exposed prior to this, on PND 3, probably due to the slow accumulation in the brain of this congener or its metabolites. Interestingly, no effects were seen when exposure took place at PND10 or 19, and when exposed on PND 19 also brain accumulation of BDE-209 was lower (Viberg et al. 2003) (Costa and Giordano 2007).
- Male mice treated with 20.1 mg/kg/bw decaBDE on PND 3 showed abnormal habituation; reduced activity for locomotion, rearing and total activity compared to controls the first 20-minute period and hyperactivity the third 20-minute period of the test at 2, 4 and 6 months of age (Viberg et al. 2003) (U.S.Environmental Protection Agency 2008).
- Male mice exposed to 2.22mg/kg decaBDE on PND 3 only showed minor changes in behaviour when subjected to the same tests as the 20.1mg/kg exposed mice (Viberg et al. 2003) (U.S.Environmental Protection Agency 2008).
- Male rats exposed to 20.1mg/kg decaBDE on PND 3 showed similar pattern in behaviour as the 20.1mg/kg treated mice at 2 months of age. The test was not carried out at 4 or 6 months (Viberg et al. 2007) (U.S.Environmental Protection Agency 2008)
- Male rats exposed to 6.7mg/kg decaBDE on PND 3 showed increased locomotion and decreased rearing during the second 20-minute period and increased total activity during the first and second 20 minute periods at 2 months of age (Viberg et al. 2007) (U.S.Environmental Protection Agency 2008).
- Decreased activity in 20.1mg/kg exposed rats treated with nicotine compared to 20.1mg/kg exposed rats injected with saline, suggested that BDE-209 might interfere with the cholinergic system (Viberg et al 2007) (U.S.Environmental Protection Agency 2008)

- Male mice exposed to doses of 1.34, 2.22, 13.4 and 20.1 mg decaBDE /kg bodyweight on PND 3 displayed a dose-related change in all three test variables; locomotion, rearing and total activity at two and four months of age. The test was not carried out at 6 months of age (Johansson et al. 2008).
- Male mice given 2.22 to 20.1mg/kg/ bw decaBDE were less active than controls and mice in the lowest exposure group during first 20 minutes at 2 and 4 months of age (Johansson et al. 2008).
- 13.4 and 20.1 mg/kg (also 2.22 at four months) exposure groups of male mice were significantly more active during the last 20-minute period at 2 and 4 months of age (Johansson et al. 2008).
- Also in this study, the nicotine- injected animals of the two higher dose-groups showed less activity than the saline injected during the 20 first minutes, in contrast to the control group and lower dose groups, and higher activity than controls during the last 20 test minutes (Johansson et al. 2008).

Another research group has also studied effects on neurodevelopment in mice:

- In mice of both sexes exposed orally to 6 and 20mg/kg decaBDE (99.5% purity in 20% fat emulsion, administered by a micropipette) during post natal days 2-15, it was observed a delay in palpebral reflex on PND 14 and the 6mg/kg-group struggled more during handling than controls on PND 20 (Rice et al. 2007) (Costa and Giordano 2007).
- Male 20mg/kg/ day group did not perform an effective forelimb grip on PNDs 14 and 16 (Rice et al. 2007) (Costa and Giordano 2007).
- Locomotor activity in a new environment declined over a 2 hrs period in all animals in the same study on PND 70, however, both exposure groups of males showed less decline than control, while females showed hypoactivity compared to controls. These effects were no longer seen when the mice were one year old (Rice et al. 2007) (Costa and Giordano 2007).

Experiments with killifish, also showed hypo-and hyperactivity depending on dose, when the fish were exposed during embryonic stage (Timme-Laragy et al. 2006) (Costa and Giordano 2007).

Endocrine toxicity

Observations from studies on endocrine system include interference of decaBDE with the thyroid hormones, some effects on male reproduction and altered expression of genes important for hormonal homeostasis. Knowledge on endocrine toxicity is obtained from animal experiments.

In female Wistar rats exposed to decaBDE for 28 days increased levels of circulating triiodothyronine were observed after exposure to a high dose (van der Ven et al. 2007) (Legler 2008). Oral exposure of mice to a dose of 6 and 20mg/kg on postnatal day 2-15 decreased levels of plasma T_4 in males in a dose- related fashion on PND 21 (Rice et al 2007) (U.S. Environmental Protection Agency, 2008).

No expression of an oestrogen receptor-mediated luciferase reporter construct was seen after exposure of zebra fish to different BDEs among them BDE-209, through food and water (Legler et al. 2005) (Legler 2008). Oral exposure of rats to decaBDE (>97% purity, phospholipon emulsion) for 28 days caused increased male accessory reproductive organ sizes at a bench mark dose level of 0.2mg/kg bw/day, as well as increased hepatic expression of CYP1A and CYP2B (BMDL 0.5-0.7mg/kg bw/day) and decreased expression of the steroidogenic enzyme CYP 17 in female adrenals (BMDL 0.18mg/kg bw/day) (van der Ven et al. 2008). Doses of 10-1500mg/kg/day (oral gavage, 98% purity in corn oil) from PND 21-70 did not affect sperm count or function in mice, but there were indications of sperm oxidative stress and a decrease in amplitude of lateral head displacement and decreased number of sperm with high mitochondrial membrane potential in the 500 and 1500mg/kg/day exposed mice (Tseng et al. 2006) (U.S.Environmental Protection Agency 2008).

In 2002, few effects were observed in a developmental toxicity study where mated females were administered decaBDE of 97.34% purity in corn oil by oral gavage during gestation days 0 through 19. Doses applied were 0, 100, 300 and 1000 mg/kg/day and dams were sacrificed at GD 20. A statistically significant increase in food consumption up to day 12 of gestation and in the mean number of early resorptions per dam were observed in the high dose group, but neither of these findings were considered toxicologically significant (Hardy et al. 2002) (U.S.Environmental Protection Agency 2008).

Mode of action

DecaBDE may act via different modes of action. Different endpoints have been studied.

Mice exposed orally to 20.1mg/kg decaBDE on post-natal day 3 showed decreased levels of autophosphorylated-active alpha Ca^{2+}/calmodulin-dependent protein kinase II, brain derived neurotrophic factor and Gap-43 (neuromodulin) which were investigated for being possible contributors to the observed neurodevelopmental effects in the same animals. Also, the rats exposed to 20.1 and 13.4mg/kg were hypoactive when injected with nicotine compared to control animals and those exposed to lower doses. This indicates interference of BDE-209 with the cholinergic system when the animal is exposed during brain development (Viberg et al. 2007) (Costa and Giordano 2007; Johansson et al. 2008).

4 days of 100mg/kg/day exposure of weanling rats did neither reveal change in thyroid hormone levels, nor in activity of hepatic enzymes which could affect thyroxin homeostasis (Zhou et al. 2001) (U.S.Environmental Protection Agency 2008).

There is evidence that BDE-209 may affect steroid hormone homeostasis as it has induced expression of steroid metabolising CYP-enzymes (cyp3a11 and 2b10, but not cyp1a1/2) (Pacyniak et al.2007) (Legler 2008) in mice. In a recent study, hepatic expression of CYP1A mRNA in rats of both sexes and CYP2B mRNA in males and their respective enzyme activities were increased (BMDL 0.5-0.7mg/kg bw/day) and expression of the steroidogenic enzyme CYP 17 in female adrenals decreased (BMDL 0.18mg/kg bw/day) (van der Ven et al. 2008). DecaBDE have earlier shown not to activate Ah receptor, but have appeared to be a very

weak oestrogen receptor antagonist, which is most likely of no biological significance (U.S.Environmental Protection Agency 2008). It has also shown to activate receptors (PXR, dose dependent up-regulation, doses 0.1-100 µM and SXR, only at 100 µM) related to expression of the induced CYP enzymes in vitro (Pacyniak et al. 2007)(Legler 2008).

NOAEL

EPA 2008 report on decaBDE uses the NOAEL from Viberg et al. (2003), 2.22mg/kg as a point of departure for estimating the oral reference dose (RfD). The LOAEL in this study, 20.1mg/kg administered orally on PND 3 to mice gave effects on locomotion, rearing and total activity at 2, 4 and 6 months of age (U.S.Environmental Protection Agency 2008). Two other studies also revealed the neurobehavioral effects to the same dose and lower doses (around 6mg/kg) in rats and mice dosed at the same PND (Viberg et al. 2007) or during the same period of life (PND2-15) (Rice et al. 2007). These two studies did not identify a NOAEL as effects were seen in the lowest dose groups. None of the three studies included more than two exposure groups. The study design in the Viberg et al. (2003) study has been criticized. 10 mice were randomly selected from three to five litters in each treatment group. EPA discusses this as a potential introduction of litter effect and biased results. EPA also mentions that more neurobehavioral endpoints could have been included and that only males were studied. The effects revealed by the Rice et al. (2007) study support and strengthen the evidences, as other neurobehavioral endpoints, and mice of both sexes were included. EPA states that the effects seen only after a single dose given in the Viberg et al. (2003) study increases the concern of the observed effects. This study also suggests PND 3 to be a critical window in brain development. These factors in addition to the supporting evidence from other studies were important in EPA's decision to use this as a point of departure for calculating the oral reference dose. The RfD was calculated to be 7µg/kg/day, when the following uncertainty factors were used: extrapolating animal data to humans (10), susceptible human subpopulation (10) and extrapolating from a single-dose to a life-time exposure (3) (U.S.Environmental Protection Agency 2008).

A new experiment conducted in 2008 by the Viberg group suggests effects at lower doses for the same neurobehavioral endpoints (Johansson et al. 2008).

Based on two studies in rodents, EPA has estimated the limit for the effective dose (LED12) to be 178 mg/kg/day for the most sensitive endpoints (neoplastic nodule or carcinoma) and used this as the point of departure for calculating cancer oral slope factor; $7*10^{-4}$ per mg/kg/day. The doses associated with excess cancer risk of 10^{-4}, 10^{-5} and 10^{-6} are approximately 100, 10 and 1 µg/kg/day. The estimate is somewhat uncertain as some neoplastic nodules today would be characterized as non-neoplastic hyperplasia and the slope factor assumes that all neoplastic nodules were preneoplastic cellular changes with the potential to become malignant (U.S.Environmental Protection Agency 2008).

Reference List

Antignac, J. P., R. Cariou, D. Zalko, A. Berrebi, J. P. Cravedi, D. Maume, P. Marchand, F. Monteau, A. Riu, F. Andre, and Bizec B. Le. 2009. Exposure assessment of French women and their newborn to brominated flame retardants: determination of tri- to deca- polybromodiphenylethers (PBDE) in maternal adipose tissue, serum, breast milk and cord serum. Environ.Pollut. 157.164-73.

Cetin, B., and M. Odabasi. 2008. Atmospheric concentrations and phase partitioning of polybrominated diphenyl ethers (PBDEs) in Izmir, Turkey. Chemosphere 71.1067-78.

Costa, L. G., and G. Giordano. 2007. Developmental neurotoxicity of polybrominated diphenyl ether (PBDE) flame retardants. Neurotoxicology 28.1047-67.

Court of Justice of the European Communities. Judgement of the Court (Grand Chamber) (Directive 2002/95/EC- Electrical and electronic equipment - Restriction of use of certain hazardous substances - Decabromodiphenyl ether ('DecaBDE') - Commission Decision 2005/717/EC - Exemption of DecaBDE from the prohibition on use - Actions for annulment - Commission's implementing powers - Infringement of the enabling provision). 1-4-2008.
Ref Type: Report

Eljarrat, E., G. Marsh, A. Labandeira, and D. Barcelo. 2008. Effect of sewage sludges contaminated with polybrominated diphenylethers on agricultural soils. Chemosphere 71.1079-86.

European Chemicals Bureau, Sazan Pakalin, Thomas Cole, Johann Steinkeller, Nicolas Ronan, Chrystele Tissier, Sharon Munn, and Steven Eisenreich. Review on production processes of decabromodiphenyl ether (decabde) used in polymeric applications in electrical and electronic equipment and assessment of the availability of potential alternatives to decabde. EUR 22693 EN. 2007. European Chemicals Bureau, Institute of Health and Consumer Protection, Joint Research Centre, European Commission.
Ref Type: Report

Frederiksen, M., K. Vorkamp, M. Thomsen, and L. E. Knudsen. 2008. Human internal and external exposure to PBDEs - A review of levels and sources. Int.J.Hyg.Environ.Health.

Gomara, B., L. Herrero, J. J. Ramos, J. R. Mateo, M. A. Fernandez, J. F. Garcia, and M. J. Gonzalez. 2007. Distribution of polybrominated diphenyl ethers in human umbilical cord serum, paternal serum, maternal serum, placentas, and breast milk from Madrid population, Spain. Environ.Sci.Technol. 41.6961-8.

Huwe, J. K., and D. J. Smith. 2007. Accumulation, whole-body depletion, and debromination of decabromodiphenyl ether in male sprague-dawley rats following dietary exposure. Environ.Sci.Technol. 41.2371-7.

Jenssen, B. M., E. G. Sormo, K. Baek, J. Bytingsvik, H. Gaustad, A. Ruus, and J. U. Skaare. 2007. Brominated flame retardants in North-East Atlantic marine ecosystems. Environ.Health Perspect. 115 Suppl 1.35-41.

Johansson, N., H. Viberg, A. Fredriksson, and P. Eriksson. 2008. Neonatal exposure to deca-brominated diphenyl ether (PBDE 209) causes dose-response changes in spontaneous behaviour and cholinergic susceptibility in adult mice. Neurotoxicology 29.911-9.

Kajiwara, N., Y. Noma, and H. Takigami. 2008. Photolysis studies of technical decabromodiphenyl ether (DecaBDE) and ethane (DeBDethane) in plastics under natural sunlight. Environ.Sci.Technol. 42.4404-9.

Knutsen, H. K., H. E. Kvalem, C. Thomsen, M. Froshaug, M. Haugen, G. Becher, J. Alexander, and H. M. Meltzer. 2008. Dietary exposure to brominated flame retardants correlates with male blood levels in a selected group of Norwegians with a wide range of seafood consumption. Mol.Nutr.Food Res. 52.217-27.

Kunisue, T., N. Takayanagi, T. Isobe, S. Takahashi, M. Nose, T. Yamada, H. Komori, N. Arita, N. Ueda, and S. Tanabe. 2007. Polybrominated diphenyl ethers and persistent organochlorines in Japanese human adipose tissues. Environ.Int. 33.1048-56.

Law, R. J., D. Herzke, S. Harrad, S. Morris, P. Bersuder, and C. R. Allchin. 2008. Levels and trends of HBCD and BDEs in the European and Asian environments, with some information for other BFRs. Chemosphere 73.223-41.

Legler, J. 2008. New insights into the endocrine disrupting effects of brominated flame retardants. Chemosphere 73.216-22.

Mariani, G., E. Canuti, J. Castro-Jimenez, E. H. Christoph, S. J. Eisenreich, G. Hanke, H. Skejo, and G. Umlauf. 2008. Atmospheric input of POPs into Lake Maggiore (Northern Italy): PBDE concentrations and profile in air, precipitation, settling material and sediments. Chemosphere 73.S114-S121.

Polder, A., G. W. Gabrielsen, J. O. Odland, T. N. Savinova, A. Tkachev, K. B. Loken, and J. U. Skaare. 2008a. Spatial and temporal changes of chlorinated pesticides, PCBs, dioxins (PCDDs/PCDFs) and brominated flame retardants in human breast milk from Northern Russia. Sci.Total Environ. 391.41-54.

Polder, A., C. Thomsen, G. Lindstrom, K. B. Loken, and J. U. Skaare. 2008b. Levels and temporal trends of chlorinated pesticides, polychlorinated biphenyls and brominated flame retardants in individual human breast milk samples from Northern and Southern Norway. Chemosphere 73.14-23.

Ross, P. S., C. M. Couillard, M. G. Ikonomou, S. C. Johannessen, M. Lebeuf, R. W. Macdonald, and G. T. Tomy. 2008. Large and growing environmental reservoirs of Deca-BDE present an emerging health risk for fish and marine mammals. Mar.Pollut.Bull.

Stapleton, H. M., B. Brazil, R. D. Holbrook, C. L. Mitchelmore, R. Benedict, A. Konstantinov, and D. Potter. 2006. In vivo and in vitro debromination of decabromodiphenyl ether (BDE 209) by juvenile rainbow trout and common carp. Environ.Sci.Technol. 40.4653-8.

Stapleton, H. M., and N. G. Dodder. 2008. Photodegradation of decabromodiphenyl ether in house dust by natural sunlight. Environ.Toxicol.Chem. 27.306-12.

Sudaryanto, A., N. Kajiwara, O. V. Tsydenova, T. Isobe, H. Yu, S. Takahashi, and S. Tanabe. 2008. Levels and congener specific profiles of PBDEs in human breast milk from China: implication on exposure sources and pathways. Chemosphere 73.1661-8.

Thomsen, C., H. K. Knutsen, V. H. Liane, M. Froshaug, H. E. Kvalem, M. Haugen, H. M. Meltzer, J. Alexander, and G. Becher. 2008. Consumption of fish from a contaminated lake strongly affects the concentrations of polybrominated diphenyl ethers and hexabromocyclododecane in serum. Mol.Nutr.Food Res. 52.228-37.

U.S.Environmental Protection Agency. Toxicological review of decabromodiphenyl ether (BDE-209). EPA and In support of summary information on the integrated risk information system (IRIS). CAS No.1163-19-5. 2008. Washington D.C.
Ref Type: Report

van den Steen, E., A. Covaci, V. L. Jaspers, T. Dauwe, S. Voorspoels, M. Eens, and R. Pinxten. 2007. Accumulation, tissue-specific distribution and debromination of decabromodiphenyl ether (BDE 209) in European starlings (Sturnus vulgaris). Environ.Pollut. 148.648-53.

van der Ven, L.T.M., van de Kuil T., P. E. Leonards, W. Slob, R. F. Canton, S. Germer, T. J. Visser, S. Litens, H. Hakansson, D. Schrenk, Berg M. van den, A. H. Piersma, J. G. Vos, and A. Opperhuizen. 2008. A 28-day oral dose toxicity study in Wistar rats enhanced to detect endocrine effects of decabromodiphenyl ether (decaBDE). Toxicol.Lett. 179.6-14.

Venier, M., and R. A. Hites. 2008. Flame retardants in the atmosphere near the Great Lakes. Environ.Sci.Technol. 42.4745-51.

Wong, M. H., S. C. Wu, W. J. Deng, X. Z. Yu, Q. Luo, A. O. Leung, C. S. Wong, W. J. Luksemburg, and A. S. Wong. 2007. Export of toxic chemicals - a review of the case of uncontrolled electronic-waste recycling. Environ.Pollut. 149.131-40.
