# Supplementary material for: Policy relevant Results from an Expert Elicitation on the Human Health Risks of Decabromodiphenyl ether (decaBDE) and Hexabromocyclododecane (HBCD)
Source: Environ Health. 2012 Jun 28;11(Suppl 1):S7. doi: 10.1186/1476-069X-11-S1-S7 (PMC3388476; doi:10.1186/1476-069X-11-S1-S7)
Supplement: Additional file 2_Q1_HBCD — Evaluation questionnaire – Causal chain for BFR HBCD Questionnaire 1 with explanation and background information. Online version is available at http://henvinet.nilu.no/EvaluationofKnowledge/tabid/1333/language/en-US/Default.aspx [file 1476-069X-11-S1-S7-S2.pdf]

Evaluation questionnaire – Causal chain for BFR HBCD

Introduction

Thank you very much for participating in this expert evaluation, conducted in the context of the HENVINET project.

With your help we will evaluate the state of the art in the scientific knowledge of various aspects of the cause-effect relationship between the production and use of Hexabromocyclododecane (HBCD) and its potential impact on health.

The goal is to identify knowledge gaps and potential agreement or disagreement on this between you and your expert colleagues in the field. Ultimately, the aim is to discuss the implications of the results of the evaluation for policy and research.

The evaluation consists of two separate parts. In part A, you will be asked to comment on the completeness and structure of the causal diagram illustrating scientists' current understanding of the cause effect relationship between the production and use of HBCD and its potential impact on health. In part B you will be asked to express your confidence in scientists' ability to predict various aspects of the health risk assessment of HBCD.

We expect the entire exercise will take you about 30-45 minutes.

Sometimes experts feel uncomfortable performing evaluations on the basis of their scientific intuition and experience. We ask for your considered opinion based on the quality of your scientific work and rely on the fact that your broad experience in the field will suffice to help achieve a qualified understanding of the issues under discussion here.

Your own expert judgement will be complimented by those of at least 10 of your colleagues in the field, as well as by a thorough review of the literature on this issue. All this information will be considered by a panel of experts during a workshop at a later time, on the impact of environment factors on health. On this basis, the experts will provide recommendations to policy makers with regards to research and problem solving strategies.

We hope that this will address any concerns you may have. If not, you may contact Gunnar Eriksen: gunnar.eriksen@vetinst.no

We appreciate your participation very much and, on behalf of the Norwegian Veterinary Institute, Norwegian School of Veterinary Science, WHO Euro, Norwegian Institute for Air Research and the HENVINET consortium, we thank you for your time.

Information about yourself

Please tell us about your research background and current institutional affiliation. These data will be confidential.

- Name:_______________________________________
- Email address: _______________________________________
- Institutional affiliation: _______________________________________
- 5 keywords describing your area of expertise:

Part A – Evaluation of structure and completeness of diagram

The diagram shown in the figure below illustrates the cause-effect relationship between production and emission of HBCD and health effect. For a summary explanation of the scientific basis of the diagram, please see Annex 1.

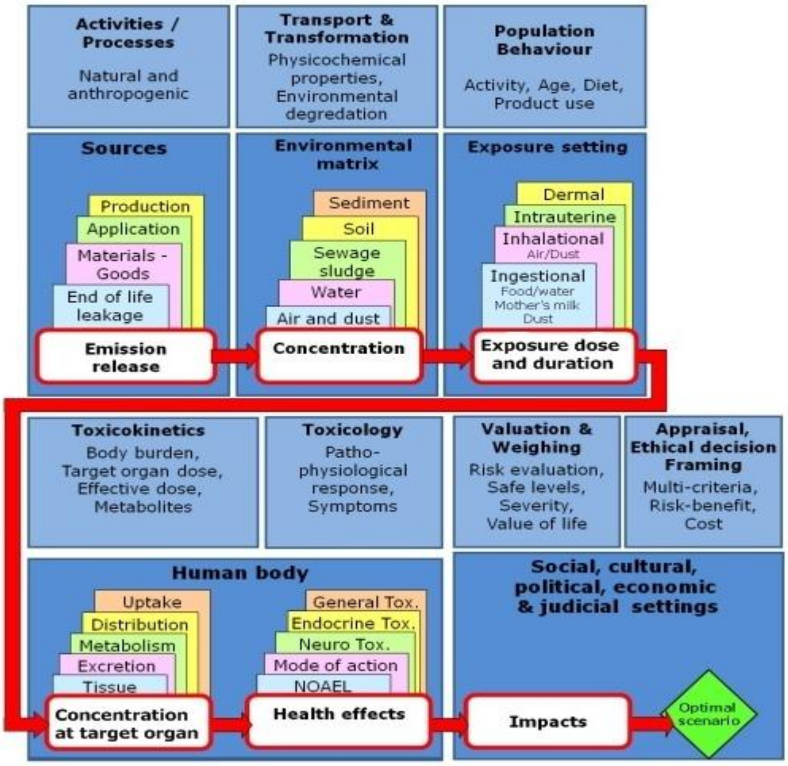

Does the diagram take into account all of the important parameters when evaluating the risks related to production, use and discharge of HBCD?*

○ Yes ○ No

If you said no to the previous question, Please explain:

Are the different causal relationships adequately structured?*

Yes No

If you said no to the previous question, Please explain:

Are there any unnecessary parameters shown in the diagram that could be deleted?*

Yes No

If you said yes to the previous question, Please explain:

Part B – Evaluation of individual model parameters

In the questions that follow you will be asked to express your confidence in scientist's ability to predict the concentrations, exposure and effects of HBCD Insert a check mark where you feel it is appropriate.

It is important that you consider each question independently of the others. For example, when you answer a question on sources, do not take into consideration your confidence in scientists' ability to predict exposure.

Where questions ask for your confidence level, please use these guidelines:

Very high - At least 9 in 10 chance of being correct
High - At least 7 in 10 chance of being correct
Medium - At least 5 in 10 chance of being correct
Low - At least 3 in 10 chance of being correct
Very low - 2 in 10 or less chance of being correct

Sources

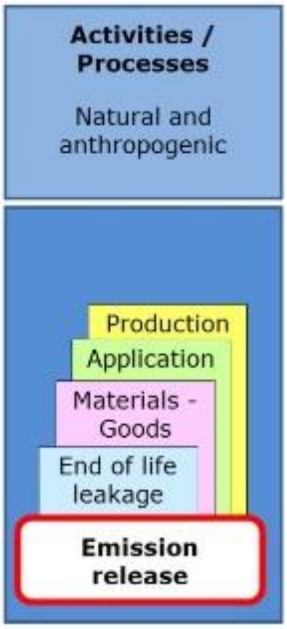

Regarding HBCD, what is your level of confidence in the quality of the current scientific data on:

Production volumes*

Very high	High	Medium	Low	Very low

Application volumes*

Very high	High	Medium	Low	Very low

Regarding the use of HBCD in products, what is your level of confidence in the scientists' ability to:

Identify and quantify all different applications*

Very high	High	Medium	Low	Very low

Predict the magnitude of emission/release/leakage during production, use and recycling*

Very high	High	Medium	Low	Very low

Environmental Matrix

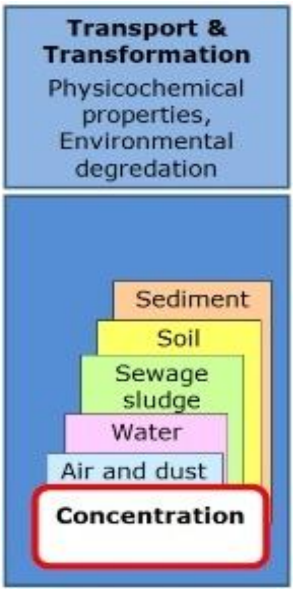

Regarding HBCD, what is your level of confidence in the scientists' ability to predict: Environmental transformation, such as conversion of diastereomers and biological half-lives?*

Very high	High	Medium	Low	Very low

The magnitude of long-range transport?*

Very high	High	Medium	Low	Very low

What is your level of confidence in the scientists' ability to predict the <u>concentration</u> of HBCD in:
Sediments?*

Very high	High	Medium	Low	Very low

Sewage sludge?*

Very high	High	Medium	Low	Very low

Soil?*

Very high	High	Medium	Low	Very low

Water?*

Very high	High	Medium	Low	Very low

Dust?*

Very high	High	Medium	Low	Very low

Indoor Air?*

Very high	High	Medium	Low	Very low

Outdoor Air?*

Very high	High	Medium	Low	Very low

Exposures

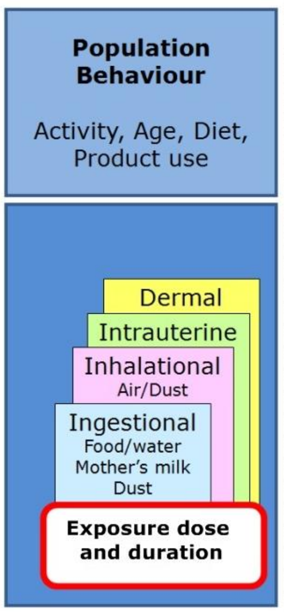

What is your level of confidence in the scientists' ability to predict the <u>level of exposure</u> to HBCD in:

The general population?*

Very high	High	Medium	Low	Very low

Occupationally exposed?*

Very high	High	Medium	Low	Very low

Infants and children?*

Very high	High	Medium	Low	Very low

What is your level of confidence in the scientists' ability to predict <u>the main sources of exposure</u> to HBCD in:

The general population?*

Very high	High	Medium	Low	Very low

Occupationally exposed?*

Very high	High	Medium	Low	Very low

Infants and children?*

Very high	High	Medium	Low	Very low

What is your level of confidence in the scientists' ability to predict the exposure of the general population to HBCD via the following routes:

Direct contact/dermal?*

Very high	High	Medium	Low	Very low

Inhalation?*

Very high	High	Medium	Low	Very low

Ingestion?*

Very high	High	Medium	Low	Very low

What is your level of confidence in the scientists' ability to predict the exposure of occupationally exposed groups to HBCD via the following routes:

Direct contact/dermal?*

Very high	High	Medium	Low	Very low

Inhalation?*

Very high	High	Medium	Low	Very low

Ingestion?*

Very high	High	Medium	Low	Very low

What is your level of confidence in the scientists' ability to predict the exposure of <u>infants and children</u> to HBCD via the following routes:

Direct contact/dermal?*

Very high	High	Medium	Low	Very low

Inhalation?*

Very high	High	Medium	Low	Very low

Intrauterine?*

Very high	High	Medium	Low	Very low

Via food?*

Very high	High	Medium	Low	Very low

Via breast milk?*

Very high	High	Medium	Low	Very low

Human Body – Toxicokinetics

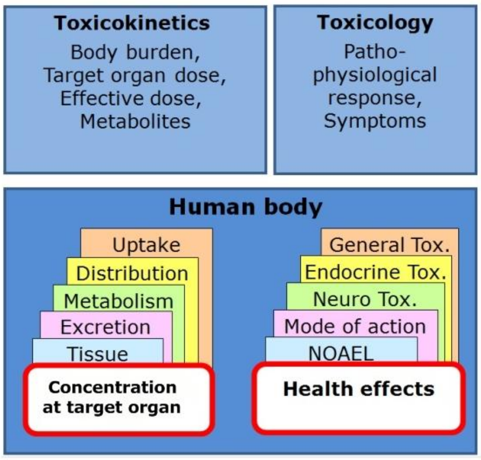

What is your level of confidence in the scientists' ability to predict to what extent HBCD is:

Absorbed/taken up?*

Very high	High	Medium	Low	Very low

Metabolised to other diastereomers after absorption?*

Very high	High	Medium	Low	Very low

Metabolised to hydroxymetabolites after absorption?*

Very high	High	Medium	Low	Very low

Metabolised to debrominated metabolites after absorption?*

Very high	High	Medium	Low	Very low

Accumulating in the body?*

Very high	High	Medium	Low	Very low

Excreted via bile and faeces?*

Very high	High	Medium	Low	Very low

Excreted via urine?*

Very high	High	Medium	Low	Very low

Regarding HBCD, what is your level of confidence in the scientists' ability to predict

The distribution to different tissues?*

Very high	High	Medium	Low	Very low

The final concentration of the parent compound in the target tissues, taking factors such as absorption, distribution, metabolism and excretion into account?*

Very high	High	Medium	Low	Very low

The final concentration of metabolites in the target tissues, taking factors such as absorption, distribution, metabolism and excretion into account?*

Very high	High	Medium	Low	Very low

The biological half-life?*

Very high	High	Medium	Low	Very low

Human Body - Toxicology

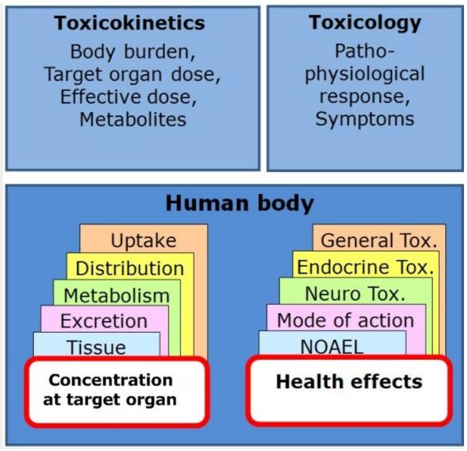

Based on <u>human epidemiological studies</u>, what is your level of confidence in the scientists' ability to predict adverse effects of HBCD in
Males?*

◯ Very high	◯ High	◯ Medium	◯ Low	◯ Very low

Females?*

◯ Very high	◯ High	◯ Medium	◯ Low	◯ Very low

Based on experimental animal studies, what is your level of confidence in the scientists' ability to predict adverse effects of HBCD on <u>general health</u> in
Males?*

◯ Very high	◯ High	◯ Medium	◯ Low	◯ Very low

Females?*

◯ Very high	◯ High	◯ Medium	◯ Low	◯ Very low

Based on experimental animal studies, what is your level of confidence in the scientists' ability to predict adverse effects of HBCD on <u>nervous system</u> in
Males exposed as adults?*

◯ Very high	◯ High	◯ Medium	◯ Low	◯ Very low

Females exposed as adults?*

◯ Very high	◯ High	◯ Medium	◯ Low	◯ Very low

Males exposed during foetal or neonatal life?*

◯ Very high	◯ High	◯ Medium	◯ Low	◯ Very low

Females exposed during foetal or neonatal life?*

◯ Very high	◯ High	◯ Medium	◯ Low	◯ Very low

Based on experimental animal studies, what is your level of confidence in the scientists' ability to predict adverse effects of HBCD on thyroid function in
Males exposed as adults?*

Very high	High	Medium	Low	Very low

Females exposed as adults?*

Very high	High	Medium	Low	Very low

Males exposed during foetal or neonatal life?*

Very high	High	Medium	Low	Very low

Females exposed during foetal or neonatal life?*

Very high	High	Medium	Low	Very low

Based on experimental animal studies, what is your level of confidence in the scientists' ability to predict adverse effects of HBCD on reproductive function in

Males exposed as adults?*

Very high	High	Medium	Low	Very low

Females exposed as adults?*

Very high	High	Medium	Low	Very low

Males exposed during foetal or neonatal life?*

Very high	High	Medium	Low	Very low

Females exposed during foetal or neonatal life?*

Very high	High	Medium	Low	Very low

Based on experimental studies, what is your level of confidence in the scientists' knowledge of the mechanisms of action of

HBCD?*

Very high	High	Medium	Low	Very low

[illegible]-HBCD?*

Very high	High	Medium	Low	Very low

[illegible]-HBCD?*

Very high	High	Medium	Low	Very low

[illegible]-HBCD?*

Very high	High	Medium	Low	Very low

Other metabolites of HBCD?*

Very high	High	Medium	Low	Very low

What is your level of confidence in the scientists' ability to predict the NOAEL of HBCD?*

Very high	High	Medium	Low	Very low

Final Comments

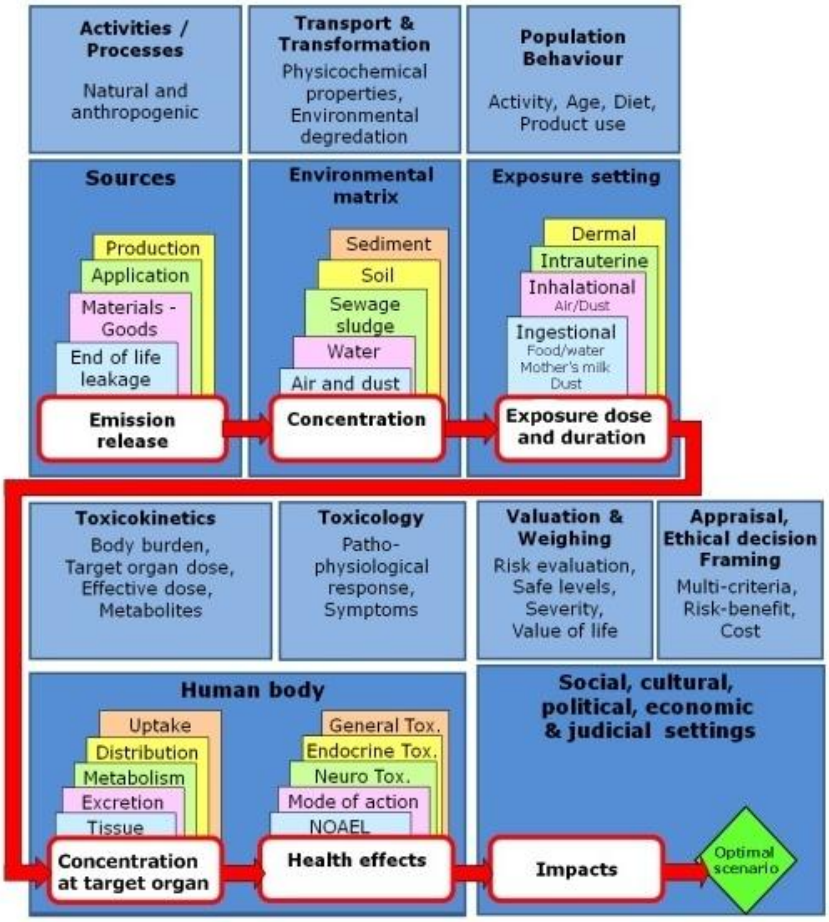

Final comment

Finally, do you think that any relevant questions were left out or that any questions were superfluous?
Please describe:

All evaluators will be acknowledged in the article that will be based on this questionnaire.

☐ Please check this box if you wish to remain anonymous.

Thank you for taking part in this evaluation and contributing your time and expertise. Your assistance is appreciated.

These results will be analysed and reviewed by experts within the HENVINET consortium.

Annex 1 Background information about Hexabromocyclododecane (HBCD)

This document is built up according to the cause-effect chain as defined by the Henvinet. It is intended as support for the expert evaluators when answering the questionnaire and will not be submitted to a scientific journal.

The literature reviewed was sampled after the following criteria:
PubMed searches: Review articles published in 2007 and 2008
Google searches: Reports from 2007 and 2008
PubMed searches: Research papers from 2008 and 2009 where the latest news in the field were needed, such as for toxicological effects.

The document is mainly based on Law et al. (2008) and the HBCD EEC Risk Assessment (2007).

Sources

Production

- Hexabromocyclododecane (HBCD) stands third in production volume (8.2%) of brominated flame retardants (BFRs) after tetrabromobisphenol A (TBBPA) (58.7%) and the decabromodiphenyl ether mixture (DecaDBE) (27.5%) (Zegers 2005).
- Total global production of HBCD in 2001 was 16,700 tons (BSEF 2007).
- Of the global production in 2001 about 60% were consumed in Europe and 20% in North America and 20% in Asia (Janak 2005 and Marvin 2006).
- HBCD is produced in USA, Europe and Asia. The sole production site today in Europe is in the Netherlands, with an annual production volume of 6000 tons in 2005 (HBCD Risk Assessment, EEC, April 2007).
- Commercial formulations of HBCD are 75-89% γ-HBCD, 10-13% α-HBCD and 1-12% β-HBCD.

Application
HBCD is used as a flame retardant additive first of all in polystyrene insulation foam, but in addition it is used in upholstery textiles and video or audio equipment castings. Finally, use has been reported in crystal and high –impact polystyrene, SAN (Styrene-AcryloNitrile) resins, adhesives, and coatings (EPA 2008).

Materials – Goods (HBCD Risk Assessment, EEC, April 2007).

- Expanded polystyrene (EPS) (major product)-Used for Insulation in: Construction, Insulation boards, Packaging material (minor, not food).
- Extruded polystyrene (XPS) (major product)-Used for Insulation in: Construction & Insulation boards.

- High impact polystyrene (HIPS) (minor product)-Electrical and electronic equipment in: VCRs, electric castings, distribution boxes, cassette castings.
- Polymer dispersion on cotton/synthetic blends-used as a textile coating agent: Upholstery fabric, bed mattress ticking, upholstered furniture, seatings, draperies, wall coverings, interior textiles, automobile indoor textiles.

End of life leakage

- HBCD is not covalently bonded to the material leading to the risk of migration out of the product during use or disposal (Tomy 2005).
- In a study of amount and presence of BFRs originating from electrical, electronic equipment and construction materials in a Swiss recycling plant, HBCD was measured to be 17 mg/kg of bulk waste (Morf 2007).
- A large and variable percentage of HBCDs in the atmosphere (69.1-97.3 %) existed in the particle phase, and suggest that long-range transport is possible in some environmental conditions (Yu 2008).

Environmental Matrix

HBCD are ubiquitous contaminants in the environment, wildlife and humans due to widespread use, low volatility and low water solubility (Covaci 2006).

Biotransformation and half-lives

- Biologically mediated transformation and anoxic conditions accelerate the rate of loss of HBCD. The biotransformation half-lives were 63 and 6.9 days in aerobic and anaerobic soils, and the biotransformation half-lives ranged from 11 to 32 days and 1.1 to 1.5 days in aerobic and anaerobic river system conditions (Davis 2005).
- Microorganisms naturally occurring in aquatic sediments and anaerobic digester sludge debrominate HBCD via dihaloelimination. Metabolites identified were tetrabromocyclododecane, dibromocyclododecadiene, cyclododecatriene (Davis 2006).
- Degradation of HBCD (a technical mixture) under anaerobic conditions in sewage sludge in a laboratory system gave a half-life of 0.66 days. Half-life of α-HBCD was double of β-HBCD and γ-HBCD (Gerecke 2006).

Sediment

- Total HBCD concentrations of North Sea surface sediments were from <0.2 to 6.9 µg/kg dry weight (n=10) (Klamer 2005).
- HBCD concentrations from the German Bight were from 0.03 to 6.5 µg/kg dry weight (n=12) (Lepom 2007).
- HBCD was only detected in the depth interval from 1 to 2 cm, α -HBCD = 0.43 µg/kg dry weight, γ -HBCD = 3.9 µg/kg dry weight, β -HBCD was not detected (n=4) (Evenset 2007). There has been a more than 5-fold increase in Sum-BDE concentrations in the lake sediments over the last 50 years.

- Three sediment cores and six surface sediment samples from Tokyo Bay were analysed (Minh 2007). Sum-HBCD was ranging from 0.06 to 2.3 µg/kg dry weight, implying widespread contamination (n=3). Levels were higher near to the highly populated industrial area of the bay indicating industrial and human activities as sources. HBCD first appeared in sediment cores in the mid-1970s and increased since then. The annual surface flux to sediments currently is: Sum-HBCD=0.6-2.4 ng/cm2/year.
- Swiss lake sediments showed that the concentration of HBCD was continuously increasing to reach 2.5 ng/g dry weight in 2001 (Kohler 2008).

Soil
Very little data is available on HBCD concentrations in soil.

- Concentration in soil from urban areas in China ranged from 1.7 to 5.6 ng/g dry weight (n=3) (Yu, Peng et al 2008).
- Soil samples from near-point sources in Sweden and Belgium/Germany ranged from 111 to 23,200 ng/g dry weight (Covaci 2006).

Sewage sludge

- HBCD was determined in sewage sludge from eight locations in the Czech Republic, and in sediments downstream of the sewage plant. HBCD concentration was 1-27 µg/kg dry weight (n=8) (Pulkrabova 2007).

Water
Data for BDEs in dissolved and suspended phases of water samples is usually not gathered because of their high degree of hydrophobicity, which will cause adsorption to particulate matter and deposition in sediments as potential sinks and sources (Law 2008).

Air
Outdoor air

- 13-15 pg/m3 Sum-HBCD (n=2) outside Japanese homes (Takigami 2007).
- 1.2-1.8 pg/m3 (n=4) in Guangzhou city in South China (Yu, Peng et al 2008). Mean percentages of β-HBCD, α-HBCD and γ-HBCD were 58%, 15% and 27% respectively, which differs from commercial mixtures and may be due to leaching from the high temperature treated products.

Indoor air

- Sum-HBCD = 6.7 and 280 pg/kg in indoor air in Japan (n=2) (Takigami 2007).

Long-range transport

- A large and variable percentage of HBCDs in the atmosphere (69.1-97.3 %) existed in the particle phase, and suggest that long-range transport is possible in some environmental conditions (Yu 2008).

Exposure

Humans can be exposed to HBCD by inhalation of vapour and airborne dust, through ingestion and by dermal contact. Babies can be exposed during pregnancy and breast-feeding. Workers and consumers are mainly exposed through inhalation and dermal routes, exposure via the environment is mainly through the oral route Netherlands (HBCD Risk Assessment, EEC, April 2007).

Intrauterine

HBCD can be transferred to infants through cord blood.

- A Dutch study of mothers (n=90) and infants (n=90) showed that HBCD was detected in almost all samples (Weiss 2004). Cord blood showed a mean of 2.4 ng/g lipid weight, a median of 0.32 ng/g lipid weight and a range of 0.16-4.2 ng/g lipid weight. Mothers' serum showed a mean of 1.1 ng/g lipid weight, a median of 0.72 ng/g lipid weight and a range of 0.16-6.9 ng/g lipid weight.

Mother's milk, human

HBCD is transferred to infants through mother's milk, and increased concentrations in the milk have been measured over time.

- One of the highest levels of HBCD in mother's milk was measured in Mexico with an average of 2.1 ng/g lipid (range of 0.8-5.4 ng/g lipid and n=7) (Lopez 2004).
- One of the lowest average levels measured were in Sweden in 1980 (average 0.084 ng/g lipid and n=116). The levels in the Swedish study were shown to increase until 2002 (average of 0.75 ng/g lipid and n=20), where after the levels decreased (average of 0.39 ng/g lipid and n= 20, measured in 2004) (Fangstrom 2005, Fangstrom 2006).

Food/Water

A typical exposure level of 3 ng HBCD/kg/day, a maximum level of 22 ng HBCD/kg/day, and a level of 20 ng HBCD/kg/day is considered in the risk characterization (HBCD, Risk Assessment, EEC, April 2007).

- A regional average concentration of HBCD in fresh water fish based on all EU data has been estimated to be 20 µg/kg wet weight. Based on this a daily intake of HBCD from fish is approximately 33 ng HBCD/kg bwt/day.
- A screening study on a limited number of different samples of food in Sweden (fish, chicken, milk and egg) and the amount of food normally consumed of these food types, resulted in a calculated estimated maximum intake of 22 ng HBCD/kg/day. The medium value was 10-fold lower (Lind 2002).

- An average dietary intake of HBCD in the Dutch population was estimated to be 3 ng HBCD/kg bwt/day, from measuring the concentration of HBCD in 91 samples of food like dairy, meat, animal fat, eggs, fish and vegetable oil (De Winter-Sorkina 2003).

Dermal
Only estimated values exist for dermal exposure.
Occupational, estimated (HBCD, Risk Assessment, EEC, April 2007).

- *Occupational exposure: Manufacture of HBCD:* 1-5 mg/cm^2/day, this is equivalent to 4200 mg/day if assuming exposure of two hands.
- *Occupational exposure: Industrial use of HBCD as an additive (formulation and processing in the polymer industry):* 84 and 120 mg/day for XPS/EPS-production and textile coating, respectively. For granules, the exposure is thought to be 10 % of that with powder, because of less dusting, i.e., 8.4 mg/day.
- *Occupational exposure: during industrial end-use of semi- and end-products containing HBCD:*A total exposure is estimated to be 84 mg HBCD/day

Consumer, estimated (HBCD, Risk Assessment, EEC, April 2007).

- *A consumer exposure assessment of HBCD* was made on dermal exposure assuming exposure from furniture upholstery back-coated with HBCDD, estimated to be 1.3×10^{-6} mg/kg/day.

Inhalation
Occupational, estimated (HBCD, Risk Assessment, EEC, April 2007).

- *Occupational exposure: Manufacture of HBCD:* A typical level of exposure via inhalation can be about 0.95 mg/m^3, representing 4h contact with the standard grade substance
- *Occupational exposure: Industrial use of HBCD as an additive (formulation and processing in the polymer industry):* For HBCD charging to a process, reasonable worst-case exposure levels for fine grade and standard grade HBCD is 2-5 mg/m^3.
- *Occupational exposure: during industrial end-use of semi- and end-products containing HBCD:* The air concentration is estimated to be 0.5 mg/m^3

Consumer, estimated (HBCD, Risk Assessment, EEC, April 2007).

Air

- *A consumer exposure assessment of HBCD* was made. Inhalation exposure in a room caused by wear of and evaporation of HBCD from fabric upholstery treated with HBCD, is estimated to give a total air concentration of 3.9 µg/m^3 HBCD.
- *Indoor air exposure*: Estimated to be 0.002 µg/kg bwt/day.

- *Mattress ticking:* Estimated to be 0.01 µg/kg bwt/day.

Dust

- *Oral Exposure to dust:* Assuming the daily amount of dust available for oral exposure would be 2.5 mg/day, the content of HBCD in the dust was 0.47 %, leading to an oral exposure to 12 µg HBCD/day. If a 10 kg child is eating all dust generated from the sofas, the daily exposure would become 1.2 µg/kg/day, the internal exposure will be 1.5 µg/kg bwt/day.
- *Oral Exposure to mouthing a textile:* Estimated to be 3 µg/kg bwt/day.

Consumer, measured:
Very little data has been reported. Different sampling methods can give different results (e.g. passive samplers like PUF disks that only collect the particulate phase and active samplers like Hi-Vols that primarily measures the gas phase) (Abdallah 2008).

Air

- Air in homes in Japan (n=2) SHBCD = 6.7 and 280 pg/kg indoor air (using Hi-Vol samplers) (Takigami 2007).
- Outdoor air in Guangzhou city in South China 1.2-1.8 pg/m^3 (n=4). Mean percentages of β-HBCD, α-HBCD and γ-HBCD were 58%, 15% and 27% respectively, which differs from commercial mixtures and may be due to leaching from the high temperature treated products (Yu, Peng et al 2008).

Dust

- Sum-HBCD α-, β-, γ-diastereomers in UK: house dust was on average = 6000 µg/kg (n=31), office dust was on average = 1400 µg/kg (n=6) indistinguishable from North American domestic dust. The diastereomer pattern in dust fell between commercial formulations (predominantly γ-) and human tissues (predominantly α-) (Abdallah 2008).
- Dust in homes in Japan (n=2) SHBCD=240 and 13,000 µg/kg in dust (Takigami 2007).

Toxicokinetics

HBCD is lipophilic and has a bioaccumulation factor log Kow of 5.6 and is considered bioavailable and bioaccumulative (Marvin 2006).

Uptake
The studies below were used for risk characterization; the oral and inhalation absorption were set to 100 % and 2-4 % for the dermal absorption, depending on the size of the particles (Yu & Atallah 1980, Roper 2005, HBCD, Risk Assessment, EEC, April 2007).

Inhalation
No studies are available on absorption through inhalation, but it was set to be 100% (HBCD, Risk Assessment, EEC, April 2007).

Oral
Animal studies demonstrate that HBCD can be absorbed from the gastro-intestinal tract.

- The estimated absorption half-life in a rat study was 2 hours from the gastrointestinal tract and peak radioactivity in blood was reached 4 hours after administration, and at 8 hours 43% of the administered dose was recovered in tissues. 93% of the dose was excreted within 3 days as transformed substance (metabolites), therefore an oral absorption close to 100% is indicated (Yu & Atallah 1980).
- Based upon a study in Labradors using felodipine (a substance similar to HBCD due to its poor water solubility), the LOAEL of HBCD in a rat study was adjusted to a corrected LOAEL based on an expected 10-20 % oral absorption (Chengelis 2001).

Dermal
The total dermal absorption was estimated to be 4%, based on a human *in vitro* skin test study (Roper 2005).

Distribution
The highest concentrations of HBCD are reached in adipose tissue and muscles followed by liver, and very little is found in lung, kidney, blood, brain, and gonads (Yu & Atallah 1980). During long-term exposures, females achieved higher concentrations than males (4342 µg/g fat in females and 3101 µg/g fat in males was measured in one rat study), but HBCD is bioaccumulating in both sexes (Chengelis 2001). The α-diastereomer is much more accumulating than the others (the relative bioaccumulation factor is 99:11:1 for α-, β- and γ-HBCDD, respectively) (Zegers 2004). It takes months to reach steady-state (HBCD, Risk Assessment, EEC, April 2007).

Tissue Levels
Adipose tissue and other organs, experimental animals
In a rat study using γ-HBCD, after 8 hours the highest concentration was found in adipose tissue, and muscle followed by liver. Very little was found in lung, kidney, blood and brain. After 8 hours 43% was recovered in tissues: 20% in fat, 14% in muscle, 7 % in liver and 0.2 % in gonads. After 24 hours 0.8% was found in the liver. After 48 hours 14% was found in fat, 3% in muscle and 0.5% in the liver. At 72 hours 14% was still found in fat, but the amount in muscle was reduced to 2% and the amount in liver to 0.28% (Yu & Atallah 1980).

Mother's milk (human)
One of the highest levels of HBCD in mother's milk was measured in Mexico with an average of 2.1 ng/g lipid (range of 0.8-5.4 ng/g lipid and n=7) (Lopez 2005). One of the lowest average levels measured were in Sweden in 1980 (average 0.084 ng/g lipid and n=116). The levels in the Swedish study were shown to increase until 2002 (average of 0.75 ng/g lipid

and n=20), where after the levels decreased (average of 0.39 ng/g lipid and n= 20, measured in 2004) (Fangstrom 2005, Fangstrom 2006).

Blood (human)
In a Dutch study of 90 mothers and newborns, cord blood showed a mean of 2.4 ng/g lipid weight and a range of 0.16-4.2 ng/g lipid weight (Weiss 2004). Mothers' serum showed a mean of 1.1 ng/g lipid weight and a range of 0.16-6.9 ng/g lipid weight. Similar levels were found in 5 mothers in Mexico (Lopez 2004) and in mothers in the Netherlands (Meijer 2008). Another Dutch (Weiss 2006) study showed that the blood samples contained mostly the α-HBCD diastereomer with only a few percents γ-HBCD .

Comment: The blood levels of HBCD in different studies are measured in plasma, serum or whole blood and given as ng/g lipid weight, ng/ml plasma or ng/g blood and are thus difficult to compare without taking into account the percentage of fat.

Metabolism
HBCD diastereomer ratios differ in environmental matrix and in biota from the commercial mixtures, with a shift from the more common stereoisomer γ-HBCD in the technical mixture to a prevalence of the α-HBCD stereoisomer (Covaci 2006).
In biota the cytochrome P450 system preferentially metabolises the γ- and the β-diastereomers, but not the α-diastereomer. α-HBCDD is not accumulated in tissue by stereoselective degradation, but through preferential accumulation or stereoselective uptake (Zegers 2004).

- In a 90-minutes incubation of HBCD with hepatic microsomes, the β- and γ-diastereomer seemed to decrease (69% and 60% decrease, respectively), whereas no significant disappearance of α-HBCDD was observed (17% decrease) (Zegers 2004). For β-HBCD and γ-HBCDD respectively, three and two bromine-containing metabolites could be observed. Hydroxy-metabolites of both the β-diastereomer and γ-diastereomer were found.
- In a rat study using γ-HBCDD, after 3 days 93% of the administered dose was excreted as metabolized HBCD (Yu & Atallah 1980).
- In a rat study the mean levels of HBCD after 89 days was 3101 µg/g fat for males and 4342 µg/g fat for females (Chengelis 2001). The concentration in females were always higher than in males (15-100% more). In addition a 100-fold higher relative bioaccumulation of the α-HBCDD diastereomer than the major γ-HBCDD diastereomer.

Excretion
Elimination of HBCD and its metabolites mainly occurred via faeces, with a minor part excreted in urine. Elimination from body fat appears to be markedly slower than from other tissues, with an elimination half-life of the three diastereomers possibly being in the order of weeks to months (HBCD Risk Assessment, EEC, April 2007).

- In a rat study (n = 8 females and 2 males), after 48 hours 94% in males vs 54 % in females of the administered dose was eliminated in faeces (Yu & Atallah 1980). After

72 hours 77% of the HBCD and its metabolites were found in faeces and only 16% in urine.

- In a rat study (n = 4 males), 24 hours post dosing, no urinary excretion of unchanged HBCD was found (Arita, Miyazaki & Mure 1983). Faecal excretion of 29-37 % of the administered amount was found to be the average daily rate.

Toxicology

General Toxicology

Acute toxicity
The HBCD substance tested has a very low acute toxicity by oral and dermal exposure, and it has not been possible to determine a LD50 value. The minimum oral lethal dose is > 20 g/kg in rats (Wilson and Leong 1977 and Lewis and Palanker 1978), and > 40 g/kg in mice (EPA 1990 and Tobe 1984). LD_{50} of dermal exposure is > 20 g/kg in rabbits (Wilson and Leong 1977 and Lewis and Palanker 1978). The acute toxicity by inhalation has not been investigated properly, but seems to be low.

Irritation
HBCD is mildly irritating for the eye, but not enough to classify as an eye irritant according to EU criteria (Wilson and Leong 1977 and Lewis and Palanker 1978). HBCD is not irritating to skin in skin irritation studies or to the respiratory system according to clinical symptoms in acute toxicity studies by the inhalation route (Wilson and Leong 1977 and Lewis and Palanker 1978).

Corrosivity
HBCD is not corrosive to skin, based on a rabbit study (Crown 1984).

Sensitisation
Human studies show that no skin reactions were observed (McDonnell 1972). Two animal studies performed on a composite of EU-marketed HBCD (1-50%, n= 20 and 12 or 30) gave negative results (a Magnuson-Kligman and a Local Lymph Node test), and showed that this composite of HBCD can not be considered to be sensitising (Wenk 1996 and Wolhiser & Anderson 2003). However, two positive animal studies performed in Japan on HBCD of unknown origin and purity (0.005-5%, n=10), indicates that such HBCD may contain sensitising constituents (Nakamura 1994 and Momma 1993).

Endocrine Toxicology

Liver effects

The only really consistent effect from HBCD exposure is liver weight increase in female rats, and in most studies also in male rats. Hepatic enzyme induction is clearly involved and likely the cause of weight increase.

- Liver weights were increased in both sexes in rat studies with doses from 0 to 940 mg/kg/day (Zeller and Kirsch 1969 & 1970, Chengelis 1997 & 2001, van der Ven 2006).
- HBCD exposure in male and female rats (0-100 mg/kg bw) resulted in decreased plasma alkaline phosphatase in females, decreased apolar retionoids in female livers and increased CYP19/aromatase activity in the ovaries (van der Ven 2009).
- Rats exposed to HBCD gave effects on phase I and II enzymes (CYP3A3 and UGT), lipid metabolism and cholesterol biosynthesis. A more efficient elimination process of HBCD in males was registered (Canton 2008).
- Rat microsomes exposed to HBCD gave an mRNA induction in CYP2B1 and CYP3A4, probably via PXR and CAR signalling pathways. Higher enzyme induction in females than in males (Germer 2006).
- Chicken hepatocytes exposed to HBCD gave effects on the mRNA level on the lipid regulation, the thyroid hormone pathway and phase I and phase II enzymes (L-FABP, THRSP, TTR, CYP2H1, CYP3A37 and UGT1A9) (Crump 2008).
- Juvenile Rainbow Trout exposed to HBCD had effects on biotransformation enzymes (reduced CYP1A1 activity and induced glucuronosyltransferase (UDPGT) activity). All diastereomers (α-, β- and γ-) showed effects, with some differences in levels (Palace 2008).

Thyroid effects

The main endocrine disrupting effect of HBCD is on the thyroid hormone metabolism and the hypothalamo-pituitary-thyroid axis.

- Thyroid hyperplasia was observed in both sexes in a rat study with HBCD at 940 mg/kg/day (Zeller and Kirsch 1969). Thyroid and pituitary weight was increased in female rats in a study using HBCD from 0-200 mg/kg/day (van der Ven 2006). No thyroid effects were observed in a recent rat study using HBCD from 0-100 mg/kg/day (van der Ven 2009).
- Serum T4 was decreased and TSH was increased in female rats in a study using HBCD at 0-200 mg/kg/day (van der Ven 2006).
- In the presence of T3 (50 ng/ml), HBCD (3.12, 6.25, 12.5 and 25 μM) increased Thyroid receptor (TR)-mediated gene expression in HeLaTR cells (Yamada-Okabe 2005).
- Exposure of *Xenopus laevis* tadpole tail tips to 1000 nM HBCD in combination with 20 nM T_3, potentiated tail tip regression with 35% +/- 5% (Schriks 2006).
- HBCD significantly enhanced the number of proliferating cells in the brain of *Xenopus Laevis* tadpoles at the two highest doses 100 and 1000 nM (in combination with 1 nM T3) with 33.2 % and 24.5 %, respectively (Schriks 2006).

- Juvenile Rainbow Trout exposed to HBCD gave a transient disruption of the thyroid axis (reduced T4ORD activity (Deiodinase: T4->T3), lower FT4 activity, higher FT3 activity). All diastereomers (α-, β- and γ-) showed effects, with some differences in levels (Palace 2008).

Steroid hormone receptor effects

- Rat pituitary cells exposed to exhibits antiandrogenic (AR) (γ-HBCD, IC50 = 3.7 µM), antiprogesteronic (PR) (γ-HBCD, IC50 = 1.4 µM), T_3-potentiating properties (α-HBCD and γ-HBCD) and a low binding to transthyretin (TTR) to compete with T4 (α-HBCD and β-HBCD, EC_{50} = 12-15 µM) (Hamers 2006).

- HepG2 cells exposed to HBCD (0.03–0.3 ng/ml) resulted in inhibition of mRNA expression of two oestrogen responsive genes (ERα and THRα) (Aniagu 2008).

Reproduction Toxicology

Developmental toxicity
Two ordinary developmental toxicity studies have failed to demonstrate any fetotoxicity, teratogenic potential or adverse effects from HBCD on development of rats. 0-750 mg/kg/day (n=20) and 0, 500 or 1000 mg/kg/day (n=25) was given orally (Murai, Kawasaki & Kanoh 1985 and CMA & Chemical Manufacturers Association Brominated Flame Retardant Industry 1999).

Fertility
The available data from rats indicate effects on reproductive organs only at high exposure levels. But the high bioaccumulation of HBCD and the potential for milk transport are reasons to investigate further the full life-time toxicity. Recent data in a human study shows effects on the sexual development from lower levels of HBCD.

- A two-generation reproductive toxicity study in 24 rats (F0) was given 0, 150, 1500 or 15000 ppm HBCD (Ema 2008). Effects were not found on sex hormone-dependent events. Changes were found on the thyroid hormone axis (T4, TSH, FSH), liver enzymes (CYP2B1 and CYP2B2), liver size in (females). Results suggest that HBCD is potentially reproductively toxic, but no adverse effects on reproductive parameters in F1 dams or F2 pups were noted. The NOAEL was 10.2 mg/kg bwt/day = 150 ppm which is far below estimated human daily intake.
- A recent study in the Netherlands shows effects of prenatal exposure of HBCD on sexual development in healthy infants (Meijer 2008). Sex hormone levels like luteinizing hormone and testosterone were influenced from prenatal exposure (n=21-33). Testes volume and penile length were also affected from prenatal exposure (n=36).
- A recent study on rats showed reproductive effects of HBCD (0-100 mg/kg bw), like increased CYP19/aromatase activity in the ovaries (n=2) and also decreased weight of the testis (n=10) (van der Ven 2009).

Developmental neurotoxicity
Neonatal HBCD exposure may cause developmental neurotoxic effects due to observed statistically significant changes in spontaneous behaviour, learning and memory defects in two rat studies (0.9 or 13.5 mg/kg bw and n=10 or 0-100 mg/kg bw). A LOAEL of 0.9 mg/kg/day was determined, but this needs to be confirmed by other laboratories (Eriksson 2006). A recent human study of prenatal exposure has shown effects on psychomotor development (Meijer 2008).

Neurotoxicology
Neurotransmitter effects

- HBCD inhibit the plasma membrane uptake of glutamate and dopamine (IC50 = 4µM) and the vesicular uptake of dopamine (IC50 = 3µM) (Fonnum 2006, Mariussen and Fonnum 2003).
- Exposure to HBCD (0-20 µM) dose-dependently inhibits depolarization-induced increase in calcium levels and neurotransmitter release in a neuroendocrine *in vitro* model using rat pheochromocytoma (PC12) cells (Dingermans 2008).

Other effects

- Increased IgG response in males, increased fraction of neutrophilic granulocytes in males, decreased trabecular bone density of tibia in females was found in a recent study on rats using HBCD (0-100 mg/kg bw), (female rats, n=2) (male rats, n=10) (van der Ven 2009).
- Zebrafish exposure to HBCD gave increased Hsp70 (heat shock protein) and SOD (superoxide dismutase) EC/LC50 > 100 mg/l (Hu 2008).
- HepG2 cells were exposed to HBCD (0.5–10 µg/ml) and cell viability was measured (Zang 2008). γ-HBCD was more cytotoxic than β-HBCD that was more cytotoxic than α-HBCD. The (+) enantiomers were more cytotoxic than the (-) enantiomers.

Mutagenicity

The evidence from available studies indicates that HBCD lacks significant genotoxic potential *in vitro* as well as *in vivo* (TSCATS 1990e, TSCATS 1990a, Gudi and Schadly 1996, BASF 2000 and HBCD Risk Assessment, EEC, April 2007). HBCD induces genetic recombination in *in vitro* assays in mammalian cells indicating a potential to cause cancer via a non-mutagenic mechanism (Helleday 1999), but the relevance of the study is considered questionable due to lack of relevant information and low recombination activity (Ausgabe 2001).

Carcogenicity

No adequately performed HBCD cancer study has been reported. On long-term study with restricted validity in 50 male and 50 female mice, no evidence of carcogenicity was found in doses up to 1,300 mg/kg bwt/day (Kurokawa 1996 and HBCD Risk Assessment, EEC, April 2007).

Gender aspects

At long-term exposure, higher concentrations of HBCD in fat tissue are achieved in females than in males, but the substance is bioaccumulating in both sexes.

- Fat tissue levels of HBCD 20 male and 20 female rats (0 or 1000 mg HBCDD/kg/day orally for up to 90 days) (Chengelis 2001). The highest concentration of α-HBCDD in both sexes was at day 89, with mean levels of 3101 μg/g fat for males and 4342 μg/g fat for females.
- The excretion of γ-HBCDD was investigated in 8 female and 2 male rats after an oraldose (Yu & Atallah 1980).
- Rat microsomes exposed to HBCD gave an mRNA induction in CYP2B1 and CYP3A4, more in females than in males (Germer 2006).
- Rats exposed to HBCD gave effects on phase I and II enzymes (CYP3A3 and UGT), lipid metabolism and cholesterol biosynthesis (Canton 2008). A more efficient elimination process of HBCD in males was registered.
- HBCD exposure in male and female rats (0-100 mg/kg bw) resulted in decreased plasma alkaline phosphatase in females, decreased apolar retionoids in female livers, increased CYP19/aromatase activity in the ovaries, increased IgG response in males, increased fraction of neutrophilic granulocytes in males, decreased trabecular bone density of tibia in females (female rats, n=2) (male rats, n=10) (van der Ven 2009).

NOAEL

- The HBCD substance tested has a very low acute toxicity by oral and dermal exposure, and it has not been possible to determine a LD50 value. The minimum oral lethal dose is > 20 g/kg in rats (Wilson and Leong 1977 and Lewis and Palanker 1978), and > 40 g/kg in mice (EPA 1990 and Tobe 1984). LD_{50} of dermal exposure is > 20 g/kg in rabbits (Wilson and Leong 1977 and Lewis and Palanker 1978). The acute toxicity by inhalation has not been investigated properly, but seems to be low.
- From a study measuring fetotoxic and teratogenic potentials, a foetal and maternal NOAEL of 1000 mg/kg/day was determined (CMA & Chemical Manufacturers Association Brominated Flame Retardant Industry 1999).
- The effects on the liver, especially in the female rats, indicate a LOAEL of 125 mg/kg/day (Chengelis 1997).

- From observing effects on liver, thyroid and prostate in rats, a LOAEL = 10-20 mg/kg/day was concluded (Chengelis 2001).
- From measuring liver enzyme induction in female rats, the NOAEL/BMD-L of 22.9 mg/kg/day is proposed (van der Ven 2006). The same authors proposed Benchmark doses (BMD-L) for female rats: for the increased thyroid weight a BMD-L of 1.6 mg/kg/day, for the liver enzyme for T4-conjugation (T4-UGT) a BMD-L of 4.1 mg/kg/day, for the increased pituitary weight in female rats a BMD-L of 29.9 mg/kg/day.
- From a two-generation reproductive toxicity study in rats, the NOAEL was set to 10.2 mg/kg bwt/day = 150 ppm which is far below estimated human daily intake (Ema 2008).
- From a study of neonatal male NMRI mice, a LOAEL of 0.9 mg/kg/day was determined, but this needs to be confirmed by other laboratories (Eriksson 2006).
- HBCD inhibited plasma membrane uptake of glutamate and dopamine (IC50 = 4 µM) and the vesicular uptake of dopamine (IC50 = 3µM) (Fonnum 2006, Mariussen and Fonnum 2003).

Modes of action

Several endpoints for HBCD effects have been analysed lately, such as enzymatic, endocrinologic and histopathologic. The only really consistent effect was liver weight increase in female rats, and in most studies also in male rats. Hepatic enzyme induction is clearly involved and likely the cause of weight increase. Studies of the thyroid system have shown either effect in both sexes, only in females, or no effects (HBCD Risk Assessment, EEC, April 2007). Decreased serum thyroxine (T4) and increased serum TSH was observed (Chengelis 2001, Germer 2006 and van der Ven 2006). Similar thyroid hormone (TH)-related effects were seen in a two-generation study in rats (Ema 2008), and in addition reproduction related effects such as histology of the ovary and viability of the pups.

- Changes in liver and thyroid hormone system and prostate could possibly be explained by enzyme induction in the liver, since hepatic glucuronidation enzymes like T4-UGT transferase is known to be the rate limiting step in the biliary excretion of T4 (HBCD Risk Assessment, EEC, April 2007). T4-UGT transferase involved in the metabolism of T3/T4 is induced by HBCD in both sexes (van der Ven 2006). A hypothesis that could be supported from studies in female rats is that the first effect is an enzyme induction followed by an activation of the pituitary (resulting in TSH synthesis), and followed by an activation of the thyroid (hyperactive cells/weight increase) and finally if the T4/T3 decreases it can have effects other tissues and systems (HBCD Risk Assessment, EEC, April 2007).
- Another hypothesis is that instead of affecting the thyroid system via hepatic enzyme induction, HBCD acts via steroid hormone receptors like the progesterone receptor and the androgen receptor where HBCD exerts antagonistic effects (Hamers 2006).

- Also, binding of HBCD to thyroxine binding transport protein (TTR) could displace T4 from TTR, making T4 more susceptible to metabolism and excretion (Hamers 2006, Yamada-Okabe 2005, Schriks 2006).
- Developmental neurotoxicity was observed in mice (Eriksson 2006), and in humans (Meijer 2008), and possible roles of neurotransmitter inhibition has been shown (Dingermans 2008, Fonnum 2006 and Mariussen and Fonnum 2003).

Altogether these effects point towards a possible role of HBCD as a (neuro-) endocrine disruptor affecting the hypothalamo-pituitary-thyroid axis.
