# Supplementary material for: Policy relevant Results from an Expert Elicitation on the Human Health Risks of Decabromodiphenyl ether (decaBDE) and Hexabromocyclododecane (HBCD)
Source: Environ Health. 2012 Jun 28;11(Suppl 1):S7. doi: 10.1186/1476-069X-11-S1-S7 (PMC3388476; doi:10.1186/1476-069X-11-S1-S7)

HENVINET
HEALTH AND ENVIRONMENT NETWORK

Follow-up questions on decaBDE

Introduction

Thank you very much for participating in this expert evaluation, conducted in the context of the HENVINET project.

With your help we will further interpret the results of the first evaluation of the state of the art in the scientific knowledge of various aspects of the cause-effect relationship between the production and use of the brominated flame retardant decaBDE and the potential impact on health. You will find the causal diagram on the following page and a summary of the results of the first evaluation following that. The goal of this questionnaire is to identify priorities for further action and to discuss the implications of the results of the evaluation for policy and research. The outcomes of this questionnaire will form the basis for the workshop at the offices of WHO Euro in Copenhagen on May 19th.

In this questionnaire we will first ask you to pinpoint priority elements in the causal diagram. We will then ask you a series of four questions dealing with the implications of the results of the first evaluation on these priority elements. Issues such as research needs and the policy actions justified will be explored.

In the expert workshop on May 19th, a synthesis of the results of the two questionnaires will be presented and discussed in order to arrive at expert advice for EU-policymakers. This advice will be presented during a final stakeholder workshop and discussed in view of enriching the advice with societal viewpoints.

Please return this questionnaire no later than the 11th of May to Hans Keune at hans.keune@ua.ac.be.

We appreciate your participation very much and, on behalf of the the Nowegian Insitute for Veterinary Science, WHO Euro and the Henvinet consortium, we thank you for your time.

Follow-up questions on decaBDE

Questionnaire

1. In the table below, list the five most important elements of the causal diagram for decaBDE(see annex 1)*. Prioritize according to their influence on the extent of the health risk the causal chain leads to. If a small change in the value of an element results in a large change in the health impact, then this element has a high influence and should be considered very important. Conversely, if a large change in the value of an element leads to only small changes in the health impact, then this element has little influence and is not so important.
You may rank no more then two elements equally; five elements in total.

Priority	Label of element	Please explain why you attribute this priority to this element
1.		
2.		
3.		
4.		
5.		

**If you consider elements that are not represented in the causal diagram to be amongst the five most important, you may include these in the priority list. In Annex 1(after the causal diagram) you will find elements suggested by your colleagues as important supplements to the causal diagram we presented.*

Follow-up questions on decaBDE

2. Different strengths of evidence justify different policy intervention. For example, a high level of evidence is required to justify banning a substance, while a lower level of evidence might be sufficient to justify initiating a targeted monitoring program or a mandatory labelling scheme.

For each of the priority elements you have identified, indicate the type of action you consider is justified by the evidence available.

Causal elements*	*Conduct Scientific research*					*Policy action*<br>Concrete action by policymakers				Please explain the basis for your choice. If you have any specific scientific studies or hypotheses, please specify them here. If there are any broad implications for science, please explain. If you have any specific policy actions in mind, please specify them here.
	Fundamental science to gain knowledge about the problem			*Applied science* to gain knowledge about solving the problem						
	More data	Better data	Better under-standing	Developing interventions	Experimenting with interventions in practice	Monitor-ing	Awareness raising	Restricting risk activities	Prohibiting risk activities	
1.										
2.										
3.										
4.										
5.										

**As ranked by you in question 1*

Follow-up questions on decaBDE

Before answering the following questions, please take a moment to consider the "big picture" depicted by the overall results of the first evaluation on decaBDE (provided in annex 2).

With this in mind:

3. What is your level of confidence that conducting more scientific research would yield <u>decisive</u> knowledge on the risks of decaBDE within the next five years? *(decisive knowledge is understood here as knowledge that would clearly dictate which type of policy action is to be undertaken(or not))*

Insert checkmark in appropriate box					Please justify your answer. Also, if you expect decisive knowledge to become available, please specify which of the five causal element(s) you selected in question 1 this knowledge would most likely pertain to.
Very high	High	Medium	Low	Very low	

Interpretive guidance: Very high - At least 9 in 10 chance of being correct; High - At least 7 in 10 chance of being correct; Medium - At least 5 in 10 chance of being correct; Low - At least 3 in 10 chance of being correct; Very low - 2 in 10 or less chance of being correct

4. What is your level of confidence in the possibility that policy actions to effectively manage the health risks of decaBDE will become technically (not politically) feasible within the next five years? *In other words, are effective policy actions technically feasible now, or to what extent would you expect them to become feasible within the next 5 years?*

Insert checkmark in appropriate box					Please explain why.
Very high	High	Medium	Low	Very low	

Interpretive guidance: Very high - At least 9 in 10 chance of being correct; High - At least 7 in 10 chance of being correct; Medium - At least 5 in 10 chance of being correct; Low - At least 3 in 10 chance of being correct; Very low - 2 in 10 or less chance of being correct

Follow-up questions on decaBDE

5. As mentioned above, different strengths of evidence justify different policy intervention.

Considering the overall results of the evaluation, to what extent do you think the current scientific knowledge of the the health risks of decaBDE represents sufficient evidence to justify policy action (or not)?

Insert checkmark in appropriate box

Insufficient evidence to justify a policy intervention	Sufficient evidence to justify not taking policy action	Sufficient evidence to justify a policy intervention

Please explain why you choose this option.

Thank you!!

ANNEX1: Results, Part A: Evaluation of the structure and completeness; Causal diagram for decaBDE

Figure 1: Causal diagram for decaBDE

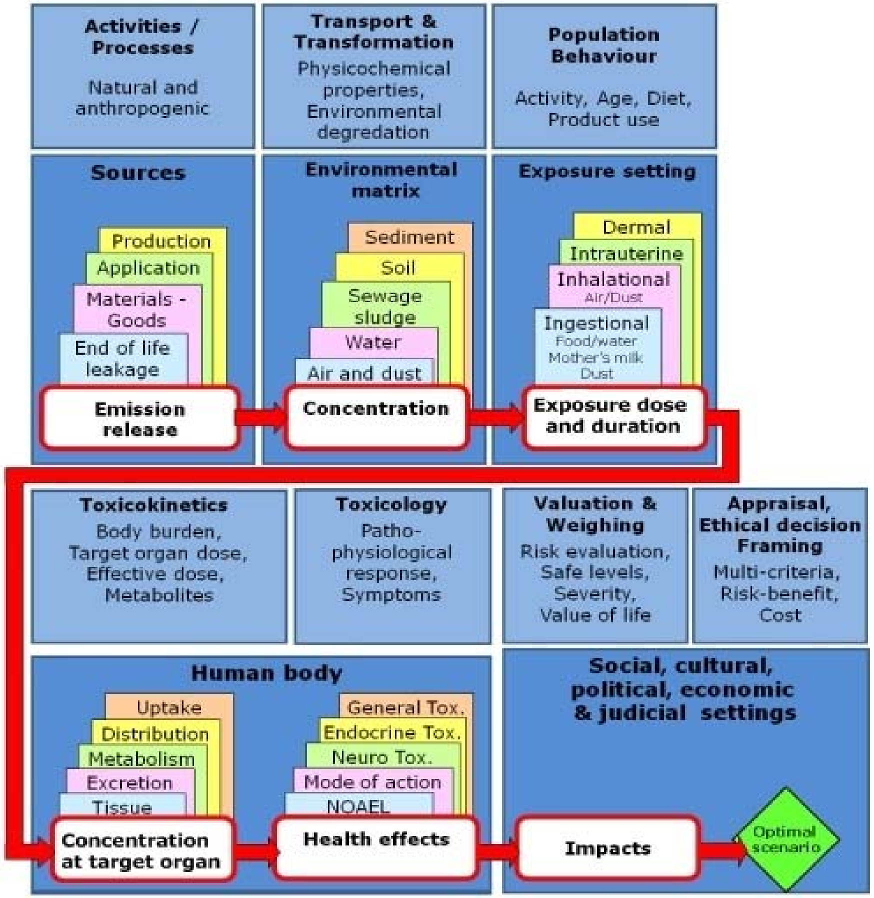

Supplement: Additional file 3_Q2_DecaBDE — Follow-up questions on decaBDE Questionnaire 2 on decaBDE. Experts were expected to use results of questionnaire 1 (Additional file 3) when answering. [file 1476-069X-11-S1-S7-S3.pdf]
