# Supplementary material for: Policy relevant Results from an Expert Elicitation on the Human Health Risks of Decabromodiphenyl ether (decaBDE) and Hexabromocyclododecane (HBCD)
Source: Environ Health. 2012 Jun 28;11(Suppl 1):S7. doi: 10.1186/1476-069X-11-S1-S7 (PMC3388476; doi:10.1186/1476-069X-11-S1-S7)
Supplement: Additional file 5_Policy Brief_decaBDE — HENVINET Policy Brief. Expert Elicitation on Health Implications of decaBDE. Based on the results from questionnaire 2 and the workshop, a policy recommendation was written as the final product of the project. [file 1476-069X-11-S1-S7-S5.pdf]

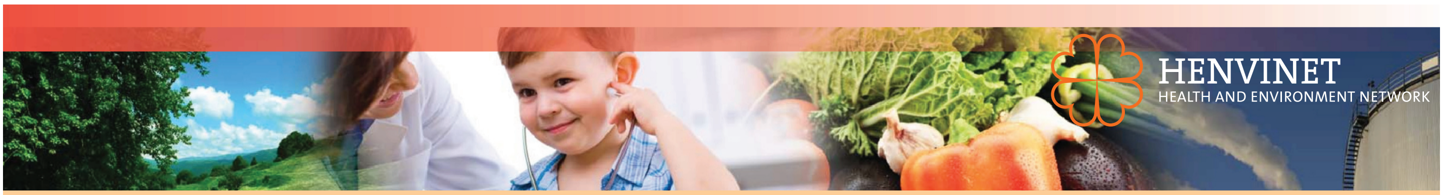

HENVINET Policy Brief:

Expert Elicitation on Health Implications of decaBDE

Policy context

- Deca-brominated diphenyl ether (decaBDE) is a flame retardant widely used in products such as electronics and textiles to impede development of fire and thereby save lives.
- DecaBDE is persistent in the environment, but differs from other polybrominated diphenyl ethers (BDEs) with respect to some important physicochemical properties: it is less absorbable into human and animal tissues; it accumulates less in these tissues; and it has a lower level of toxicity. On this basis, decaBDE has been less strictly regulated in many countries than other BDEs.
- There is a substantial build-up of decaBDE and a high predominance of this congener compared to other BDEs in some environmental compartments such as sediments, soils and dust. One concern relates to data demonstrating that decaBDE, under such circumstances can be broken down to other brominated compounds already banned. Another concern is to what extent microorganisms in the intestines and metabolism in the body are capable of transforming decaBDE to more toxic and bioaccumulating BDEs or other potentially harmful metabolites.
- The relatively high levels in the environment may lead to risk for substantial human exposure. In particular, the predominance of decaBDE in house dust may be a major exposure route for small children.
- Toxicological effects observed in animal studies include effects such as disruption of the development of the neurological system and hormonal balance at doses relevant to humans.
- Knowledge of the potential risks of the alternative chemicals to decaBDE is limited.

Policy options

In order to evaluate the state of the current scientific knowledge and highlight important policy considerations, experts were approached by two questionnaires followed by a workshop. Based on the answers from the questionnaires and discussion at the workshop, it was concluded that:

- All experts agreed that more research and monitoring are needed in order to develop a better understanding of the risks involved in the use of decaBDE.
- Experts agreed that three priority areas to investigate are:
  - I. The extent to which the substance is transformed to compounds with more tissue accumulating and toxic properties in the environment (other OH-BDEs and PDBEs with lower bromine content);
  - II. The extent to which humans and animals are exposed to the compound, especially from food and dust;
  - III. The extent to which decaBDE is transformed to more harmful substances in the human body.

This is to some extent supported by recent reviews and reports

- Effort should also be invested into research on the toxicity and environmental behaviour of the most frequently proposed alternatives to decaBDE before they are applied on a large scale.
- In order to accelerate the rate at which policy relevant information becomes available, experts feel that research collaborations between publically funded institutions and universities should be organised at the European level. In addition to publically funded research, industry should be required to provide more toxicological data.
- There was disagreement among the experts as to whether additional research would yield decisive knowledge on key issues related to decaBDE and its alternatives within five years, given adequate resources. Whereas most were either optimistic or meant that there already is sufficient decisive knowledge available, others stated that research requires more time. Most experts moreover had a medium to high degree of confidence that policy actions to effectively manage the health risks of decaBDE are technically (not necessarily politically) feasible either now, or will become so within the next five years.
- While there was disagreement, the majority of experts felt that, in light of the current, all be it limited, knowledge available on the risks of decaBDE, a precautionary ban or restrictions on the use of decaBDE are warranted.

Executive summary

Situation

Brominated flame retardants are used in many different consumer products with the aim of retarding development of fire and thereby save lives and reduce material damage (www.bsef.com). One group of brominated flame retardants is the polybrominated diphenyl ethers (BDEs). The different types of BDEs differ with respect to the number and position of bromine atoms in their molecule. DecaBDE, also known as BDE209, has the highest possible number of bromine atoms. The technical mixture of decaBDE contains small amounts of the nonaBDEs, 3% or less [1]. This mixture is almost exclusively used in electrical and electronic equipment, transportation sector, construction and building, and textiles [2].

Different research and policy communities have different points of view regarding the potential hazards of decaBDE. Penta- and octabrominated diphenyl ethers (penta- and octaBDEs) were found to accumulate in animal and human tissues and to cause harmful health effects, and were banned in the EU in 2004. The primary North American manufacturer voluntarily ceased their production [3]. The fully brominated BDE congener, decaBDE was regarded less toxic and was eluded from the ban [3]. In 2008, the European Court of Justice decided that the Commission had exempted decaBDE from the ban on false premises and consequently again a ban was put to its use in electrical and electronic products[4]. In Norway, a total ban was introduced in April 2008. Also, the states of Maine and Washington have restricted the use of the substance in certain products, but still many major uses of deca-BDE are allowed in North-America [2].

Since 2005, many companies have reduced the use of or phased out decaBDE voluntarily without specifying which flame retardants they use as substitutes. The main alternatives being proposed for decaBDE are other brominated compounds, phosphorus containing flame retardants and inorganic, non-phosphorus compounds. Data of the potential risks of these alternatives are limited.

Background

DecaBDE (BDE209) has shown in several studies to be the most abundant PBDE in sediments, sewage sludge, soil, dust and air [5,6]. Also, it shows a build-up over years in sediments [6]. An increasing number of studies show that decaBDE is being transformed into more accumulating, more toxic substances in some environmental matrices in processes involving e.g. microorganisms and sunlight [6,7]. Inhaled and ingested dust is probably the main route of exposure, together with ingestion of food, while direct dermal contact may also play an important role [8]. The developing foetus and infant will also be exposed through placenta and via mother's milk [1,8]. DecaBDE is absorbed from the intestines to a lesser extent than the other BDEs [9] and when absorbed it is distributed differently. That is, it is measured in relatively higher concentrations in blood and in the liver than in fat tissue which is the primary site of accumulation for the lower brominated compounds [1]. DecaBDE also accumulates to a lesser extent than other PBDEs in the body. Animal experiments have shown that decaBDE may be metabolised into more toxic and accumulating BDEs in the gut by microorganisms before absorption, as well as in the liver after absorption [1]. The presence of highly brominated metabolites not found in technical mixtures of BDE in human plasma [10] may indicate debromination also in humans, though exposure to environmentally

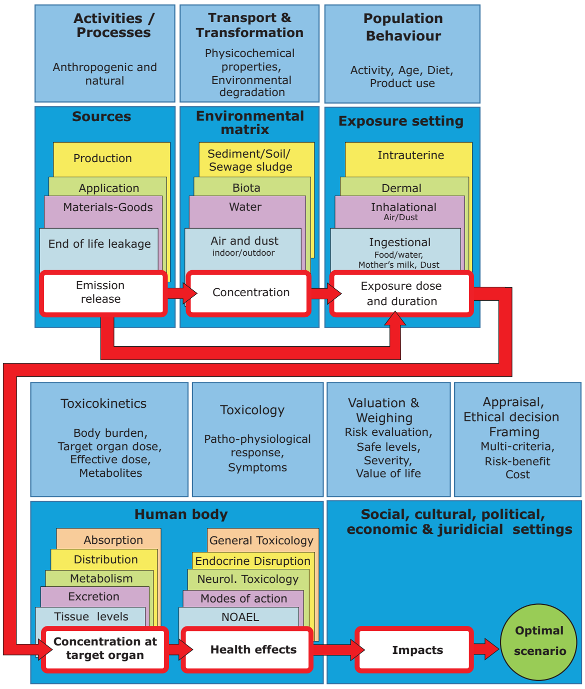

Figure 1. Diagram developed by HENVINET and used by experts to evaluate the current understanding of the cause-effect relationship between the production and use of decaBDE and its potential impact on health. The diagram has been slightly adapted to comments from the experts.

formed metabolites is also a possibility [11].

DecaBDE also appears to be excreted more rapidly from the body than the lower brominated BDEs [9]. Subchronic studies in rats have showed toxicological effects only in animals exposed to much higher doses compared to the other PBDEs [9] . More recent studies have been focussing on exposure to lower doses, closer to the real-life scenario during sensitive time frames of development and observed effects on neurobehavioural endpoints [9,12] and the thyroxin hormone balance [1,13]. There are not many existing effect studies and some are also criticized for their experimental design. The decision by the US Environmental Protection Agency to use one of these studies to set the oral reference dose led to discussions and objections from the industry [14].

To identify knowledge gaps and potential agreement or disagreement on the different aspects of the decaBDE issue a causal diagram illustrating scientists' current understanding of the cause-effect relationship between the production and use of decaBDE and its potential impact on health was made (See Figure 1). The diagram was based on the latest review articles and reports available and made similar to more brominated flame retardants.

A group of experts was asked to express their confidence in the current knowledge in the different parts of the diagram by completing an online questionnaire. From these experts a group of eight was selected to complete a second questionnaire and take part in an expert panel workshop where the implications of the results of the two different evaluations for policy and human health were discussed (Copenhagen 1905 2009). Priorities for further action were identified and the workshop aimed at arriving at a concrete expert advice for policy makers.

Assessment

Because of its wide use and environmental occurrence, preventing potential adverse effects on human health caused by decaBDE

is a task for authorities around the world. Taking appropriate political actions requires sufficient knowledge on the different aspects of chemicals, especially given the potential economic and safety consequences of a ban. The required weight of knowledge that is needed to support policy measures with regard to such issues is not well defined and open for debate amongst experts, policymakers and stakeholders. Both monitoring, modelling, epidemiological and experimental research are, however, quite time and money intensive. Therefore, the most important issues must be identified and prioritized.

Priority knowledge gaps

The top three most relevant areas to study further in order to assess the health impact of decaBDE were identified.

- In agreement with a recent review [3], the first area to prioritize is understanding better the magnitude of environmental transformation of decaBDE. The high abundance and temporal build-up measured in some environmental media are a cause for concern because of the evidences of transformational processes, resulting in more bioavailable and toxic BDEs [6]. If bromine is cleaved off from the decaBDE molecule in nature, the compound is transformed into lower brominated congeners which are already banned for their accumulating properties and toxic nature [6].
- Sources and magnitude of oral exposure is the 2nd prioritized area. There is too little knowledge on the extent of oral exposure in humans, from food and dust. There are data suggesting high exposure in children [9]. Monitoring of levels in humans, food and environment will provide a better insight in the main routes of exposure. It is also important to gain more knowledge on exposure in utero as the foetus may be more vulnerable than adults.
- The fate of the compound in the body is a third very important data gap relevant for human health risks posed by decaBDE. Toxicokinetics is the study of how a substance gets into the body and what happens to it in the body. The most important question is whether and to what degree decaBDE is metabolised in the human body to other more accumulating and toxic less brominated BDEs or readily excreted [1,8].

Also, toxicological health effects were considered an important area to prioritize. Some experts considered this to be among the top three priority areas.

There was disagreement amongst experts whether conducting more scientific research would yield <u>decisive</u> knowledge on the risks of decaBDE within the next five years. While most experts were either highly confident or meant that sufficient knowledge already exists, others claimed that high quality research requires more time.

Policy Considerations

<u>Arguments for using the precautionary principle to ban or restrict the use of decaBDE</u> would be the environmental abundance and increasing levels as described by Ross et al. 2008 combined with the uncertainties and potential threats in the priority areas described above and in recent reviews and reports [1,9]. Also, transport over long distances is indicated by the concentrations in remote areas, far away from production and use [3].The effects observed in animal studies involve brain development and hormone balance which are regarded highly relevant. A lesson is to be learned from other persistent organic pollutants where more sensitive endpoints were being detected at lower doses often at earlier life stages after initial assessment of high doses in adults on robust endpoints. There is also a risk that the most sensitive endpoints for decaBDE are still not detected. Then, the environmental load will have extensive consequences.

One expert pointed out that such a sensitive endpoint could be vitamin K metabolism and subsequent impacts on blood coagulation, as decaBDE has been reported to affect enzymes involved in this process [15,16]. Finally, one expert considered restrictions and prohibitions of the compound ethically justified, stating that it is unethical to pollute a whole population in order to prevent some fires.

<u>On the other hand, the existing knowledge does not necessitate a ban</u>, as few toxicological studies exist, and there is lack of knowledge regarding the margin of exposure; maybe the human exposure is not big enough for causing effects. The toxicological activity appears to be lower of decaBDE itself compared to BDEs with less bromines [9].

Another argument against a ban, is that the industry may take into use compounds that are less studied and have not been subjected to risk assessment [17]. However, for some uses, alternative compounds exist [17] which at least are not persistent.

Most experts had medium to high confidence in the possibility that policy actions to effectively manage the health risks of decaBDE will become technically (not politically) feasible within the next five years.

Based on the answers from the questionnaire and discussion at the workshop, the invited experts were not in agreement on whether or not the knowledge currently available is sufficient to justify more strict policy actions at this point. While most experts felt that the persistence of decaBDE and the transformation into bioaccumulating and toxic compounds are enough to justify a ban or restrictions on use, others felt that more data is required before a decision to change the status quo of this economically and technically important compound is justified.

Recommendations

There is a need for more research and monitoring of the substance to better support policy on this substance. Priority areas were defined as:

I. Environmental transformation of decaBDE into related lower brominated compounds with known abilities to accumulate in the body and to cause toxic effects
II. To what extent humans are exposed to decaBDE, in particular in utero, through food, mother's milk and dust.
III. The toxicokinetic properties of the compound, with special focus on the potential breakdown of decaBDE to the lower brominated BDEs and toxic metabolites in the human body.

Suggestions for improving knowledge could be:

I. To require more research and toxicological testing from the industry itself.
II. Better organised research cooperation between universities and research institutions at the European level
III. Better funding for relevant research.

There is a need for information on alternative substances.

Literature

1. U.S.Environmental Protection Agency. Toxicological review of decabromodiphenyl ether (BDE-209). EPA and In support of summary information on the integrated risk information system (IRIS). CAS No.1163-19-5. 2008. Washington D.C. Ref Type: Report
2. BSEF. Brominated Flame Retardant Deca-BDE Factsheet. 1-12-0009. BSEF. Ref Type: Report
3. Vonderheide, A. P., Mueller, K. E., Meija, J. & Welsh, G. L. Polybrominated diphenyl ethers: causes for concern and knowledge gaps regarding environmental distribution, fate and toxicity. Sci. Total Environ. 400, 425-436 (2008).
4. Court of Justice of the European Communities. Judgement of the Court (Grand Chamber) (Directive 2002/95/EC- Electrical and electronic equipment - Restriction of use of certain hazardous substances - Decabromodiphenyl ether ('DecaBDE') - Commission Decision 2005/717/EC - Exemption of DecaBDE from the prohibition on use - Actions for annulment - Commission's implementing powers - Infringement of the enabling provision). 1-4-2008. Ref Type: Report
5. Law, R. J. et al. Levels and trends of brominated flame retardants in the European environment. Chemosphere 64, 187-208 (2006).
6. Ross, P. S. et al. Large and growing environmental reservoirs of Deca-BDE present an emerging health risk for fish and marine mammals. Mar. Pollut. Bull. 58, 7-10 (2009).
7. Kajiwara, N., Noma, Y. & Takigami, H. Photolysis studies of technical decabromodiphenyl ether (DecaBDE) and ethane (DeBDethane) in plastics under natural sunlight. Environ. Sci. Technol. 42, 4404-4409 (2008).
8. Frederiksen, M., Vorkamp, K., Thomsen, M. & Knudsen, L. E. Human internal and external exposure to PBDEs--a review of levels and sources. Int. J. Hyg. Environ. Health 212, 109-134 (2009).
9. Costa, L. G. & Giordano, G. Developmental neurotoxicity of polybrominated diphenyl ether (PBDE) flame retardants. Neurotoxicology 28, 1047-1067 (2007).
10. Antignac, J. P. et al. Exposure assessment of French women and their newborn to brominated flame retardants: determination of tri- to deca- polybromodiphenylethers (PBDE) in maternal adipose tissue, serum, breast milk and cord serum. Environ. Pollut. 157, 164-173 (2009).
11. Stapleton, H. M. & Dodder, N. G. Photodegradation of decabromodiphenyl ether in house dust by natural sunlight. Environ. Toxicol. Chem. 27, 306-312 (2008).
12. Johansson, N., Viberg, H., Fredriksson, A. & Eriksson, P. Neonatal exposure to deca-brominated diphenyl ether (PBDE 209) causes dose-response changes in spontaneous behaviour and cholinergic susceptibility in adult mice. Neurotoxicology 29, 911-919 (2008).
13. Legler, J. New insights into the endocrine disrupting effects of brominated flame retardants. Chemosphere 73, 216-222 (2008).
14. Goodman, J. E. Neurodevelopmental effects of decabromodiphenyl ether (BDE-209) and implications for the reference dose. Regul. Toxicol. Pharmacol. 54, 91-104 (2009).
15. Pacyniak, E. K. et al. The flame retardants, polybrominated diphenyl ethers, are pregnane X receptor activators. Toxicol. Sci. 97, 94-102 (2007).
16. Bouwman, C. A., Seinen, W., Koppe, J. G. & van den, B. M. Effects of 2,3,7,8-tetrachlorodibenzo-p-dioxin or 2,2',4,4',5,5'-hexachlorobiphenyl on vitamin K-dependent blood coagulation in female germfree WAG/Rij-rats. Toxicology 75, 109-120 (1992).
17. European Chemicals Bureau, Institute for Health and Consumer Protection, Joint research Centre & European Commission. Review on production processes of decabromodiphenyl ether (DecaBDE) used in polymeric applications in electrical and electronic equipment, and assessment of the availability of potential alternatives to decaBDE. 2007. Ref Type: Report

Acknowledgements:

All experts attending the workshop are gratefully acknowledged, in alphabethical order: Åke Bergman[a], Lucio G Costa[b], Per Ola Darnerud[c], Marie Frederiksen[d], Helen Håkansson[e], Janna G Koppe[f], Jan L Lyche[g], Cathrine Thomsen[h] and Cynthia de Wit[a]. Also, all experts responding to the first questionnaire are acknowledged for their valuable and essential contribution.

[a] Stockholm University, Sweden

[b] University of Washington, US

[c] National Food Administration, Sweden

[d] University of Aarhus, Denmark

[e] Karolinska Institutet, Stockholm, Sweden

[f] Ecobaby Foundation, The Netherlands

[g] Norwegian School of Veterinary Science

[h] Norwegian Institute of Public Health

This HENVINET Policy Brief was produced by:

Author:

Karin E Zimmer[a] and Solveig Ravnum[b]

Contributors:

Hans Keune[c], Martin Krayer von Krauss[d], Erik Ropstad[a], Janneche U Skaare [a,b], Gunnar S Eriksen[b], Arno C Gutleb[e], Janna G Koppe[f], Albertinka J Murk[g], Brooke Magnanti[h], Alena Bartonova[i], Michael Kobernus[i] and Aileen Yang[i].

[a] Norwegian School of Veterinary Science
[b] National Veterinary Institute of Norway
[c] University of Antwerp, Belgium
[d] WHO Euro, Copenhagen, Denmark
[e] Centre de Recherche Public-Gabriel Lippmann, Luxembourg
[f] EcoBaby Foundation, The Netherlands
[g] Wageningen University, The Netherlands
[h] University Hospital, Bristol, UK
[i] NILU - Norwegian Institute for Air Research

Contact: Karin.Zimmer@nvh.no

Funding: This project was funded by the EU sixth Framework Programme as part of the HENVINET consortium.

NILU

Universiteit Antwerpen

Veterinærinstituttet
National Veterinary Institute

Norges veterinærhøgskole
