# Supplementary material for: Policy relevant Results from an Expert Elicitation on the Human Health Risks of Decabromodiphenyl ether (decaBDE) and Hexabromocyclododecane (HBCD)
Source: Environ Health. 2012 Jun 28;11(Suppl 1):S7. doi: 10.1186/1476-069X-11-S1-S7 (PMC3388476; doi:10.1186/1476-069X-11-S1-S7)
Supplement: Additional file 6_Policy Brief_HBCD — HENVINET Policy Brief. Expert Elicitation on Health Implications of HBCD. Based on the results from questionnaire 2 and the workshop, a policy recommendation was written as the final product of the project. [file 1476-069X-11-S1-S7-S6.pdf]

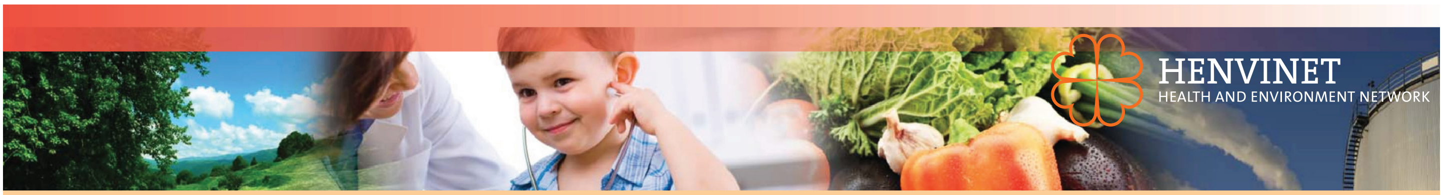

HENVINET Policy Brief:

Expert Elicitation on Health Implications of HBCD

Policy context

- HBCD is one of the major brominated flame retardants (BFRs) used today. BFRs are applied to prevent building materials, electronics, clothes and furniture from catching fire. The commercial formulation of HBCD contains three isomers: γ-HBCD, α-HBCD and β-HBCD.
- A sharp increase of the HBCD concentrations in the environment has been detected by several investigators since 2001, probably caused by the increased use of HBCD when other BFRs were banned or withdrawn (penta- and octabrominated diphenyl ether (PBDE) mixtures (Penta BDE, OctaBDE).
- The major concerns about HBCD are its persistence and its potential for bioaccumulation. The compound is found in high concentrations in both animals and nature.
- There are indications of toxicological effects of HBCD, especially in the liver and on the thyroid hormones. Also, once in the body, the different isomers of the technical mixture of HBCD are selectively metabolized. The α-HBCD isomer is metabolized at a slower rate and is accumulated to a greater extent in the body.
- On June 2nd 2009 the European Chemicals Agency (ECHA) within the REACH framework decided to restrict the use of HBCD within the EU such that it only can be used when "authorized" for specific purposes. HBCD is also currently proposed to be reviewed for a global agreement of restriction by the Stockholm Convention.
- Alternative substances to HBCD with putative lower risk have been proposed. Potential risks of these compounds are limited and further investigation is required.

Policy options

An expert workshop was conducted in order to evaluate the state of the current scientific knowledge and highlight important policy considerations.

Experts agreed that more information is needed about the HBCD compound in order to better understand its health impact. This requires more investment in fundamental science as well as certain policy measures such as monitoring activities.

Experts agreed to three priority areas for further investigation:

I. More knowledge, especially in humans, on the behavior of HBCD in the body, the mechanisms of action of HBCD and how HBCD may affect the health and illness of populations (toxicology and epidemiology).
II. More knowledge on the concentration levels of HBCD in the target tissues (absorption, distribution, metabolism and excretion of HBCD).
III. More knowledge on the extent of exposure to HBCD; especially human exposure and exposure to the general population.

Furthermore the following issues were proposed for better understanding:

I. The different behavior of the different HBCD stereo-isomers must also be addressed.
II. Effort should also be invested into research on the toxicity and environmental behaviour of the most frequently proposed alternatives to HBCD.
III. In order to accelerate the rate at which policy relevant information becomes available, experts feel that research collaborations between publically funded institutions should be organised at the European level.
IV. In addition to publically funded research, industry should be required to provide more toxicological data.
V. Policy makers must take decisions and invest more money in the required research.

Based on the answers from the questionnaire and discussion at the workshop, the invited experts were not in agreement on whether or not the knowledge currently available is sufficient to justify more strict policy actions at this point. While some experts considered the persistence and bioaccumulation properties of HBCD are enough to justify a ban or restrictions on use, others considered more data is required before a decision to change the status quo is justified.

Experts disagreed as to whether, given five years and adequate resources, additional research would yield decisive knowledge on the key issues related to HBCD and its alternatives. Experts had a medium to high degree of confidence in policy actions to effectively manage the health risks of HBCD to be technically (not necessarily politically) feasible either now, or within the next five years.

Executive summary

Situation

Brominated flame retardants (BFRs) are the major group of chemical flame retardants consisting of bromine containing organic compounds. BFRs are applied to prevent building materials, electronics, clothes and furniture from catching fire. Hexabromocyclododecane (HBCD or HBCDD) is one of the major BFRs. HBCD has 16 possible stereo-isomers with different biological activities, therefore the substance poses difficult problems for manufacturing, production and regulation [12]. The technical mixture/commercial formulation of HBCD contains three isomers: 75-89% γ-HBCD, 10-13% α-HBCD and 1-12% β-HBCD.

HBCD is used in construction and insulation boards, packaging material, electrical and electronic equipment, upholstered fabric and textiles, bed mattress, furniture, seatings, draperies, wall coverings, indoor textiles and automobile indoor textiles [12]. At present, according to BSEF, the brominated flame retardant industry panel, HBCD is the only suitable flame retardant for some of these applications.

The global production of HBCD was 16700 tons per year in 2001 and 23000 tons per year in 2008 [3]. This correlates well with a sharp increase of the HBCD concentrations in the environment detected by several investigators from 2001 onward [16], and is most probably caused by the increased use of HBCD when other BFRs were banned or withdrawed (penta- and octabrominated diphenyl ether (PBDE) mixtures (Penta BDE, OctaBDE). There is only one production site in Europe today, in the Netherlands.

HBCD's toxicity and harm to the environment is currently being discussed. The EU Risk Assessment (RA) of HBCD for environmental and human health was initiated in 1996 and finalized in 2008 [3,11,12]. The RA concluded that no risk to consumers was identified, and no risk for workers was identified when standard hygiene measures are applied. Further the RA concluded that HBCD has persistent, bioaccumulative and toxic (PBT) properties due to the reported increased environmental concentrations, the concerns linked to these higher concentrations, and the several specific risks identified in the aquatic environment. In June 2008 HBCD entered a screening procedure under the new legislation REACH [20]. On June 2nd 2009 the European Chemicals Agency (ECHA) within the REACH framework decided to restrict the use of HBCD within the EU such that it only can be used when “authorized” for specific purposes [9]. In Japan under the Chemical Substances Control Law (CSCL), HBCD was classified as a Type 1 Monitoring Chemical Substance since April 2004. The US Environmental Protection Agency (EPA) will finalize a review of HBCD in 2012. Canada will publish a risk assessment of HBCD during 2009. Furthermore, HBCD is currently proposed to be reviewed under the global framework of the Stockholm Convention on Persistent Organic Pollutants (POPs) [22]. HBCD is also included in the list of substances added to a proposal to revise the RoHS (Restriction of Hazardous Substances) directive [21].

Alternative substances to HBCD with putative lower risk have been proposed [10], but need further investigation. Among the proposed substances are: halogenated flame retardants in conjunction with antimony trixide, organic aryl phosphorous compounds, chlorinated paraffins, and ammonium polyphosphates.

Background

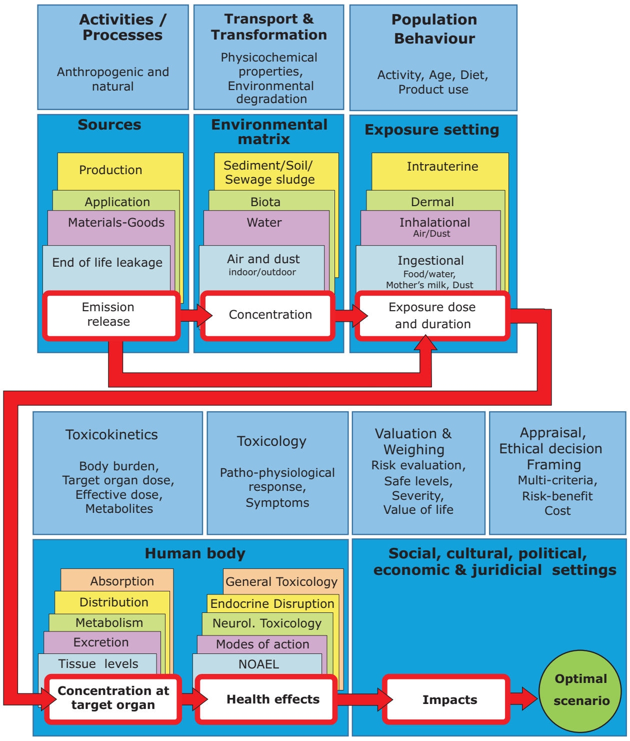

Figure 1. Diagram developed by HENVINET and used by expets to evaluate the current understanding of the cause-effect relationship between the production and use of HBCD and its potential impact on health. The diagram has been slightly adapted to comments from the experts.

HBCD is a ubiquitous contaminant in the environment, wildlife and humans due to widespread use, low volatility and low water solubility [6]. HBCD can be found in environmental samples such as birds, mammals, fish and other aquatic organisms as well as soil and sediment, but also in the anthroposphere. Humans can be exposed to HBCD by inhalation of vapor and airborne dust through ingestion and by dermal contact, babies can be exposed during pregnancy and breast feeding, workers and consumers are mainly exposed through inhalation and dermal routes and exposure in the environment occurs mainly via the oral route [12]. HBCD is easily taken up and stored by organisms, especially in adipose tissue. Animal studies have shown that from a technical mixture of HBCD the different isomers are selectively metabolized in the body so that the α-HBCD isomer is accumulated to a greater extent [5,6,12,26]. Also in nature a similar selective metabolism occurs mainly via microorganisms [7,8,13]. Animal studies have confirmed a low acute toxicity, but liver weights were increased, liver enzymes were induced, and thyroid hormone levels were affected [4,12,14,24,25]. We do not know anything about similar effects in humans. One recent Dutch study on human prenatal exposure to HBCD and other organohalogans suggest relationships on sexual and psychomotor development in healthy infants [17].

To identify knowledge gaps and potential agreement or disagreement on the different aspects of the HBCD issue, a causal diagram illustrating scientists' current understanding of the cause-effect relationship between the production and use of HBCD and its potential impact on health was made (See Figure 1). The diagram was based on the latest review articles and reports available.

A group of experts was asked to express their confidence in the current knowledge in the different parts of the diagram by completing an online questionnaire. From these experts a group of eight was selected to complete a second questionnaire and take part in an expert panel workshop where the implications of

the results of the two different evaluations for policy and health were discussed. Priorities for further action were identified and the workshop aimed at arriving at a concrete expert advice for policy makers.

Assessment

Our first step in developing an expert advice on HBCD for policy makers was focused on prioritizing the results from our expert consultation: how severe are specific results with regard to public health risks? The results were used to set priorities of further attention for policy uptake.

Priority knowledge gaps

The top area issues that the expert panel group considered to be the most influential for the health impact for HBCD was *toxicology* and *concentration in the target tissues* and *exposure*. Toxicology concerns the effects of a substance inside the body, and this area issue was ranked as number one. A request for more toxicological and epidemiological evaluation of the risk issue was raised. Concentration in the target tissues is a result of exposure and toxicokinetics, (more specifically what happens to the substance inside the body, how the substance is absorbed, distributed, metabolized and excreted). Toxicokinetics was ranked as number two. Exposure deals with the different routes of exposure, e.g. inhalation, ingestion, dermal.

Most experts in the panel had medium to very high confidence in science coming up with usable or decisive knowledge within the next five years if given sufficient resources. Most experts moreover had medium to high confidence in the possibility that policy actions to effectively manage the health risks of HBCD, will become technically (not politically) feasible within the next five years.

Weight of knowledge

During the expert panel discussions there was a general opinion that it is very difficult to be very certain about HBCD since there are less data available for this compound than for e.g. decaBDE. More specifically, there is a lack of epidemiological and toxicological studies, especially in humans [12]. There are limited data from toxicological studies of the targets of HBCD and of the mechanisms of action of HBCD. In addition there is very little information of the concentrations of HBCD in the target tissues, first of all due to lack of adequate studies on absorption, distribution, metabolism and excretion, but also because the different isomers of a technical mixture of HBCD are selectively metabolized in the body, so that α-HBCD is accumulated which behave differently from the original technical mixture [12,15,18,26]. It was also argued that there is a data gap on human exposure to HBCD, too little is known about normal exposure to the general population. Some exposure studies on children exist on sexual and psychomotor development in healthy infants [17] and estimations of exposure of occupational workers have been done [12]. Also the expert panel group considered that HBCD measurements performed in the past using the GC/MS technique are questionable compared to the LC/MS method used today [1,16].

Experts disagreed on the extent to which knowledge on the risks of HBCD justifies a more drastic policy intervention. On the basis of the persistence and bioaccumulation properties of HBCD, most experts suggested that policy makers should introduce regulations on restricting and prohibiting activities. Other experts felt that more data and better understanding are required before such drastic policy measures can be justified, they also claim that the use of suggested alternative compounds [10] is not proven to be safer, and developing safe alternatives take time. One expert considered restrictions and prohibitions of the compound ethically justified.

Some experts pointed out that studies performed on certain other persistent organic pollutants constitute a sufficient basis to justify, by analogy, concerns about the health effects of HBCD to humans. With these other chemicals, risk was first assessed at high doses in adults, but later more sensitive endpoints were detected at lower doses and often in earlier-life stages. One expert pointed out that one such endpoint could be vitamin K metabolism and subsequent impact on blood coagulation, and another endpoint could be leptin metabolism and possible impact on body weight [2,19,23]. Other experts do not agree in these conclusions based on the analogy to other persistent organic compounds.

It was suggested that in order to achieve what we want, more investment in fundamental science as well as policy measures such as monitoring activities is required.

It was claimed that there is no laboratory or institution in Europe where politicians and officers can initiate studies such as those within the US NTP program.

It was suggested to start randomized controlled trials of new medications or chemicals and to have permission from an ethical committee.

Based on the answers from the questionnaire and discussion at the workshop, the invited experts were not in agreement on whether or not the knowledge currently available is sufficient to justify more strict policy actions at this point. While most experts felt that the persistence and bioaccumulation properties of HBCD are enough to justify a ban or restrictions on use, others felt that more data is required before a decision to change the status quo is justified.

Recommendations

More research data and monitoring on HBCD is necessary to better support policy actions. The priority areas suggested were:

I. More research data and monitoring of epidemiological and toxicological studies of HBCD, especially in humans. Do randomized controlled trials and have permission from an ethical committee.
II. More research data and monitoring of the concentration of HBCD at the target tissue. Individual HBCD isomers need to be studied separately.
III. More research data and monitoring of exposure to HBCD, especially human exposure and exposure to the general population.

Suggestions for improving knowledge could be:

I. More research must be required from the industry itself that produces HBCD.
II. Better organized research, collaboration between universities and specific laboratories for required research studies.
III. Decisions taken and more money invested by policy makers in the required research.

Better information on safety of alternative substances is needed.

Literature

1. Abdallah, M. A. et al. Hexabromocyclododecane In Indoor Dust From Canada, the United Lingdom, and the United States. Environ. Sci. Technol. 42, 459-464 (2008).
2. Bouwman, C. A., Seinen, W., Koppe, J. G. & van den, B. M. Effects of 2,3,7,8-tetrachlorodibenzo-p-dioxin or 2,2',4,4',5,5'-hexachlorobiphenyl on vitamin K-dependent blood coagulation in female germfree WAG/Rij-rats. Toxicology 75, 109-120 (1992).
3. BSEF Fact Sheet, HBCD, Hexabromocyclododecane, http://www.bsef.com/uploads/Documents/documents/HBCD_factsheet.pdf. Brominated Science and Environmental Forum (BSEF, www. bsef. com) (2009).
4. Canton, R. F. et al. Subacute effects of hexabromocyclododecane (HBCD) on hepatic gene expression profiles in rats. Toxicol. Appl. Pharmacol. 231, 267-272 (2008).
5. Chengelis C.P. A 90-day oral (gavage) toxicity study of HBCD in rats. Wil Research Laboratories, Inc., Ashland, Ohio, USA. WIL-186012, pp1527. 1-1-2001. Ref Type: Generic
6. Covaci, A. et al. Hexabromocyclododecanes (HBCDs) in the environment and humans: a review. Environ. Sci. Technol. 40, 3679-3688 (2006).
7. Davis, J. W., Gonsior, S., Marty, G. & Ariano, J. The transformation of hexabromocyclododecane in aerobic and anaerobic soils and aquatic sediments. Water Res. 39, 1075-1084 (2005).
8. Davis, J. W. et al. Biodegradation and product identification of [14C]hexabromocyclododecane in wastewater sludge and freshwater aquatic sediment. Environ. Sci. Technol. 40, 5395-5401 (2006).
9. ECHA. ECHA Press Release, Helsinki, 02 June 2009, ECHA/PR/09/07. http://echa.europa.eu . 2-6-2009. Ref Type: Generic
10. ECHA_2 Data on manufacture, import, export, uses and releases of HBCDD as well as information on alternative substances. CAS No: 25637-99-4. ECHA_2008_2_SR04_HBCDD_report_12_01_2009.doc. http://echa. europa. eu (2008).
11. European Comission_2 Council Regulation 793/93/EEC of March 1993 on the evaluation and control of risks of existing substances. Official Journal of the European Communities 23 March 1993, (1993).
12. European Commission. Risk Assessment Report, Hexabromocyclododecane, CAS No:25637-99-4, R044_0805_env_hh_final_EBC.doc. http://ecb.jrc.ec.europa.eu/esis , 1-492. 1-5-2008. Ref Type: Generic
13. Gerecke, A. C. et al. Anaerobic degradation of brominated flame retardants in sewage sludge. Chemosphere 64, 311-317 (2006).
14. Germer, S. et al. Subacute effects of the brominated flame retardants hexabromocyclododecane and tetrabromobisphenol A on hepatic cytochrome P450 levels in rats. Toxicology 218, 229-236 (2006).
15. Hamers, T. et al. In vitro profiling of the endocrine-disrupting potency of brominated flame retardants. Toxicol. Sci. 92, 157-173 (2006).
16. Law, R. J. et al. Levels and trends of HBCD and BDEs in the European and Asian environments, with some information for other BFRs. Chemosphere 73, 223-241 (2008).
17. Meijer, L. et al. Serum concentrations of neutral and phenolic organohalogens in pregnant women and some of their infants in The Netherlands. Environ. Sci. Technol. 42, 3428-3433 (2008).
18. Palace, V. P. et al. Biotransformation enzymes and thyroid axis disruption in juvenile rainbow trout (Oncorhynchus mykiss) exposed to hexabromocyclododecane diastereoisomers. Environ. Sci. Technol. 42, 1967-1972 (2008).
19. Pelletier, C., Doucet, E., Imbeault, P. & Tremblay, A. Associations between weight loss-induced changes in plasma organochlorine concentrations, serum T(3) concentration, and resting metabolic rate. Toxicol. Sci. 67, 46-51 (2002).
20. REACH. Regulation (EC) No 1907/2006 of the European Parliament and the Council of 18th December 2006 concerning the Registration, Evaluation, Authorisation and Restricion of Chemicals (REACH). http://ec.europa.eu/environment/chemicals/reach/reach_intro.htm . 12-1-2006. Ref Type: Generic
21. RoHS Directive. List of substances added to a proposal to revise the RoHS (Restriction of Hazardous Substances) directive. http://www.rohs.gov.uk . 1-12-2008. Ref Type: Generic
22. Stockholm Convention on Persistant Organic Pollutants (POPs). Newly Proposed Chemicals. http://chm.pops.int . 5-12-2008. Ref Type: Generic
23. Tremblay, A. & Chaput, J. P. About unsuspected potential determinants of obesity. Appl. Physiol Nutr. Metab 33, 791-796 (2008).
24. van, d., V et al. Endocrine effects of hexabromocyclododecane (HBCD) in a one-generation reproduction study in Wistar rats. Toxicol. Lett. 185, 51-62 (2009).
25. van, d., V et al. A 28-day oral dose toxicity study enhanced to detect endocrine effects of hexabromocyclododecane in Wistar rats. Toxicol. Sci. 94, 281-292 (2006).
26. Zegers, B. N. et al. Levels of hexabromocyclododecane in harbor porpoises and common dolphins from western European seas, with evidence for stereoisomer-specific biotransformation by cytochrome p450. Environ. Sci. Technol. 39, 2095-2100 (2005).

Acknowledgements:

All experts attending the workshop are gratefully acknowledged: Åke Bergman[a], Lucio G Costa[b], Per Ola Darnerud[c], Marie Frederiksen[d], Helen Håkansson[e], Janna G Koppe[f], Jan L Lyche[g], Cathrine Thomsen[h] and Cynthia de Wit[a]. Also, all experts responding to the first questionnaire are acknowledged for their valuable and essential contribution.

[a] Stockholm University, Sweden

[b] University of Washington, US

[c] National Food Administration, Sweden

[d] University of Aarhus, Denmark

[e] Karolinska Institutet, Stockholm, Sweden

[f] Ecobaby Foundation, the Netherlands

[g] Norwegian School of Veterinary Science

[h] Norwegian Institute of Public Health

This HENVINET Policy Brief was produced by:

Authors:

Solveig Ravnum[a] and Karin E Zimmer[b]
[a] National Veterinary Institute of Norway
[b] Norwegian School of Veterinary Science

Contributors:

Martin Krayer von Krauss[c], Hans Keune[d], Erik Ropstad[b], Janneche U Skaare[a,b], Gunnar S Eriksen[a], Arno C Gutleb[e], Janna G Koppe[f], Albertinka J Murk[g], Brooke Magnanti[h], Alena Bartonova[i], Michael Kobernus[i] and Aileen Yang[i].
[a] National Veterinary Institute of Norway
[b] Norwegian School of Veterinary Science
[c] WHO Euro, Copenhagen, Denmark
[d] University of Antwerp, Belgium
[e] Centre de Recherche Public-Gabriel Lippmann, Luxembourg
[f] EcoBaby Foundation, the Netherlands
[g] Wageningen University, the Netherlands
[h] University Hospital Bristol, UK
[i] NILU - Norwegian Institute for Air Research

Contact: solveig.ravnum@vetinst.no

Funding: This project was funded by the EU sixth Framework Programme as part of the HENVINET consortium.

NILU Universiteit Antwerpen Veterinærinstituttet National Veterinary Institute Norges veterinærhøgskole
