# Supplementary material for: Policy relevant Results from an Expert Elicitation on the Human Health Risks of Decabromodiphenyl ether (decaBDE) and Hexabromocyclododecane (HBCD)
Source: Environ Health. 2012 Jun 28;11(Suppl 1):S7. doi: 10.1186/1476-069X-11-S1-S7 (PMC3388476; doi:10.1186/1476-069X-11-S1-S7)
Supplement: Additional file 7_Q1 Results_decaBDE and HBCD — Additional file 3_Q1 Results Available data from questionnaire 1 of decaBDE and HBCD. Mean, standard deviation, consensus measure and rank consensus are presented. [file 1476-069X-11-S1-S7-S7.pdf]

Additional file 7_Q1 Results

Ravnum et al 2010; Policy relevant Results from an Expert Elicitation on the Health Risks of Decabromo-diphenyl ether (decaBDE) and Hexabromocyclododecane (HBCD).

Summary of results from quesationnaire 1 on decaBDE and HBCD. The questions were "What is your level of confidence in the quality of the current scientific data on…" or "What is your level of confidence in the scientists' ability to predict…" followed by the text in the left side of the table. VH, H, M, L, VL = levels of confidence; No.Resp. = number of respondents; Std = standard deviation; CNS = consensus score (agreement); RANK (CNS) = consensus rank.

decaBDE	Questions	VH	H	M	L	VL	No. Resp.	Mean	Std	CNS	RANK (CONS)
	**Sources**	5	4	3	2	1					
S1	Production volumes	1	8	9	3	2	23	3,13	1,01	0,66	38
S2	Application volumes	0	5	10	6	2	23	2,78	0,90	0,70	30
S3	Identify&quantify all different applications	0	6	11	6	0	23	3,00	0,74	0,78	6
S4	Emission release leakage during production use&recycling	0	1	11	9	2	23	2,48	0,73	0,74	16
	**Environmental Matrix**										
EM1	Debromination& biological halflives	2	3	12	4	2	23	2,96	0,97	0,69	31
EM2	The magnitude of long range transport	1	6	14	2	0	23	3,26	1,02	0,78	7
EM3	Sediments	2	13	5	3	0	23	3,61	0,69	0,71	25
EM4	Sewage sludge	2	12	7	2	0	23	3,61	0,84	0,73	20
EM5	Soil	3	9	7	4	0	23	3,48	0,78	0,66	39
EM6	Water	2	4	8	6	3	23	2,83	0,95	0,59	57
EM7	Dust	4	4	11	3	1	23	3,30	1,15	0,63	45
EM8	Indoor Air	1	5	12	4	1	23	3,04	1,06	0,74	17
EM9	Outdoor Air	1	5	8	8	1	23	2,87	0,88	0,67	37
	**Level of exposure**										
EX1	The general population	0	2	15	6	0	23	2,83	0,58	0,83	2
EX2	Occupationally exposed	1	12	8	2	0	23	3,52	0,73	0,75	15
EX3	Infants and children	1	2	9	11	0	23	2,70	0,82	0,72	24
	**Main sources of exposure**										
EX4	The general population	0	3	12	8	0	23	2,78	0,67	0,78	8
EX5	Occupationally exposed	3	13	6	1	0	23	3,78	0,74	0,77	12

EX6	Infants and children	1	5	10	7	0	23	3,00	0,85	0,74	18
	**General population**										
EX7	Direct contactdermal	0	1	10	10	2	23	2,43	0,73	0,75	14
EX8	Inhalation	0	6	9	8	0	23	2,91	0,79	0,74	19
EX9	Ingestion	1	4	14	4	0	23	3,09	0,73	0,80	5
	**Occupational exposed**										
EX10	Direct contact dermal	0	3	15	5	0	23	2,91	0,60	0,84	1
EX11	Inhalation	4	8	10	1	0	23	3,65	0,83	0,71	27
EX12	Ingestion	4	3	11	4	1	23	3,22	1,09	0,63	46
	**Infants and children**										
EX13	Direct contact dermal	0	1	8	13	1	23	2,39	0,66	0,77	10
EX14	Inhalation	1	3	10	6	3	23	2,70	1,02	0,65	42
EX15	Intrauterine	1	1	8	8	5	23	2,35	1,03	0,63	43
EX16	Via food	1	9	8	5	0	23	3,26	0,86	0,70	29
EX17	Via breast milk	2	14	4	2	1	23	3,61	0,94	0,68	34
	**Toxicokinetics**										
TK01	Absorbed/taken up	1	2	13	7	0	23	2,87	0,76	0,78	9
TK02	Metabolised to hydroxymetabolites after absorption	0	0	14	7	2	23	2,52	0,67	0,77	13
TK03	Debrominated to lower ominated congeners after absorption	1	2	7	11	2	23	2,52	0,95	0,67	36
TK04	Debrominated or metabolised by the intestinal microflora	0	0	7	15	1	23	2,26	0,54	0,82	3
TK05	Accumulating in the body	1	9	10	3	0	23	3,35	0,78	0,73	21
TK06	Excreted via bile and faeces	2	7	6	7	1	23	3,09	1,08	0,61	49
TK07	Excreted via urine	2	5	5	8	3	23	2,78	1,20	0,55	58
TK08	Distribution to different tissues	2	4	12	4	1	23	3,09	0,95	0,71	26
TK09	Final concentration of the parent compound in the target tissues	0	3	8	11	1	23	2,57	0,79	0,72	23
TK10	Final metabolite concentration in target tissues	0	1	3	10	9	23	1,83	0,83	0,73	22
TK11	The biological halflife	1	5	9	6	2	23	2,87	1,01	0,66	40
	**Human Epidemiological studies**										
TX01	Males	0	1	5	7	9	22	1,91	0,92	0,68	33
TX02	Females	0	1	4	7	10	22	1,82	0,91	0,69	32
	**General health in**										
TX03	Males	1	2	10	5	4	22	2,59	1,05	0,63	47
TX04	Females	1	2	10	5	4	22	2,59	1,05	0,63	48
	**Neurodevelopment**										
TX05	Males exposed during fetal or neonatal life	2	2	8	7	3	22	2,68	1,13	0,60	55
TX06	Females exposed during fetal or neonatal life	2	1	9	6	4	22	2,59	1,14	0,59	56

	**Thyroid function in**										
TX07	Males exposed as adults	1	6	8	6	1	22	3,00	0,98	0,68	35
TX08	Females exposed as adults	1	6	8	5	2	22	2,95	1,05	0,65	41
TX09	Males exposed during foetal or neonatal life	1	3	8	6	4	22	2,59	1,10	0,60	50
TX10	Females exposed during foetal or neonatal life	1	3	8	6	4	22	2,59	1,10	0,60	51
	**Reproductive function in**										
TX11	Males exposed as adults	0	4	8	6	4	22	2,55	1,01	0,63	44
TX12	Females exposed as adults	1	3	8	6	4	22	2,59	1,10	0,60	52
TX13	Males exposed during foetal or neonatal life	1	3	7	7	4	22	2,55	1,10	0,60	53
TX14	Females exposed during foetal or neonatal life	1	3	7	7	4	22	2,55	1,10	0,60	54
	**Knowledge of the mechanisms of actions**										
TX15	BDE209	0	2	4	15	1	22	2,32	0,72	0,77	11
TX16	Metabolites of BDE209	0	2	1	14	5	22	2,00	0,82	0,80	4
TX17	NOAEL of BDE209	0	2	8	9	3	22	2,41	0,85	0,70	28

**HBCD**	**Questions**	**VH**	**H**	**M**	**L**	**VL**	**No. Resp.**	**Mean**	**Std**	**CNS**	**RANK (CONS)**
	**Sources**	5	4	3	2	1					
S01	Production volumes	2	4	5	2	0	13	3,46	0,97	0,66	48
S02	Application volumes	1	4	6	2	0	13	3,31	0,85	0,71	25
S03	Identify&quantify all different applications	0	7	4	2	0	13	3,38	0,77	0,73	19
S04	Emission release leakage during production use&recycling	0	1	6	5	1	13	2,54	0,78	0,73	17
	**Environmental Matrix**										
EM01	Debromination & biological halflives	0	3	9	1	0	13	3,15	0,55	0,84	1
EM02	The magnitude of longrange transport	0	3	4	6	0	13	2,77	0,83	0,71	26
EM03	Sediments	2	5	5	1	0	13	3,62	0,87	0,70	30
EM04	Sewage sludge	2	6	2	3	0	13	3,54	1,05	0,62	53
EM05	Soil	0	5	6	1	1	13	3,15	0,90	0,72	22
EM06	Water	0	4	5	3	1	13	2,92	0,95	0,69	33
EM07	Dust	1	3	4	5	0	13	3,00	1,00	0,67	42
EM08	Indoor Air	1	2	6	4	0	13	3,00	0,91	0,73	18
EM09	Outdoor Air	0	3	2	6	2	13	2,46	1,05	0,62	54
	**Level of exposure**										
EX01	The general population	0	2	6	5	0	13	2,77	0,73	0,76	7

EX02	Occupationally exposed	2	3	6	2	0	13	3,38	0,96	0,67	41
EX03	Infants and children	0	1	6	4	2	13	2,46	0,88	0,69	31
	**Main sources of exposure**										
EX04	The general population	1	3	5	4	0	13	3,08	0,95	0,69	34
EX05	Occupationally exposed	4	2	6	1	0	13	3,69	1,03	0,62	57
EX06	Infants and children	0	4	5	3	1	13	2,92	0,95	0,69	35
EX07	Direct contactdermal	0	2	4	5	2	13	2,46	0,97	0,66	49
EX08	Inhalation	1	1	5	6	0	13	2,77	0,93	0,69	32
EX09	Ingestion	0	4	5	4	0	13	3,00	0,82	0,74	11
	**Occupational exposed**										
EX10	Direct contact dermal	1	3	4	5	0	13	3,00	1,00	0,67	43
EX11	Inhalation	3	2	5	3	0	13	3,38	1,12	0,59	63
EX12	Ingestion	1	3	4	4	1	13	2,92	1,12	0,62	58
	**Infants and children**										
EX13	Direct contact dermal	0	1	3	7	2	13	2,23	0,83	0,74	16
EX14	Inhalation	1	1	4	5	2	13	2,54	1,13	0,60	61
EX15	Intrauterine	1	0	6	5	1	13	2,62	0,96	0,68	36
EX16	Via food	0	3	5	2	3	13	2,62	1,12	0,59	62
EX17	Via breast milk	1	5	3	4	0	13	3,23	1,01	0,63	51
	**Toxicokinetics**										
TK01	Absorbed/taken up	0	5	9	3	1	18	3,00	0,84	0,76	8
TK02	Metabolised to diastereomers after absorption	0	4	5	8	1	18	2,67	0,91	0,68	37
TK03	Metabolised to hydroxymetabolites	0	2	5	9	2	18	2,39	0,85	0,71	27
TK04	Metabolised to debrominated metabolites	0	0	6	8	4	18	2,11	0,76	0,76	9
TK05	Accumulating in the body	0	6	9	3	0	18	3,17	0,71	0,77	4
TK06	Excreted via bile and faeces	0	5	8	5	0	18	3,00	0,77	0,77	6
TK07	Excreted via urine	0	4	7	6	1	18	2,78	0,88	0,70	29
TK08	Distribution to different tissues	0	5	12	1	0	18	3,22	0,55	0,83	2
TK09	Final concentration of the parent compound in the target tissues	0	5	9	2	2	18	2,94	0,94	0,72	23
TK10	Final concentration of metabolites in the target tissues	0	0	8	6	4	18	2,22	0,81	0,72	24
TK11	The biological halflife	0	5	7	5	1	18	2,89	0,90	0,70	28
	**Human Epidemiological studies**										
TX01	Males	0	3	9	5	1	18	2,78	0,81	0,74	14
TX02	Females	0	3	9	5	1	18	2,78	0,81	0,74	15
	**General health in**										
TX03	Males	1	1	3	10	3	18	2,28	1,02	0,67	44
TX04	Females	1	1	3	10	3	18	2,28	1,02	0,67	45
	**Nervous system**										
TX05	Males exposed as adults	1	2	3	9	3	18	2,39	1,09	0,62	59

TX06	Females exposed as adults	1	2	3	9	3	18	2,39	1,09	0,62	60
TX07	Males exposed during foetal or neonatal life	1	1	7	7	2	18	2,56	0,98	0,66	46
TX08	Females exposed during foetal or neonatal life	1	1	7	7	2	18	2,56	0,98	0,66	47
	**Thyroid function**										
TX09	Males exposed as adults	1	5	6	4	2	18	2,94	1,11	0,62	55
TX10	Females exposed as adults	1	5	6	4	2	18	2,94	1,11	0,62	56
TX11	Males exposed during foetal or neonatal life	0	2	9	6	1	18	2,67	0,77	0,74	13
TX12	Females exposed during foetal or neonatal life	0	3	8	6	1	18	2,72	0,83	0,72	20
	**Reproductive function**										
TX13	Males exposed as adults	0	4	5	8	1	18	2,67	0,91	0,68	38
TX14	Females exposed as adults	0	4	5	8	1	18	2,67	0,91	0,68	39
TX15	Males exposed during foetal or neonatal life	1	2	5	8	2	18	2,56	1,04	0,63	52
TX16	Females exposed during foetal or neonatal life	1	2	5	9	1	18	2,61	0,98	0,66	50
	**Knowledge of the mechanisms of actions**										
TX17	HBCD	0	1	6	8	3	18	2,28	0,83	0,72	21
TX18	a- HBCD	0	2	1	10	5	18	2,00	0,91	0,75	10
TX19	b-HBCD	0	1	1	9	7	18	1,78	0,81	0,74	12
TX20	g-HBCD	0	1	2	10	5	18	1,94	0,80	0,77	5
TX21	Other metabolites of HBCD	0	0	1	7	10	18	1,50	0,62	0,78	3
TX22	NOAEL of HBCD	0	4	3	10	1	18	2,56	0,92	0,67	50
